# Understanding complex dynamics of behavioral, neurochemical and transcriptomic changes induced by prolonged chronic unpredictable stress in zebrafish

**DOI:** 10.1038/s41598-020-75855-3

**Published:** 2020-11-17

**Authors:** Konstantin A. Demin, Anton M. Lakstygal, Nataliya A. Krotova, Alexey Masharsky, Natsuki Tagawa, Maria V. Chernysh, Nikita P. Ilyin, Alexander S. Taranov, David S. Galstyan, Ksenia A. Derzhavina, Nataliia A. Levchenko, Tatiana O. Kolesnikova, Mikael S. Mor, Marina L. Vasyutina, Evgeniya V. Efimova, Nataliia Katolikova, Andrey D. Prjibelski, Raul R. Gainetdinov, Murilo S. de Abreu, Tamara G. Amstislavskaya, Tatyana Strekalova, Allan V. Kalueff

**Affiliations:** 1 Institute of Translational Biomedicine, St. Petersburg State University, University Emb. 7-9, Saint-Petersburg, Russia; 2 Institute of Experimental Medicine, Almazov National Medical Research Centre, Ministry of Healthcare of Russian Federation, Saint-Petersburg, Russia; 3 Core Facility Centre for Molecular and Cell Technologies, St. Petersburg State University, Saint-Petersburg, Russia; 4 Department of Biophysics and Biochemistry, Graduate School of Science, University of Tokyo, Tokyo, Japan; 5 Laboratory of Preclinical Bioscreening, Granov Russian Research Center of Radiology and Surgical Technologies, Ministry of Healthcare of Russian Federation, Pesochny, Russia; 6 School of Pharmacy, Southwest University, Chongqing, China; 7 Institute of Cytology RAS, Saint-Petersburg, Russia; 8 Center for Algorithmic Biotechnology, Institute of Translational Biomedicine, St. Petersburg State University, Saint-Petersburg, Russia; 9 University of Passo Fundo, Passo Fundo, Brazil; 10 Laboratory of Biopsychiatry, Scientific Research Institute of Physiology and Basic Medicine, Novosibirsk, Russia; 11 School of Medicine and Psychology, Novosibirsk State University, Novosibirsk, Russia; 12 Department of Psychiatry and Neuropsychology, Maastricht University, Maastricht, The Netherlands; 13 Laboratory of Psychiatric Neurobiology, Institute of Molecular Medicine, I.M. Sechenov First Moscow State Medical University, Moscow, Russia; 14 Division of Molecular Psychiatry, Centre of Mental Health, University of Würzburg, Würzburg, Germany; 15 Ural Federal University, Ekaterinburg, Russia; 16 Laboratory of Cell and Molecular Biology and Neurobiology, Moscow Institute of Physics and Technology, Dolgoprudny, Russia

**Keywords:** Diseases of the nervous system, Emotion, Stress and resilience, Neurochemistry, Transcriptomics

## Abstract

Stress-related neuropsychiatric disorders are widespread, debilitating and often treatment-resistant illnesses that represent an urgent unmet biomedical problem. Animal models of these disorders are widely used to study stress pathogenesis. A more recent and historically less utilized model organism, the zebrafish (*Danio rerio*), is a valuable tool in stress neuroscience research. Utilizing the 5-week chronic unpredictable stress (CUS) model, here we examined brain transcriptomic profiles and complex dynamic behavioral stress responses, as well as neurochemical alterations in adult zebrafish and their correction by chronic antidepressant, fluoxetine, treatment. Overall, CUS induced complex neurochemical and behavioral alterations in zebrafish, including stable anxiety-like behaviors and serotonin metabolism deficits. Chronic fluoxetine (0.1 mg/L for 11 days) rescued most of the observed behavioral and neurochemical responses. Finally, whole-genome brain transcriptomic analyses revealed altered expression of various CNS genes (partially rescued by chronic fluoxetine), including inflammation-, ubiquitin- and arrestin-related genes. Collectively, this supports zebrafish as a valuable translational tool to study stress-related pathogenesis, whose anxiety and serotonergic deficits parallel rodent and clinical studies, and genomic analyses implicate neuroinflammation, structural neuronal remodeling and arrestin/ubiquitin pathways in both stress pathogenesis and its potential therapy.

## Introduction

Stress evokes multiple behavioral and physiological responses^1,2^, including neuroendocrine and immune deficits^3–7^, that may trigger common affective illnesses, such as anxiety, depression and post-traumatic stress disorder (PTSD)^8–11^. Widespread, debilitating and often treatment-resistant, these neuropsychiatric disorders represent an urgent unmet biomedical problem^12–14^, complicated by their multiple overlapping genetic and environmental determinants, and poor understanding of their mechanisms and risk factors^15,16^. Animal models, especially employing rodents, are widely used to study stress biology and pathogenesis^17–19^. Commonly utilizing various chronic unpredictable stress (CUS) protocols^20–24^, such models typically expose rodents to continuous exposure to varying stressors for several weeks^22,24–27^, to evoke anxiety- and/or depression-like states^28–30^ and physiological alterations that resemble those observed clinically^31^.

**Figure 1:**
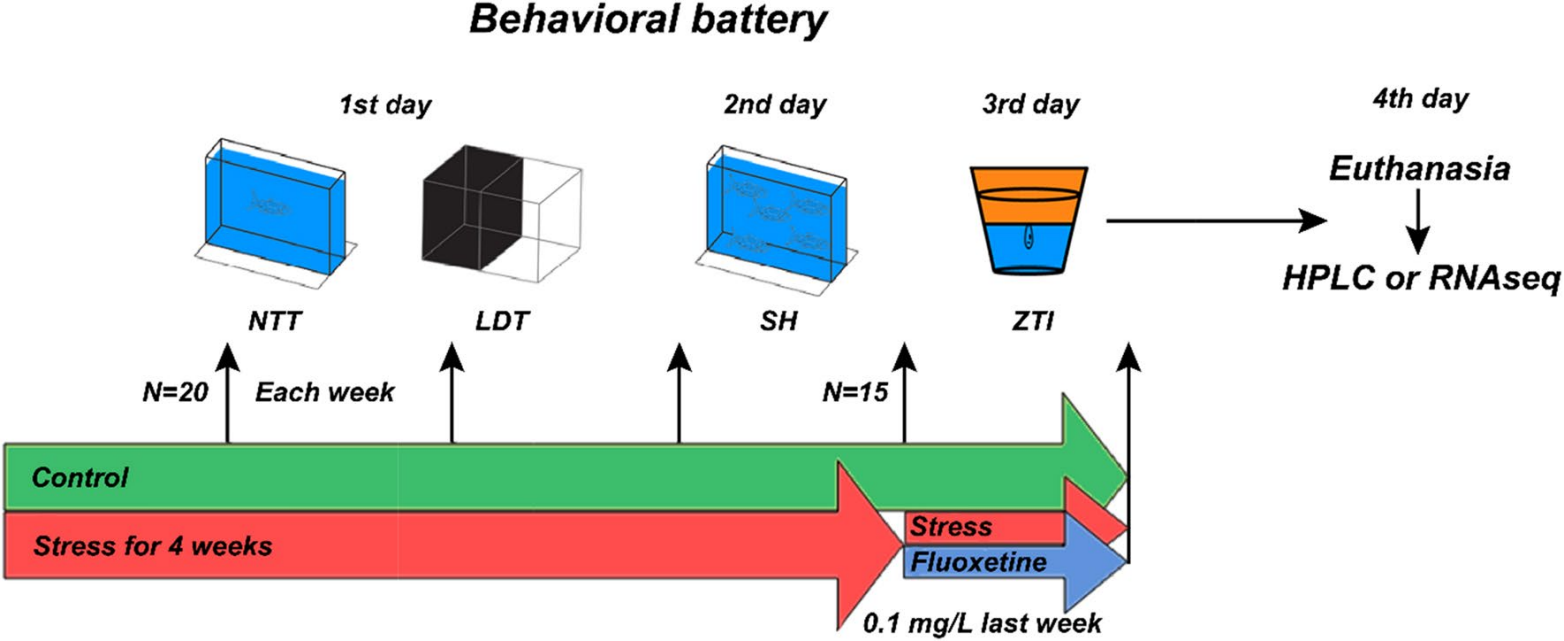
A brief diagram outlining the study experimental design, including the chronic unpredictable stress (CUS) protocol and behavioral testing (see Table 1 for details of CUS stressors applied in the present study). *NTT* the novel tank test, *LDT* the light–dark test, *SH* shoaling test, *ZTI* the zebrafish tail immobilization test, *HPLC* high-performance liquid chromatography, *RNASeq* RNA sequencing.

A more recently recognized and historically less utilized model organism, the zebrafish (*Danio rerio*) is rapidly becoming a critical species for translational neuroscience research, complementing rodent studies^32,33^. For example zebrafish demonstrate high genetic and physiological similarity to humans^34,35^, and possess major, evolutionarily conserved neurotransmitter systems^36,37^ and shared central nervous system (CNS) morphology^38,39^. Zebrafish are also widely used in stress research^40–42^, often based on various aquatic CUS protocols adapted from rodent models^43–47^. Here, we utilize a rigorous 5-week CUS protocol, already established in our laboratory^48^, to examine brain transcriptomic changes and weekly dynamics of behavioral and neurochemical stress responses in adult zebrafish (Fig. 1, Table 1), as well as their potential correction by fluoxetine, a common, clinically efficient and most prescribed selective serotonin reuptake inhibitor (SSRI) antidepressant.

**Table 1:** A brief summary of the chronic unpredictable stress (CUS) protocol used in the present study (adapted from^48^).

CUS days	Specific CUS stress procedures	Behavioral tests and sampling
1	Tank change → local hypothermia*	
2	Net chasing (10 min) → novel objects added to the hometank **(6 h)	
3	Crowding (2 h) → Air exposure ***	
4	Darkness (24 h)	
5	Food deprivation (24 h)	
6	Water change → noise exposure in unfamiliar tank **** (4 h)	
7	Alarm pheromone ***** → remixing cohorts	Week 1 NTT/LDT
8	Bright light (4 h) → novel objects (8 h)	ST
9	Crowding (2 h) + remixing cohorts → net chasing (15 min)	ZTI
10	Noise exposure in unfamiliar tank **** (5 h) → water change	Euthanasia, sampling
11	Darkness (24 h)	
12	Food deprivation (24 h)	
13	Air exposure → local hypothermia	
14	Bright light (6 h) → alarm pheromone	Week 2 NTT/LDT
15	Noise exposure in an unfamiliar tank (6 h) → remixing cohorts	ST
16	Novel objects (10 h) → shallow water (8 h) + water change	ZTI
17	Crowding (3 h) + remixing cohorts → alarm pheromone	Euthanasia, sampling
18	Darkness (24 h)	
19	Food deprivation (24 h)	
20	Net chasing (20 min) → local hypothermia	
21	Bright light (8 h) → alarm pheromone	Week 3 NTT/LDT
22	Overcrowding (12 h) → remixing cohorts	ST
23	Exposure to air → shallow water (10 h) + water change	ZTI
24	Noise exposure in unfamiliar tank (8 h) → alarm pheromone	Euthanasia, sampling
25	Darkness (24 h)	
26	Food deprivation (24 h)	
27	Bright light (12 h) → shallow water (12 h)	
28	Tank change + remixing cohorts → air exposure	Week 4 NTT/LDT
29	Food deprivation (24 h) → local hypothermia	ST
30	Bright light (14 h) → net chasing (30 min)	ZTI
31	Alarm pheromone → novel objects (18 h)	Euthanasia, sampling
32	Darkness (24 h)	
33	Food deprivation (24 h)	
34	Noise exposure in unfamiliar tank (10 h) → overcrowding (12 h)	
35	–	Week 5 NTT/LDT
36	–	ST
37	–	ZTI
38	–	Euthanasia, sampling

Note that fish undergoing 3-day behavioral testing on specific weeks 1–5 were separated from stress cohort, excluded from CUS procedures after behavioral assays, and euthanized for sampling next day after the last behavioral test of the battery (ZTI) was administered.

*NTT* the novel tank test, *LDT* the light/dark test, *ST* shoaling test, *ZTI* the zebrafish tail immobilization test.

*Adding 0.5 L of ice to the hometank.

**Adding 20 novel plastic children kinder toys to the hometank.

***Simultaneous exposure of all cohort to air in the net (30 s, followed by a 1-min rest, repeated 5 times).

****Exposure to loud white noise sound using the 40-W sound speakers.

*****Exposure to 5 mL alarm pheromone (per L of water) extracted from additional intact fish as in^40, 68^.

## Results

The generalized linear models 1 (GZLM1) analyses were used in the present study to compare CUS and control zebrafish groups in the novel tank test (NTT) across all 5 weeks of stress, revealing significant Wald test effects for week, group for distance traveled, as well as the time spent in top, with significant within-week differences between control and stressed fish in all five experimental weeks (Table 2, Supplementary Tables S2–S4). There were also significant group, week and week × group effects for time spent not moving, where the post-hoc Tukey’s test revealed only the significant difference within week group (control vs. stress) for week 2, but not other weeks (Table 2, Supplementary Tables S2–S4). We also found significant week and interaction, but not group, effects for the number of top entries, as Tukey’s test for week groups revealed significant differences vs. control only at week 1 (Table 2, Supplementary Tables S2–S4). Finally, significant week, group and interaction effects were found for the latency to enter the top, whose post-hoc pairwise week comparisons revealed significant differences from controls in weeks 2, 3 and 5 (Table 2, Supplementary Tables S2–S4).

**Table 2:** Summary of the Wald Chi-square test results (ANOVA Type II) for generalized linear model (GZLM1; Supplementary Table S2) using week (1–5), group (control vs. stress) and their interaction effects as predictors (GZLM1) in the zebrafish 5-week chronic unpredictable stress (CUS) model used in the present study.

Factors	Df	χ^2^	P	Factors	Df	χ^2^	p
**NTT distance travelled, cm**				**NTT time spent not moving, s**			
Week	4.00	11.12	0.03	Week	4.00	21.09	< 0.01
Group	1.00	52.03	< 0.01	Group	1.00	9.51	< 0.01
Week × group	4.00	9.52	NS	Week × Group	4.00	11.95	0.02
**NTT time spent in top, s**				**NTT number of top entries**			
Week	4.00	148.85	< 0.01	Week	4.00	14.01	0.01
Group	1.00	149.59	< 0.01	Group	1.00	0.25	NS
Week × group	4.00	11.76	0.02	Week × group	4.00	18.33	< 0.01
**NTT latency to enter top, s**				**ZTI time spent active, s**			
Week	4.00	17.63	< 0.01	Week	4.00	10.67	0.03
Group	1.00	27.06	< 0.01	Group	1.00	0.60	NS
Week × group	4.00	10.38	0.03	Week × group	4.00	19.80	< 0.01
**LDT time spent in light, s**				**LDT number of light entries**			
Week	4.00	2.45	NS	Week	4.00	6.50	NS
Group	1.00	31.54	< 0.01	Group	1.00	2.47	NS
Week × group	4.00	17.11	< 0.01	Week × group	4.00	2.70	NS
**ST distance to surface, cm**				**ST inter-fish distance, cm**			
Week	4.00	12.26	0.02	Week	4.00	5.36	NS
Group	1.00	1.31	NS	Group	1.00	12.76	< 0.01
Week × group	4.00	34.86	< 0.01	Week × group	4.00	5.46	NS
**Dopamine, pg/mg**				**DOPAC/dopamine ratio**			
Week	4.00	11.02	0.03	Week	4.00	10.67	0.03
Group	1.00	0.88	NS	Group	1.00	0.37	NS
Week × group	4.00	2.77	NS	Week × group	4.00	3.80	NS
**DOPAC, pg/mg**				**5-HIAA, pg/mg**			
Week	4.00	3.51	NS	Week	4.00	7.19	NS
Group	1.00	0.40	NS	Group	1.00	2.52	NS
Week × group	4.00	3.40	NS	Week × group	4.00	14.73	0.01
**5-HIAA/serotonin ratio**				**Serotonin, pg/mg**			
Week	4.00	6.53	NS	Week	4.00	4.23	NS
Group	1.00	2.70	NS	Group	1.00	0.54	NS
Week × group	4.00	18.22	< 0.01	Week × group	4.00	5.67	NS
**HVA/dopamine ratio**				**HVA, pg/mg**			
Week	4.00	7.06	NS	Week	4.00	3.47	NS
Group	1.00	2.59	NS	Group	1.00	2.34	NS
Week × group	4.00	5.18	NS	Week × group	4.00	5.97	NS
**Norepinephrine, pg/mg**							
Week	4.00	13.76	0.01				
Group	1.00	3.79	NS				
Week × group	4.00	4.56	NS				

**Figure 2:**
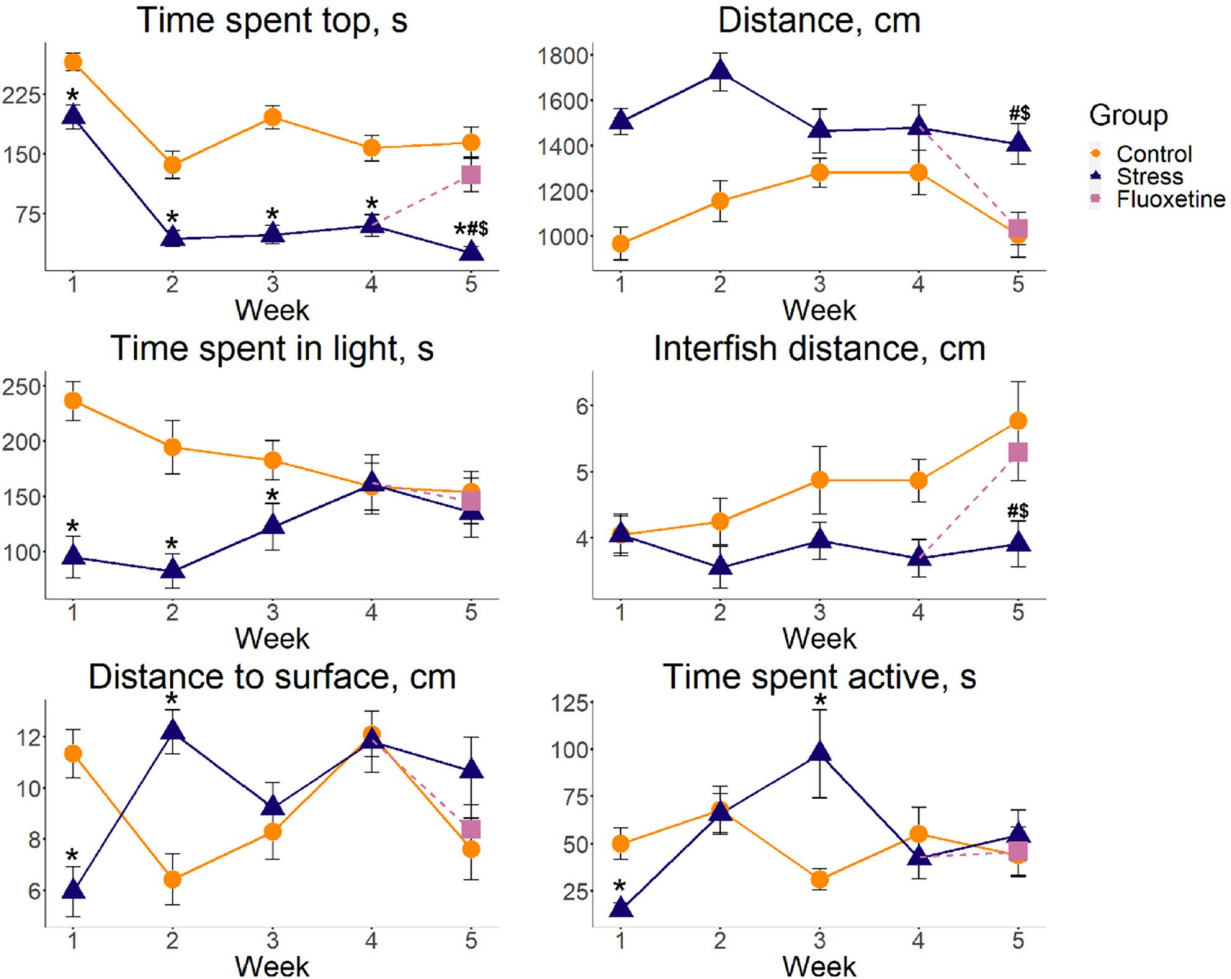
Weekly dynamics of behavioral alterations induced by chronic unpredictable stress (CUS) exposure and fluoxetine treatment in adult zebrafish tested in the novel tank test (time spent in top and distance traveled), the light–dark test (time spent in light), shoaling test (average inter-fish distance and distance to water surface) and the zebrafish tail immobilization test (ZTI, time spent active). Data is represented as mean ± S.E.M. (n = 20 in weeks 1–3 and n = 15 in weeks 4–5), *p < 0.05 control vs. stress, post-hoc Tukey’s test for significant Wald Chi-squared test (ANOVA Type II) for GZLM1 for group (control and stress), week (1–5) and their interaction as predictors, ^#^p < 0.05 stress vs. control group and ^$^p < 0.05 fluoxetine vs. stress group, post-hoc Tukey’s test for significant Wald Chi-squared test ANOVA (Type II) for GZLM2 for group (control, stress and fluoxetine) at week 5 as predictor. Graphs were constructed using the ggplot2 R package^137^, also see Tables 2 and 3 and Supplementary Tables S1–S5 for statistical details.

**Figure 3:**
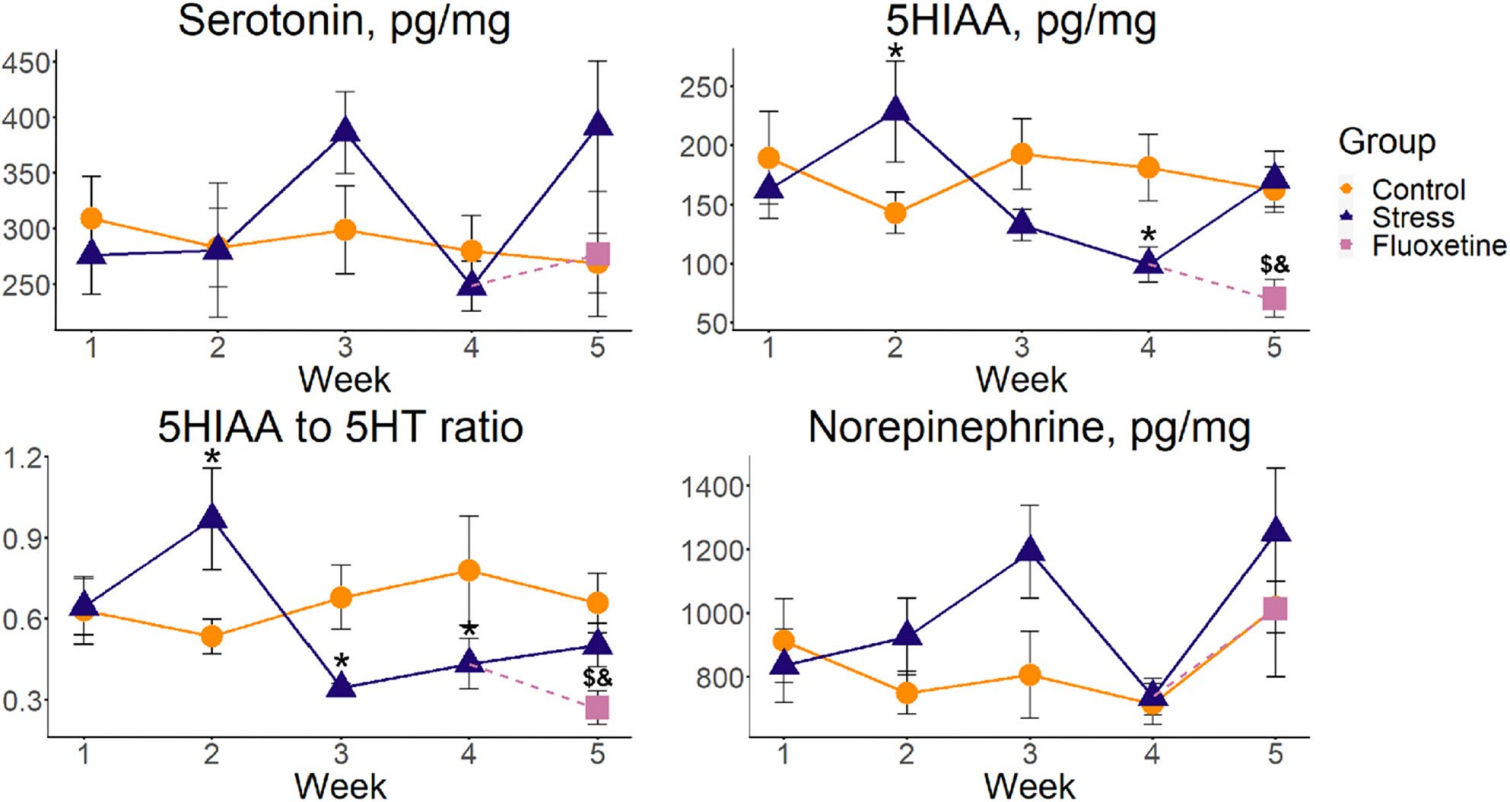
Weekly dynamics of neurochemical alterations induced by chronic unpredictable stress (CUS) exposure and fluoxetine treatment, assessed by HPLC in the whole-brain samples of adult zebrafish (n = 10). Data are represented as mean ± S.E.M. *p < 0.05 control vs. stress, post-hoc Tukey’s test for significant Wald Chi-squared test ANOVA (Type II) for GZLM1 for group (control and stress), week (1–5) and their interaction as predictors, ^#^p < 0.05 stress vs. control group and ^$^p < 0.05 fluoxetine vs. stress group and ^&^p < 0.05 fluoxetine vs. control, post-hoc Tukey’s test for significant Wald Chi-squared test ANOVA (Type II) for GZLM2 using group (control, stress and fluoxetine) at week 5 as predictor. Graphs were constructed using the ggplot2 R package^137^, also see Tables 2 and 3 and Supplementary Tables S1–S5 for statistical details.

Behavioral endpoints were assessed in the novel tank test (NTT), light–dark test (LDT), shoaling test (ST) and the zebrafish tail immobilization test (ZTI), also see Figs. 2 and 3, Table 3 and Supplementary Tables S1–S5 for details.

*NS* no significant differences (p > 0.05), *DOPAC* 3,4-dihydroxyphenylacetic acid, *5-HIAA* 5-hydroxyindoleacetic acid, *HVA* homovanillic acid. *Df* degree of freedom.

Generalized linear models 2 (GZLM2) analyses of CUS, fluoxetine and control groups at week 5 revealed significant NTT group effect for distance travelled, with significant differences between stress vs. both control and fluoxetine by post-hoc Tukey’s test (Table 3). There was also significant group effect for time spent in top, with significant differences in control vs. stress and vs. fluoxetine (Table 3). Finally, significant group effect was found for the latency to enter the top, yielding significant stress vs. control and vs. fluoxetine differences, as assessed by the post-hoc Tukey’s test (Table 3).

**Table 3:** Summary of post-hoc Tukey’s test results for significant Wald Chi-square test (ANOVA Type II) for generalized linear model (GZLM1; Supplementary Table S6) using group (control, stress and fluoxetine) at week 5 as predictor (GZLM2) in the zebrafish 5-week chronic unpredictable stress (CUS) model used in the present study.

Endpoints	Df	χ^2^	p	Groups compared	$\mathrm{\Delta M}$	95% confidence interval	$\mathrm{z}.\mathrm{test}$	$\mathrm{p}$
**Novel tank test (NTT)**								
Distance, cm	2.00	13.22	< 0.01	Control–fluoxetine	− 28.33	[− 269.06, 212.41]	− 0.23	NS
				Control–stress	− 400.19	[− 640.93, − 159.45]	− 3.26	0.001
				Fluoxetine–stress	− 371.86	[− 612.60, − 131.13]	− 3.03	0.002
Time spent not moving. s	2.00	2.95	NS					
Time spent top, s	2.00	34.81	< 0.01	Control–fluoxetine	41.10	[− 6.39, 88.59]	1.70	NS
				Control–stress	139.13	[91.64, 186.61]	5.74	< 0.001
				Fluoxetine–stress	98.03	[50.54, 145.51]	4.05	< 0.001
Number of top entries	2.00	2.74	NS					
Latency to enter top, s	2.00	17.99	< 0.01	Control–fluoxetine	− 31.44	[− 94.75, 31.88]	− 0.97	NS
				Control–stress	− 131.20	[− 194.52, − 67.89]	− 4.06	< 0.001
				Fluoxetine–stress	− 99.77	[− 163.08, − 36.45]	− 3.09	0.002
**Light–dark test (LDT)**								
Time spent in light, s	2.00	0.41	NS					
Number of light entries	2.00	0.53	NS					
**Shoaling test (ST)**								
Inter-fish distance, cm	2.00	10.02	0.01	Control–fluoxetine	0.09	[− 0.17, 0.34]	0.67	NS
				Control–stress	0.39	[0.14, 0.64]	3.01	0.003
				Fluoxetine–stress	0.30	[0.05, 0.56]	2.35	0.019
Distance to surface, cm	2.00	3.39	NS					
**Zebrafish tail immobilization test (ZTI)**								
Time spent active, s	2.00	0.40	NS					
**Neurochemical endpoints**								
Dopamine, pg/mg	2.00	0.76	NS					
DOPAC/dopamine ratio	2.00	0.43	NS					
DOPAC, pg/mg	2.00	1.75	NS					
5-HIAA, pg/mg	2.00	17.69	< 0.01	Control–fluoxetine	0.84	[0.37, 1.30]	3.52	< .0001
				Control–stress	− 0.05	[− 0.52, 0.41]	− 0.23	NS
				Fluoxetine–stress	− 0.89	[− 1.35, − 0.42]	− 3.75	< 0.001
5-HIAA/serotonin ratio	2.00	10.82	< 0.01	Control–fluoxetine	0.89	[0.30, 1.47]	2.98	0.003
				Control–stress	0.27	[− 0.38, 0.93]	0.82	NS
				Fluoxetine–stress	− 0.62	[− 1.15, − 0.08]	− 2.27	0.023
Serotonin, pg/mg	2.00	3.09	NS					
HVA/dopamine ratio	2.00	3.58	NS					
HVA, pg/mg	2.00	3.21	NS					
Norepinephrine, pg/mg	2.00	1.05	NS					

Behavioral endpoints were tested in the novel tank test (NTT), light–dark (LDT), shoaling (ST) and zebrafish tail immobilization (ZTI) tests, also see Figs. 2, 3, Table 2 and Supplementary Tables S1–S5 for details.

*NS* no significant differences (p > 0.05), *DOPAC* 3,4-dihydroxyphenylacetic acid, *5-HIAA* 5-hydroxyindoleacetic acid, *HVA* homovanillic acid, *Df* degree of freedom.

Applying GZLM1 analyses to the light–dark test (LDT), showed significant group and week × group effects for time spent in light. Post-hoc Tukey’s test comparison of the weeks revealed significant differences between stress and control groups at weeks 1–3, but not other weeks of CUS (Table 2, Supplementary Tables S2–S4). In contrast, there were no significant effects for any predictor studied for the number of light entries (Table 2), and no group effects were observed in GZLM2 using the Wald test for the LDT endpoints (Table 3).

In the shoaling test (ST), there was a significant group effect, but not week or interaction, for the average inter-fish distance. The distance to the surface showed significant week and week × group, but no group effects (Table 2), with groups differing at weeks 1–2, but not other CUS weeks (Supplementary Table S5). GZLM2 analyses also showed significant group effects, with stress vs. control and vs. fluoxetine fish for the average inter-fish distance, as assessed by Tukey’s post-hoc testing (Table 3).

Finally, GZLM1 analyses of the zebrafish tail immobilization (ZTI) test data established significant week and week × group (but not group) effects for time spent active (Table 2), with no significant GZLM2 effects using the Wald test for the ZTI test endpoints (Table 3). There was also a significant week × group interaction for the 5-hydroxyindoleacetic acid (5-HIAA) content and the 5-HIAA/serotonin ratio, with no week or group effects (Table 2). Furthermore, significant stress vs. control week differences were found for weeks 2–4 (Fig. 3, Supplementary Table S5). Finally, GZLM2 analyses revealed significant group effects for 5-HIAA levels, with significant fluoxetine vs. control and vs. stress differences for post-hoc Tukey’s test (Table 3). GZLM2 also revealed altered 5-HIAA/serotonin ratios, with fluoxetine significantly differing vs. control and stress, as assessed by the Tukey’s test pairwise comparisons (Table 3).

The Gamma-test revealed 13 significant correlations (Table 4), including cross-test correlations (e.g., NTT distance and ST inter-fish distance, r =  − 0.55, p < 0.05) and correlations between behavioral and neurochemical endpoints (e.g., the ZTI time spent active and the 3,4-dihydroxyphenylacetic acid (DOPAC)/dopamine ratio, r = 0.48, p < 0.05). As already mentioned, no effect as a predictor in GZML1 analyses found no behavioral battery exposure impact on neurochemistry (Supplementary Table S6). Because no differences were found for neurochemical endpoints between CUS sub-groups of behaviorally tested vs. naive untested fish, combining these two sub-groups into one cohort for analyses was justifiable (see Supplementary Table S6 for statistical details).

**Table 4:** Correlations matrix of group × week subgroups for behavioral and neurochemical endpoints assessed by the Goodman and Kruskal’s gamma correlation test.

Endpoints	2	3	4	5	6	7	8	9	10	11	12	13	14	15	16
1. Time not moving, s	** − 0.6**	0.2	0.3	0.3	** − **0.3	0.1	0.2	0.0	** − **0.2	0.2	0.2	0.2	** − **0.3	** − **0.2	0.0
2. Distance traveled, cm		** − **0.4	** − 0.6**	** − 0.5**	0.2	0.0	0.0	0.0	0.0	0.1	** − **0.2	0.1	0.1	0.1	** − **0.1
3. Time spent top, s			0.4	0.4	** − **0.3	** − **0.4	** − **0.2	** − **0.2	0.2	** − **0.3	0.1	** − **0.2	0.2	** − **0.3	0.3
4. Time in light, s				0.1	** − **0.1	** − **0.1	** − **0.4	** − **0.1	0.0	** − 0.5**	0.0	-0.1	0.0	** − **0.1	0.2
5. Inter-fish distance, cm					** − **0.3	** − **0.2	0.0	0.0	0.0	0.0	0.2	** − **0.2	0.1	** − **0.1	0.1
6. Distance to surface, cm						0.3	0.0	0.2	0.3	0.2	0.3	0.2	0.2	0.4	0.2
7. Time active, cm							0.1	0.2	0.0	0.3	0.3	0.4	** − **0.1	**0.5**	0.1
8. Norepinephrine, pg/mg								0.3	0.0	**0.7**	0.1	0.4	** − **0.2	0.0	** − **0.3
9. DOPAC, pg/mg									0.3	**0.5**	0.3	**0.5**	0.1	**0.6**	0.1
10. 5-HIAA, pg/mg										0.2	0.4	0.2	**0.6**	0.1	0.2
11. Dopamine, pg/mg											0.2	**0.6**	** − **0.1	0.1	** − **0.2
12. HVA, pg/mg												0.2	0.4	0.4	**0.6**
13. Serotonin, pg/mg													** − **0.1	0.1	** − **0.2
14. 5-HIAA/serotonin ratio														0.2	**0.5**
15. DOPAC/dopamine ratio															0.3
16. HVA/dopamine ratio															1

Statistically significant correlations are bolded (p < 0.05).

*DOPAC* 3,4-dihydroxyphenylacetic acid, *5-HIAA* 5-hydroxyindoleacetic acid, *HVA* homovanillic acid.

**Figure 4:**
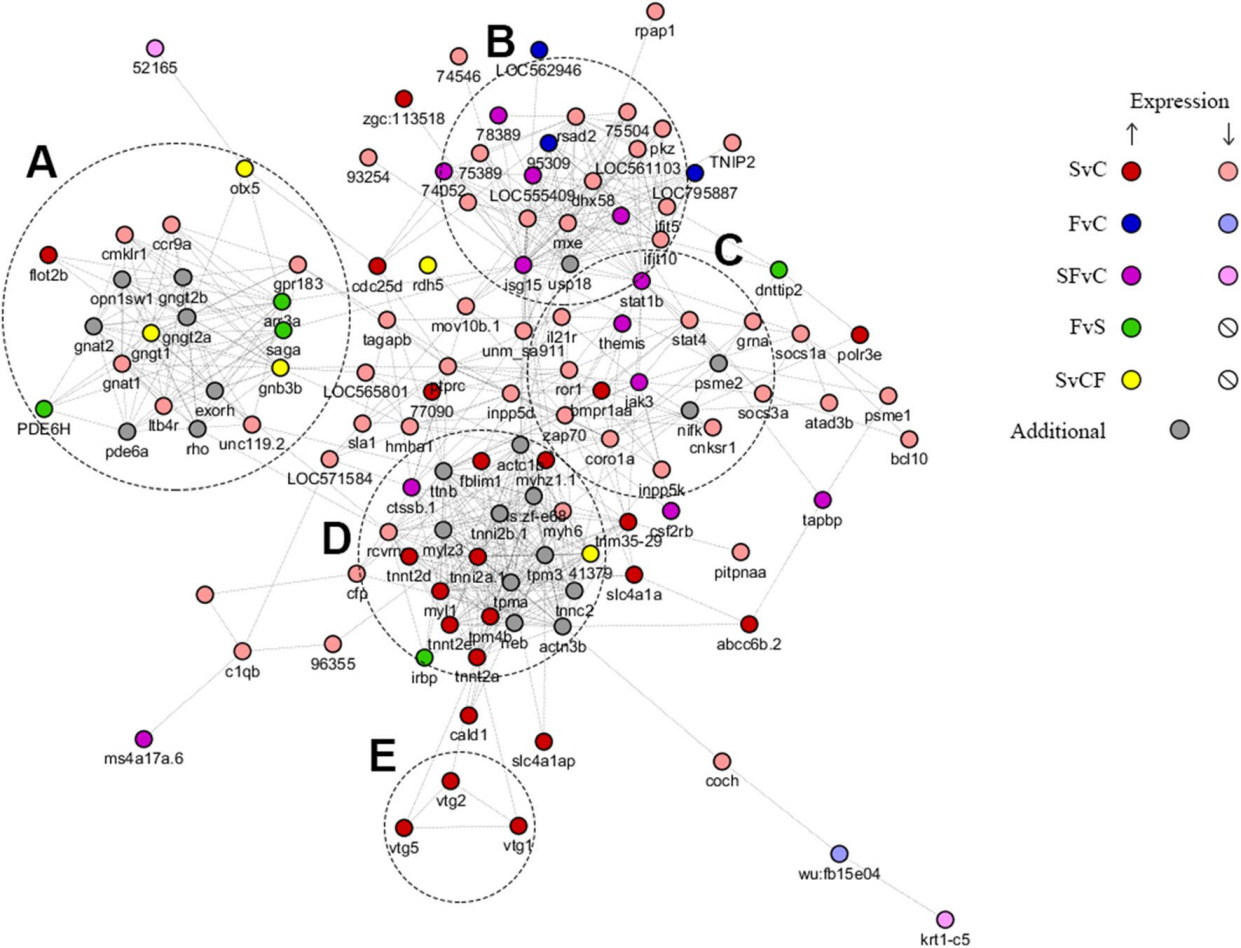
The network of protein–protein interactions (PPI) constructed for differentially expressed genes (found in all analyses) using the STRING online database^151^ (see “Methods” section and Supplementary Figs. S1–S3 for details). Genes represented as numbers refer to “ENSDARG000000*”, where * denotes the last 5 digits of the Ensembl gene names (ID). The network was visualized using the CytoScape application^149,150^. *SvC* stress vs. control, *FvC* fluoxetine vs. stress, *SFvC* differentially expressed in both SvC and FvC (in same l2fc direction), *FvS* fluoxetine vs. stress, *SvCF* differentially expressed in SvC and FvS (in opposite directions, color refers to the direction of FvS expression change), Additional—20 proteins with the highest interaction score in STRING for suggested PPI networks. Letters denote several clusters of genes, including (**A**) arrestins and G protein-coupled receptors (GPCRs) related genes, (**B**) ubiquitin-related genes and their inflammatory modulators, (**C**) inflammation-related transcription factors, (**D**) cytoskeletal and motility related proteins, (**E**) vitellogenins.

**Figure 5:**
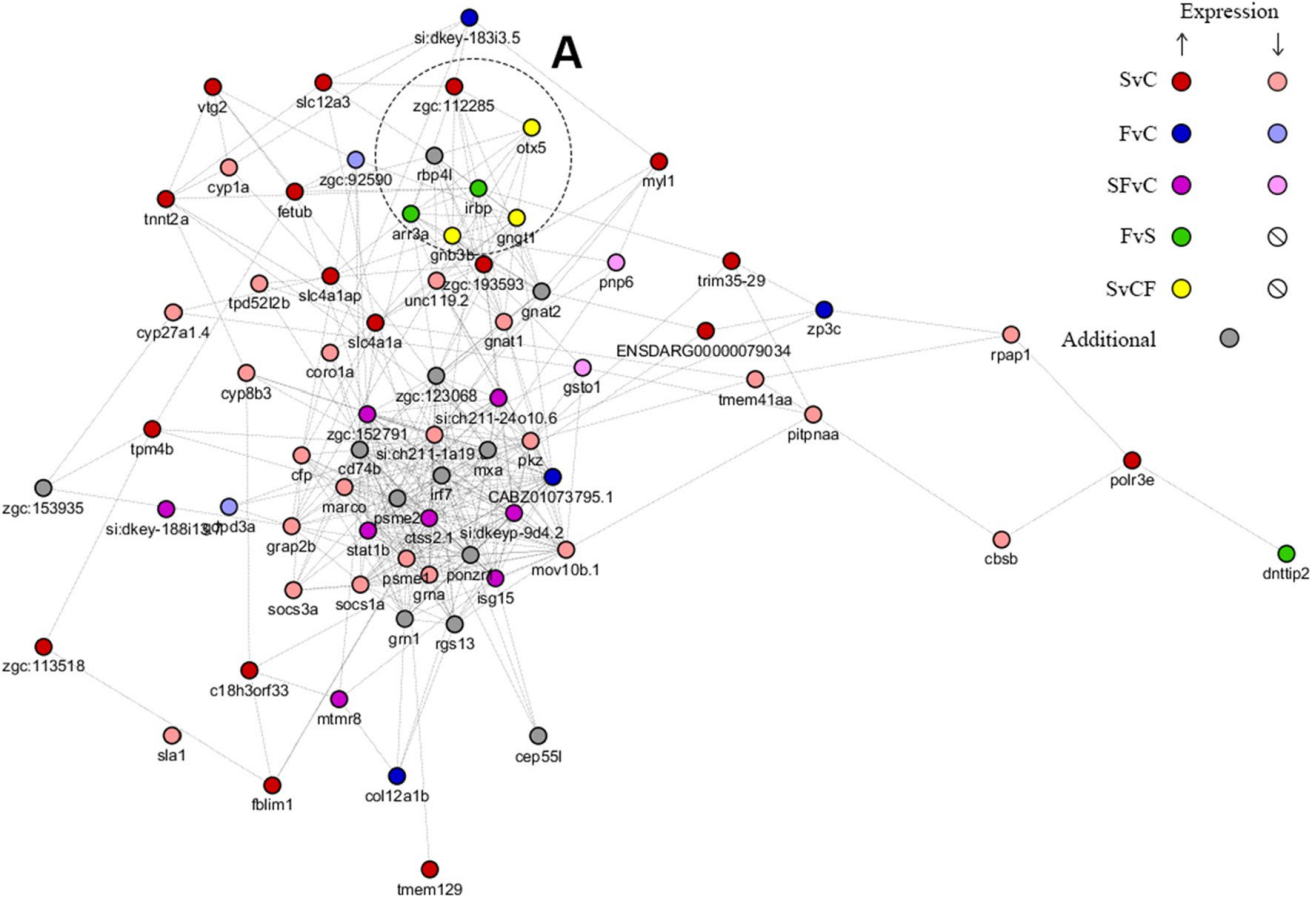
Gene co-expression (GE) network constructed for differentially expressed genes (found in all analyses) using the GeneMANIA^155–158^ online database (see the “Methods” section, and Supplementary Figs. S1–S3 for details of treatment and network construction). The network was graphically presented using the CytoScape application^149,150^. *SvC* stress vs. control, *FvC* fluoxetine vs. stress, *SFvC* differentially expressed in both SvC and FvC (in same l2fc direction), *FvS* fluoxetine vs. stress, *SvCF* differentially expressed in both SvC and FvS (in the opposite direction, color refers to the direction of FvS expression change), Additional—20 genes with the highest co-expression score in GeneMANIA for suggested PPI networks. (**A**) fluoxetine vs. stress-related genes that are commonly co-expressed.

Our CNS genomic analyses revealed a total of 196 genes (130 downregulated and 66 upregulated) differentially expressed in stressed vs. control fish (Figs. 4, 5, Supplementary Table S8, Figs. S1–S5), as well as 49 genes (34 downregulated and 15 upregulated) differentially expressed in fluoxetine vs. control groups. Overall, 35 genes (25 downregulated and 10 upregulated) were differentially co-expressed in the same direction in the stressed and fluoxetine (vs. control) groups, likely representing CUS-related genes resistant to antidepressant treatment (Figs. 4, 5, Supplementary Table S8, Figs. S1–S5). Finally, differential expression in fluoxetine vs. control fish involved 18 genes (3 downregulated and 15 upregulated), among which 9 (2 downregulated and 7 upregulated) genes have already been noted as differentially expressed (but in the opposite direction) in stress vs. control fish, thus likely representing a set of genes whose expression was altered by CUS but became normalized/rescued by the antidepressant treatment.

The Generally Applicable Gene-set Enrichment (GAGE, see the “Methods” section for details) of these results (Supplementary Table S9) revealed 87 Gene Ontology (GO) and 10 Kyoto Encyclopedia of Genes and Genomes (KEGG)-listed pathways differentially expressed in stressed vs. control fish, forming 13 essential GO and 8 KEGG gene sets. For fluoxetine-treated vs. control fish, GSEA yielded 68 GO pathways and 6 KEGG pathways, clustered into 9 GO and 5 KEGG essential pathways. Fluoxetine vs. stressed fish GAGE analysis revealed 19 GO and 3 KEGG pathways, organized in 7 and 3 essential sets respectively. Finally, topological analyses (focusing on potential importance of proteins and genes as therapeutic targets) of both these genes and their protein products linked some hub nodes, such as *tpm4b*, *isg15*, *mov10b.1*, *CABZ01073795.1*, *mxa*, to both zebrafish CUS and its treatment (Fig. 4, Table 5 and Supplementary Fig. S4, S5).

**Table 5:** Top 10 nodes analyzed using the double screening scheme (DSS) analysis, combining the density of maximum neighborhood component (DMNC) and maximum neighborhood component (MNC, see “Methods” section for details; DMNC||MNC), degree, or bottleneck methods, for networks of constructed protein–protein interactions (PPI; STRING database) and gene co-expression (GE; GeneMANIA; see “Methods” section and Fig. 5 for details).

Genes or Proteins	DMNC	MNC	Genes or Proteins	Degree	Genes or Proteins	Bottle-neck
**STRING (PPI)**						
tpm4b	0.81	23	isg15	32	isg15	31
tnni2b.1	0.96	20	ttnb	29	actc1b	24
myh6	0.87	20	actc1b	28	Ttnb	22
tnnc2	0.84	20	stat1b	26	saga	11
tnni2a.1	1.00	19	tpma	25	trim35-29	10
mylz3	0.88	19	tpm3	24	stat1b	9
ns:zf-e68	0.83	18	tpm4b	23	ENSDARG00000004953	9
neb	0.96	17	actn3b	23	myhz1.1	9
tnnt2a	0.98	16	myl1	21	ptprc	7
tnnt2d	0.94	15	usp18	21	arr3a	7
**GeneMANIA (GE)**						
*mov10b.1*	0.82	24	*CABZ01073795.1*	36	*mxa*	13
*cd74b*	0.78	24	*mxa*	28	*CABZ01073795.1*	11
*stat1b*	0.77	24	*isg15*	25	*si:ch211-1a19.3*	10
*si:dkeyp-9d4.2*	0.75	23	*stat1b*	25	*slc4a1a*	10
*ctss2.1*	0.73	23	*si:ch211-1a19.3*	25	*irbp*	7
*isg15*	0.91	22	*irf7*	24	*cd74b*	6
*ponzr1*	0.90	22	*psme2*	24	*irf7*	5
*psme2*	0.86	22	*mov10b.1*	24	*ctss2.1*	5
*zgc:152791*	0.75	22	*cd74b*	24	*si:ch211-24o10.6*	5
*irf7*	0.91	21	*si:dkeyp-9d4.2*	24	*grap2b*	5

**Figure 6:**
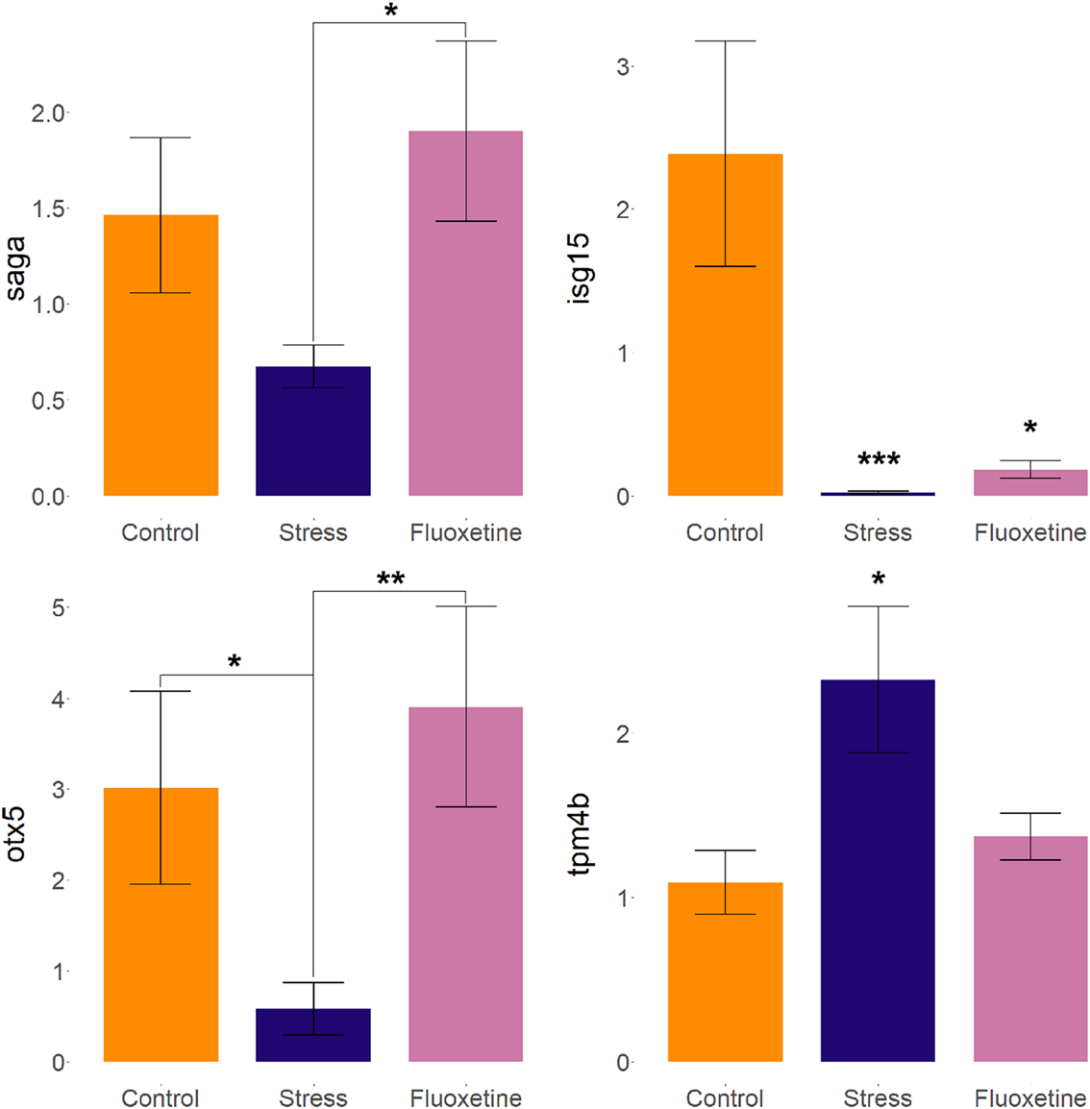
Qualitative real-time polymerase chain reaction results of the last (fifth) week of CUS showing differential expression of selected four reference genes in adult zebrafish brain samples with significant expression differences detected previously by the RNA-seq. Data is analyzed and represented using the Pfaffl method. *p < 0.05, **p < 0.01, ***p < 0.001 vs. controls or vs. the group connected by a horizontal line, post-hoc Dunn’s test for pairwise comparisons for significant Kruskal–Wallis data (see Supplementary Table S10 for details). Graphs were constructed using the ggplot2 R package^137^.

As shown in Fig. 6, the qualitative real-time polymerase chain reaction (qRT-PCR) analyses of the same samples successfully validated the RNA sequencing results reported above (see also Supplementary Table S10 for statistical data). Specifically, among several reference genes we selected for such validation, *isg15* significantly reduced expression in both stress and fluoxetine groups vs. control, the *tpm4b* expression increased in stressed vs. control groups, *saga* expression decreased in stressed vs. fluoxetine group, and *otx5* in stressed vs. both control and fluoxetine groups (Fig. 6, Supplementary Table S10). The genes for qRT-PCR analyses were chosen based on their hubness in core PPI groups and on different expression patterns between groups (see the “Methods” section for details).

## Discussion

The present report is the first large-scale study that explored in-depth complex weekly behavioral and neurochemical dynamics of CUS-evoked states in adult zebrafish, testing a wide range of anxiety-, social and despair-like behaviors. We also paralleled these data with a genome-wide brain transcriptomic screening of the 5-week CUS effects, and performed in-silico modeling of molecular networks associated with the identified differentially expressed genes.

Our behavioral findings can be briefly summarized as follows: first, overt NTT anxiety (reduced top exploration) was the most stable behavioral effect observed weekly following the CUS protocol here (Fig. 2, Tables 2, 3). In contrast, the LDT behavior was insensitive to anxiety-like changes at weeks 4 and 5, corroborating a putative lesser sensitivity of this test (than NTT) to anxiety^49,50^. Moreover, the ZTI activity decreased at week 1, increased at week 3, and remained unaltered at other CUS weeks (Fig. 2, Table 2), suggesting rather complex interactions between anxiety- and despair-like phenotypes in zebrafish (also see complex CUS effects on despair in rodent models^51–54^). Finally, shoals of the stressed fish displayed shorter inter-fish distance, consistent with increased NTT anxiety-like behavior in fish observed here (Fig. 2, Table 2) and reported previously for zebrafish stress models in the literature^40,47,50,55,56^.

Chronic fluoxetine reversed most of the CUS-evoked behavioral phenotypes in the present study (Fig. 2), thus resembling anti-stress anxiolytic/antidepressant effects observed clinically (Table 3). Notably, there were overt fluctuations in baseline behaviors in control fish over time (Fig. 2), likely due to some environmental influences beyond of experimenter’s control (e.g., atmospheric pressure) that would affect all study groups equally. However, this observation parallels daily variance of zebrafish behavior reported earlier^57^, hence corroborating the overall behavioral validity of the present study.

Summarized in Fig. 3 and Tables 2 and 3, neurochemical analyses strongly implicate the serotonergic system in zebrafish CUS, consistent with similar serotonergic responses in rodent CUS^58–60^ and human affective disorders^61–64^, and suggesting shared, evolutionarily conserved serotonergic mechanisms underlying CUS. Indeed, we found reduced levels of a serotonin metabolite 5-HIAA and the 5-HIAA/serotonin ratios, as well as their increase at week 2 of stress. The 5-HIAA/serotonin ratio is common biomarker of CNS serotonergic activity, reflecting the activation of serotonergic neurons and their serotonin release, followed by its metabolization^65,66^. Interestingly, our findings also parallel reduced 5-HIAA in 15-day zebrafish CUS reported in a different study earlier^67^. Thus, altered 5-HIAA levels and the 5-HIAA/serotonin ratios only emerged from week 2 in the present study—an important methodological observation suggesting that shorter (e.g., 1-week) protocols based on CUS exposure may be insufficient for (or less capable of) evoking pathological alterations in the serotonergic system of zebrafish.

To further integrate behavioral and neurochemical CUS-evoked alterations in zebrafish, we performed correlational analyses of behavioral and neurochemical endpoints, comparing all average values for each endpoint at each individual week of CUS (Table 4). While brain dopamine levels negatively correlated with the LDT time in light, the DOPAC/dopamine ratio positively correlated with the ZTI time spent active (Table 4), supporting the link between the DOPAC/dopamine ratio and zebrafish despair-like behavior, already noted in this test^68^. NTT distance negatively correlated with NTT time not moving, LDT time in light and the ST average inter-fish distance (Table 4). However, using group means values (instead of individual fish values) could somewhat limit such analyses, thus representing only pilot correlational findings. Moreover, like genomic assays, neurochemical analyses performed here involved whole-brain samples (rather than individual brain regions), likely representing a less sensitive approach for probing potential region-specific changes in neurochemistry and genomics, therefore necessitating further studies of region-specific changes during CUS in zebrafish.

Furthermore, multiple zebrafish CUS studies have shown increased anxiety following a 2-week (or longer) CUS protocols^47,48,56,67^, consistent with the present findings (Fig. 2, Tables 2, 3). However, some other reports failed to evoke overt anxiety^69^, raising questions of whether CUS protocol was indeed properly applied in such studies (e.g., see^70,71^ for a recent discussion of challenges with data reliability and replicability in zebrafish behavioral models). Thus, further research is needed, for example, to compare zebrafish that received CUS protocols that would differ in the numbers of stressors, their severity and/or duration.

Nevertheless, zebrafish genomic data, analyzed here using different bioinformatic methods (see “Methods” section for details), revealed major genomic changes associated with stress exposure and its drug treatment (Figs. 4, 5, Table 5, Supplementary Tables S7, S8, and Supplementary Figs. S4, S5). Overall, stress induced overt CNS gene expression changes, differentially affecting genes involved in inflammation/cytokine-related signaling pathways, Mitogen-Activated Protein Kinase (MAPK) signaling and receptor tyrosine kinases, such as signal transducer and activator of transcription (*stat*) 1b and 4, interleukin 21 receptor (*il21r*), radical S-adenosyl methionine domain-containing protein 2 *(rsad2*), janus kinase 3 (*jak3*), zeta-chain-associated protein kinase 70 (*zap70*) and suppressor of cytokine signaling (*socs*) 1a and 3a, receptor tyrosine kinase-like orphan receptor 1 (*ror1*), thymocyte-expressed-molecule (*themis*) and altered pathways (GO:0004713 protein tyrosine kinase activity; dre04010 MAPK signaling pathway; Fig. 4 and 5, Supplementary Figs. S4, and S5). Similarly to previous studies, we also found changes in the expression of interferon inducible proteins IFI6/IFI27-like associated genes and pathways^69^ (Supplementary Fig. S5).

Furthermore, CUS upregulated various cytoskeletal and cell motility-related brain genes, such as encoding myosins (light chain 1 and heavy polypeptide 1.1 and 6) and troponins (T2a, T2d and 2a.1), also disturbing the expression of ubiquitin-related genes, such as interferon-stimulated gene 15 ubiquitin-like modifier (*isg15*), ubiquitin E3 ligases (*si:dkey-40c23.2*) and other associated genes (*zgc:163,136* ubiquitin-protein transferase; Figs. 4, 5, Supplementary Table S8, and Figs. S4, S5). Notably, these ubiquitin-related genes are linked to various interferon-associated genes, and *isg15* interacts and co-expresses with multiple other genes, thus representing the strongest hub gene found for CUS here (Fig. 5, Table 5). Stress also lowered the expression of *dre04744* Phototransduction (see further), and disturbed endocrine (especially steroid) function-related genes, increasing the vitellogenin *vtg1*, -*2* and *-5* genes expression (see further). Finally, CUS exposure affected the RNA processing-related pathways, including GO:0006397 mRNA processing, GO:0003735 structural constituent of ribosome, dre03010 ribosome, and dre03040 spliceosome and cell metabolism, GO:0009199 ribonucleoside triphosphate metabolism, dre01200 carbon metabolism, dre00010 glycolysis/gluconeogenesis, and dre00983 drug metabolism/other enzymes.

In contrast, fluoxetine normalized the expression of most of these CUS-affected genes, sharing with stress only 35 out of 196 ‘stress’ genes, mostly representing key inflammatory hubs associated with cytokine activity (e.g., *isg 15*, *stat1b*, *jak3*, Figs. 4, 5, Supplementary Table S8 and Fig. S1–S5). Similarly, while the expression of genes of some pathways was restored by fluoxetine, others remained differentially expressed (e.g., RNA-related pathways dre03010 Ribosome, dre03040 spliceosome, GO:0034660 ncRNA metabolic process, GO:0003735 structural constituent of ribosome and metabolic pathways, dre01200 carbon metabolism, GO:0009144 purine nucleoside triphosphate metabolism, GO:0019752 carboxylic acid metabolism, Supplementary Table S9). Furthermore, the GO:0006955 immune response expression decreased, thus potentially contributing to the overall reduction of activity of genes of cytokine-related pathways in the fluoxetine-treated fish.

To better understand fluoxetine effects on stress pathogenesis, we also compared the fluoxetine and CUS groups, revealing 18 differentially expressed genes, 9 of which were found in CUS vs. control fish, thus proving normalizing their expression by fluoxetine. Analyses of their PPI networks show that most of such differentially expressed genes are interrelated, and form a small functional sub-network that includes orthodenticle homolog 5 (*otx5*), arrestin 3a (*arr3a*), s-antigen (*saga*), and GPCR genes (*gngt1*, *gnb3b* and *gpr183a*).

In general, inflammation is an important factor in affective pathogenesis^72–76^. Complementing clinical data, animal inflammation-related models of affective disorders are widely used to recapitulate affective pathogenesis^24^. The 5-week CUS protocol used here has recently been shown to induce major alterations in both inflammatory and anti-inflammatory cytokine networks, all corrected by fluoxetine^48^. Similarly, the present CUS study revealed global brain expression changes in genes involved in inflammation, whereas fluoxetine treatment predictably normalized most of these genomic effects (Figs. 4, 5, Supplementary Table S8, and Figs. S1–S5).

The role of sex-related genes, especially estrogens, in the pathogenesis of depression is also thoroughly discussed in the literature^77–80^. In fish, vitellogenin is an important biomarker of endocrine disruption that is sensitive to estrogen exposure^81,82^. CNS expression of vitellogenin genes *vtg1*, *-2* and *-5* rose in the GO:0005179 ‘hormone activity’, GO:0000003 ‘reproduction’ and other related pathways, further implying endocrine deficits in stressed fish, and suggesting that changes in the expression of some endocrine system genes may contribute to affective pathogenesis. Moreover, CUS applied here strongly (l2fc = 8.4) increased the expression of adhesion GPCR G4b (*adgrg4b*), whereas fluoxetine restored it to control levels (Fig. 4, 5, Supplementary Table S8, and Figs. S4, S8). Interestingly, adhesion G protein–coupled receptors (aGPCRs) represent an important, but understudied family of proteins that participate in both cell adhesion and signaling functions^83^. Although the physiological roles of most of aGPCRs are poorly understood, some of them have already been linked to brain disorders^84^, since, for example, *Adgrb2* knockout mice display reduced depression-like behavior and enhanced hippocampal cell proliferation^85^.

Arrestins are a small family of highly homologous adaptor proteins that function as regulators of the GPCR signaling, supporting their internalization and activating independent (e.g., tyrosine kinase Src and MAPK-related) pathways^86–88^. The activity of arrestins in-vivo is important, given biased GPCR ligands that activate G protein- and arrestins-related pathways with different efficiency^89^. Arrestins have also been suggested as potential targets for treating affective pathologies^90^, since leukocytes of depressed patients show lower levels of beta-arrestin-1, whereas antidepressant treatment increases these levels in both rats^91^ and humans^92^. Furthermore, beta-arrestins may modulate central serotonergic system, including serotonin-induced activation of ERK1/2 by serotonin 5-HT_2A_ receptors, and since serotonin and 2,5-dimethoxy-4-iodoamphetamine (DOI) act through distinct (beta-arrestin-2-dependent and independent, respectively) mechanisms, to induce mouse head twitch responses^93^.

Notably, the biological function of arrestins depends on their ubiquitination and deubiquitination, which determine the efficiency of receptors internalization, the fate of the arrestin-receptor complex, and the signaling cascades it activates^94^. Similarly, ubiquitination also plays a role in antidepressant activity^95^, since citalopram, imipramine, desipramine and moclobemide all increase mRNA expression of beta-arrestin-2 in C6 rat glioma cells, but reduce its protein levels via rapid ubiquitinylation that promotes proteasomal degradation^95^. In line with this notion, we found altered expression of ubiquitin- and arrestins-related genes (e.g. *isg15*, dre04744 Phototransduction) in the CUS group, with a subtle increase in the expression of two arrestins- (*saga*, *arr3a)* and related pathways (dre04744 phototransduction, GO:0007601 visual perception expression) following fluoxetine treatment (Figs. 4, 5, Supplementary Tables S7, S8, and Figs. S4, S5). This further suggests the putative role of arrestins and their ubiquitination in affective pathogenesis and antidepressant treatment, with multiple potential translational implications, thus meriting further studies in other CNS stress models and other species.

In conclusion, our results show that CUS induced complex behavioral and neurochemical phenotypes in zebrafish that dynamically evolve over a 5-week battery. While some phenotypes, such as NTT anxiety, were relatively stable across time, other responses (e.g., ST shoaling and ZTI despair-like behaviors, as well as serotonin metabolism) may fluctuate weekly within the CUS battery. Finally, our genomic analyses revealed altered brain expression of multiple inflammation-related genes following CUS, partially rescues by chronic fluoxetine treatment. Collectively, these findings support zebrafish as a valuable translational tool to study stress-related pathologies. For example, while CUS exposure induced pronounced anxiety and serotonergic deficits in zebrafish (paralleling rodent and clinical studies), chronic fluoxetine exposure rescued most of these effects. Complementing these physiological findings, the present transcriptomic analyses further implicate neuroinflammation, structural neuronal remodeling and the arrestins/ubiquitin regulation, in both stress pathology and its mechanisms of treatment.

## Methods

### Animals and housing

Adult, 3–5 months old wild-type short-fin experimentally naïve zebrafish (approximately 50:50 male:female ratio) were obtained from a local distributor (Axolotl, Ltd., St. Petersburg, Russia) and housed for at least 3 weeks in standard conditions in groups of 10–15 fish in 4-L tanks (2.5–3.75 fish/L) at the Aquatic Facility of Almazov National Medical Research Center (St. Petersburg, Russia) in the ZebTec Active Blue Stands with Water Treatment Unit (Tecniplast, West Chester, USA), filled with filtered system water maintained at 27 ± 0.5 °C and pH 7.4. The illumination in the holding room (950–960 lx) was provided by 18-W fluorescent light tubes with a 12/12 light/dark cycle (unless specified otherwise in the CUS protocol, Table 1). All fish were fed twice a day with small food pellets Neon Micro Granules for fish size 1–2 cm long (Dajana Pet, Bohuňovice, Czech Republic) according to the zebrafish feeding standards^96^, unless specified otherwise in the CUS protocol detailed in Table 1. All fish belonged to the same baseline population and were allocated to the experimental groups randomly using a random number generator, and were acclimated at least 2 weeks before the experiments. As the animals were involved in the study, see Ethical Confirmation statement for approval and ethical details. All animals tested were included in final analyses, without removing outliers. All experiments were performed as planned, and all analyses and endpoints assessed were included without omission.

### Chronic unpredictable stress (CUS)

The study experimental design is summarized in Fig. 1 and Table 1, and utilized a 5-week CUS battery, as described previously^48^. The control fish were housed similarly to the experimental cohort, but remained experimentally naïve for the entire study duration, as in^48^. On Day 28, the stressed fish cohort was divided into two groups (Stress and Fluoxetine) that both continued to receive stressors (Table 1), with the latter group also receiving chronic fluoxetine (0.1 mg/L) during the last 11 days of the study. Fluoxetine was selected here as a classical SSRI antidepressant with well-established clinical activity^97–99^ and proven efficacy in both rodent^100–103^ and zebrafish models^40,46,48,104,105^. The dose and the treatment duration for the drug were selected based on our previous zebrafish CUS studies^48^.

### Behavioral testing

Behavioral testing of parallel zebrafish cohorts with varying stress durations (from 1- to 5-week CUS) was performed weekly within a 3-day test battery one day after the last CUS stressor was applied (Fig. 1, Table 1). Behavioral analyses were performed between 9:00 am and 6.00 pm (NTT, ST and ZTI between 9:00–12:00 am, LDT between 3:00–6:00 pm) by individually exposing zebrafish to a standard behavioral battery consisting of the NTT (Day 1 morning), LDT (Day 1 afternoon), ST (Day 2 morning) and the ZTI test (Day 3 morning), as shown in Fig. 1. Testing zebrafish one day after the last stressor application was chosen here in order to avoid potential confounding ‘immediate’ effects of acute stressors, thus focusing instead on baseline persistent effects of chronic stress per se. Fish that underwent behavioral testing were excluded from CUS protocol on those respective CUS days, did not receive stressors during the testing and were euthanized after the last behavioral test (ZTI). The 5-week CUS battery was chosen here as an established model in our laboratory, efficiently inducing pronounced behavioral and molecular changes in zebrafish^48^ consistent with other CUS procedures. Prior to behavioral testing, all fish (n = 20 at weeks 1–3, and n = 15 at weeks 4–5, Fig. 1) were transported from the holding room and acclimated to the testing room for 2 h. After behavioral testing, fish were returned to their respective hometanks and placed back to the aquatic housing system. The specific test battery used was chosen here because although zebrafish generally display a relatively weak sensitivity to the test battery effect^106^, the battery was organized in the order of stress intensity, i.e., from lower- to higher-stress, aiming to reduce any potential prior test experience effects, as suggested in^32^.

The NTT was chosen here as the most sensitive and commonly used behavioral test to assess anxiety and locomotion in zebrafish^49,107^, performed similarly to^108^. The NTT apparatus consisted of a 2-L acrylic rectangular tank (20 height × 20 length × 5 width cm) filled with water up to 19 cm, and divided into two equal virtual horizontal portions. Back and lateral sides of the tank were covered with nontransparent white covers (fixed to the outside walls), to increase contrast and reduce external visual clues during behavioral recording. Trials were video-recorded using an SJ4000 action camera (SJCAM, Ltd., Shenzhen, China) at 60 frames/s. We assessed the mean distance (cm), not moving duration (s) and time spent in top (s), based on the center body position computation, using the EthoVision XT11.5 software (Noldus IT, Wageningen, Netherlands), as in^57,109^.

The LDT was chosen here as another widely used test to study anxiety in zebrafish^49,110^. The apparatus represented a 20-L acrylic tank (20 height × 50 length × 20 width cm) divided into two equal compartments (one white and one black) filled with water for up to 15 cm. Trials were recorded by an SJ4000 video-camera and stored for further off-line analyses, similar to the NTT testing. Each video was then scored offline by highly trained observers (blinded to the treatments) to assess time spent (s) in, and the number of entries to, the light (white) zone.

The ST was chosen for this study as a commonly used test to assess social and stress-related behavioral phenotypes in zebrafish^111^, using behavioral apparatus similar to the NTT described above. During testing, the fish from each group were placed in the tank in groups of 5 and (following a 5-min acclimation to the apparatus) their shoals were photographed using an SJ4000 video-camera every 10 s, resulting in 6 photos taken per tank (n = 24 for weeks 1–3, n = 18 for week 4–5), similar to^111,112^. Each photo was next calibrated to the size of the tank, and then analyzed by two highly-trained observers (blinded to the groups), measuring the average inter-fish distance (cm) and distance to the surface (cm) of each fish in the photo, using the ImageTool software (University of Texas Health Sciences Center, San Antonio, TX). The recorded NTT, LDT and ST behavioral endpoints fully corresponded to the established behavioral phenotypes described in the Zebrafish Behavioral Catalog (ZBC)^113^.

The ZTI test was applied here as a recently developed novel test for characterizing stress and drug effects in adult zebrafish, analogous to rodent despair-like behavioral tests, and bidirectionally sensitive to both acute stressors and antidepressant treatment^68^. Given complex interplay between despair-like behavior and chronic stress in rodents^28,114–116^, it was particularly interesting to assess the effects of CUS effects on zebrafish behavior in the ZTI test. Briefly, the caudal part of each fish was immobilized for 6 min using the wet viscose sponge (8 length × 4 height × 5 width cm) cut in the middle with a sharp scalpel to a 2-cm depth from the bottom, and attached to the top of the beaker using two additional 2-cm cuts of the sponge on the sides, to allow fixation by the beaker walls, as described in detail in^68^. The cranial part of the fish body thus remaining freely hanging vertically in a small beaker (a 7 × 5.5–4.8 cm transparent plastic cup shaped as a truncated cone) filled with water^68^. Trials were video-recorded using an SJ4000 action camera at 60 frames/s, and then scored offline by three highly trained observers to assess the total duration (s) of active escape attempts (defined here as continuous bouts of body torsion movements separated from each other by episodes of immobility—complete cessation of body movements, except for gills and eyes) for more than 2 s each, according to^68^.

### Neurochemical analyses

Brain monoamines are an important factor in stress and affective disorders both clinically and in rodents and zebrafish models^37,68,117–121^. To study dynamic changes in their levels in stressed zebrafish, the whole-brain concentrations of norepinephrine (NE), serotonin (5-HT), dopamine (DA) and their metabolites 5-HIAA, DOPAC and homovanillic acid (HVA) were assayed using the high-performance liquid chromatography (HPLC), as in^68,119–121^. As shown in Fig. 1, brain samples were collected one day after the behavioral battery, between 9:00 and 13.00 h. The 1-day interval was used here to minimize concomitant immediate effects of behavioral testing and/or handling, enabling us to focus on baseline CUS-evoked neurochemical changes instead. In our pilot studies, we used behaviorally tested (n = 5 for group × week pair, i.e., exposed to the battery of tests) and experimentally naïve (n = 5, unexposed to behavioral testing) fish, to analyze their neurochemical parameters. As we found no significant effect of tested vs. naïve CUS-exposed fish as predictor in the Generalized Linear Model (GZLM, Supplementary Table S7), the two fish subgroups were combined for assessing neurochemical changes. Fish for neurochemical analyses were chosen randomly from the respective experimental and testing groups, using an online random number generator (www.random.org). Samples were used for further analyses without pooling.

Briefly, the fish were euthanized in ice-cold water immediately after being taken from the hometanks, and their brains dissected on ice and stored in liquid nitrogen for prior analyses, as in^68^. On the day of analyses, all samples were weighted and placed into 10 μL of ice-cold 0.1-M perchloric acid (Sigma Aldrich, St. Louis, MO, USA) solution with 100 ng/mL 3,4-dihydroxybenzylamin (DHBA, internal standard) per 1 mg of brain tissue for the preservation of neurochemical analytes, similar to^68^. Then samples were next sonicated for 10 s at half-power settings, cleared by centrifugation and filtered through a 0.22-μm Durapore-PVDF centrifuge filter (Merck Millipore, Billerica, MA, USA), as in^68^. HPLC was performed using a CA-5ODS column and with a HTEC-500 chromatograph (Eicom, San Diego, CA, USA) with a carbon WE-3 G electrode WE-3 G using a + 650-mV applied potential. Chromatography mobile phase consisted of 0.1 M phosphate buffer, 400 mg/L sodium octylsulphonate, 50 mg/L ethylenediaminetetraacetic acid (EDTA), 17—% methanol and was adjusted to pH 4.5 by phosphoric acid (all reagents were purchased from Sigma Aldrich, St. Louis, MO, USA), as in^68^. The concentrations data were normalized using individual DHBA sample concentrations, and presented as pg/mg of brain tissue weight. We also assessed the 5-HIAA/serotonin, DOPAC/dopamine and HVA/dopamine ratios, reflecting the turnover of the respective monoamines in the brain, similar to^68^.

### RNA-sequencing

Brain samples for gene expression analyses were collected without pooling (1 brain per sample) one day after the last test of behavioral battery, between 9:00 and 13.00. The 1-day interval was used here to minimize concomitant immediate genomic effects of behavioral testing and/or handling, enabling us to focus on baseline CUS-evoked changes instead. Fish (n = 6–7) for RNA-sequencing analysis were chosen randomly from the experimental groups using a random number generator. Similar to the neurochemical analyses, the fish were quickly euthanized in ice-cold water immediately after being taken from the hometanks, and their brains dissected on ice and stored in liquid nitrogen for further analyses. For RNA isolation, brains were frozen in liquid nitrogen immediately after dissection. RNA isolation was made with TRI-reagent (MRC, Cat. no. 118) according to manufacturer instructions. Quality was checked with Quantus, electrophoresis, and QIAxel. PolyA RNA was purified with Dynabeads mRNA Purification Kit (Ambion). Illumina library was made from polyA RNA with NEBNext Ultra II Directional RNA Library Prep Kit for Illumina (NEB) according to manual. Sequencing was performed on Illumina HiSeq2500 with 140 bp read length, with at least 27 million reads generated for each sample.

### Qualitative real-time polymerase chain reaction (qRT-PCR)

To reconfirm the validity of our RNAseq data, we also conducted a small qualitative real-time PCR study of selected genes, quantifying whole-brain expression of four ‘reference’ genes (*isg15, saga, otx5 and tpm4b*) and one housekeeping gene (*b-act*) using the established qrt-PCR protocol^48,119,122,123^, with minor modifications. The genes were chosen based on their hubness in core PPI groups and on different expression patterns between groups (*isg15*—differentially expressed in both stress and fluoxetine vs. control, *saga*—in stress vs. fluoxetine, *otx5*—stress vs. both control and fluoxetine and *tpm4b*—in stress vs. control). Primers were designed using the National Center for Biotechnology Information (NCBI) Primer-BLAST (Basic Local Alignment Search Tool) database (https://blast.ncbi.nlm.nih.gov/Blast.cgi), or using primers from our past experiments (*b-act*) (Supplementary Table S1) and synthesized by Evrogen, Ltd. (Moscow, Russia). Similar to other molecular analyses here, samples for qRT-PCR were collected without pooling (1 brain per sample) one day after the last test of behavioral battery, between 9:00 am and 1.00 pm. As already noted, the 1-day interval was used here to minimize concomitant immediate genomic effects of behavioral testing and/or handling, enabling us to focus on baseline CUS-evoked changes instead. Fish (n = 10) for analysis were chosen randomly from the experimental groups using an online random number generator. The fish were quickly euthanized in ice-cold water immediately after being taken from the hometanks, and their brains dissected on ice and stored in liquid nitrogen for further analyses. RNA isolation was made with TRIzol analogue ExtractRNA (Evrogen, Ltd.) according to manufacturer instructions. We next synthesized cDNA using oligo(dT)20 primers using similar amount of RNA per sample (MMLV RT kit by Evrogen, Ltd.). Finally, we performed qRT-PCR with qPCRmix-HS SYBR (Evrogen, Ltd.) using CFX Connect Real-Time system (Bio-Rad laboratories, Hercules, CA, USA) in 3 replicates for each sample. The PCR sequences consisted of an initial incubation for 5 min at 95 °C to activate the Taq DNA polymerase, followed by 95 °C for 20 s (denaturing), 60 °C for 30 s (annealing), and 72 °C for 20 s (extension). Gene expression levels were normalized to the RNA expression of the housekeeping β-actin gene (relative quantification) using the Pfaffl method^124,125^. All samples with detectable signals were included in final analyses reported here.

### Statistical analyses and data handling

The present study utilized GZLMs to analyze dynamic changes observed following chronic unpredictable stress and fluoxetine treatment. GZLM is a generalization of regression methods that allows variables to have distributions other than a normal distribution, thus making it suitable for non-normal data analyses^126^. GZLM are widely used in various fields^127–129^, including zebrafish neurobehavioral studies^130^. For behavioral and neurochemical analysis, we performed the Wald chi-square (χ^2^) analysis of variance (ANOVA, Type II; Tables 2, 3) for GZLM (Supplementary Tables S1, S5), fits, followed by Tukey’s post-hoc testing for significant GZLM/Wald pair-wise comparison data (Figs. 2, 3, Tables 2, 3, Supplementary Tables S2–S4). GZLM is an effective method for analyzing multifactorial data that provides robust results both for nonparametric and parametric data^127,128,131,132^. To assess dynamic effects on zebrafish behavior, GZLM week, group and their interaction effects were used as predictors comparing stress and control group at CUS weeks 1–5 (GZLM1). To study fluoxetine treatment effects, we used another model with only 3 groups (control, stress, fluoxetine) at week 5 of treatment, to avoid dynamic model unsaturation (lack of weeks 1–4’s effect for fluoxetine group), thus limiting ANOVA capabilities to assess drug effects (GZLM2). To choose optimal GZLM distribution and link functions (goodness of fit) for each endpoint, we compared (where applicable) the Akaike information criterion (AIC) levels^133,134^ of Gaussian distribution (identity link), Poisson distribution (with log link), Gamma distribution (inverse and log links) and Inverse Gaussian distribution (with inverse and log links), choosing the least AIC score (indicating the model most likely to be correct)^135^. To assess potential behavioral battery effects on zebrafish neurochemistry, we applied a similar approach, using GZLM with week, group, their interaction and battery in CUS weeks 1–5 control vs. stress effects. Since no effect of behavioral battery was observed in any given model (p > 0.05 by GZLM, Supplementary Table S7), the test battery factor was excluded from further analyses, thus modeling only group, week and their interaction as predictors. To analyze week 5 data, we used a similar GZLM approach. All calculations were performed using the R software^136^.

The Goodman and Kruskal’s gamma correlation test (gamma-test) was used to study correlations between all mean values of endpoints observed in the groups for each week (mean values of group × week pairs). Since no fish was individually traced during behavioral testing and subsequent neurochemical analysis, mean values of endpoints observed in the groups for each week were used for the gamma test, resulting in n = 12 samples. Additionally, we studied neurochemical effects of behavioral battery comparing fish exposed to the battery (n = 5) with fish that continued to be exposed to the CUS protocol without battery exposure, using the Wilcoxon-Mann–Whitney U-test.

The sample size was chosen here based on previously published studies on zebrafish stress-related behavior, including own works^48,68,120,121,123^ and sample size estimation using the R package pwr2. Briefly, for GZLM1, the effect sizes for two factors were estimated using distance (as one of basic zebrafish endpoints) from^48^ (stress effect; effect size = 0.68) and^57^ (day effect; effect size = 0.75). Similarly, for GZLM2, the lowest distance effect size from^48^ among stress and fluoxetine was used (0.68). Power was chosen as 0.9 (0.1 chance of type II error), and alpha level was chosen as 0.05. The resulting n = 4 for GZLM1 and n = 11 for GZLM2 were further adjusted to account for potential mortality (e.g., due to CUS exposure), the number of individual fish samples needed for neuromolecular analyses, and potential presence of endpoints with smaller effect sizes. Finally, we initially started with n = 20 during the beginning of CUS protocol (weeks 1–3), but reduced n to 15 during weeks 4–5, in line with ethical principles to reduce the number of animals used in research, as no overt mortality was observed in the present study due to CUS. Graphs were constructed using the ggplot2 R package version 3.3.0 (https://ggplot2.tidyverse.org)^137^. All fish tested were included in final analyses without attrition or exclusion, and all planned analyses were reported here. All experimenters were blinded to the treatment groups during behavioral testing, neurochemical and genomic analyses, including statistics and video analysis using individual codes for fish/groups identification. Manual scoring of behavior was performed by two highly-trained experimenters blinded to the treatment (intra/inter-rater reliability > 0.85, as assessed by Spearman correlation).

To analyze differential gene expression, reads were mapped to the zebrafish GRCz11 reference genome using STAR spliced aligner^138^ and further processed using featureCounts^139^ to obtain raw gene counts. A total of 32,057 genes were used for analyses using the R environment for statistical computing^136^, Bioconductor software^140^, and DESeq2 package^141^. This method was chosen as a recommended tool for experiments with 12 or fewer replicates per condition, stable even within 0.5-fold-change thresholds, and generally consistent with other tools, such as EdgeR (when using exact test), Limma and EBSeq^142^. First, all rows without counts or only with a single count across all samples were removed from the analysis, yielding 28,932 genes. Differential expression analyses on the Negative Binomial (Gamma-Poisson) distribution were next performed by estimation of size factors, dispersion, and negative binomial generalized linear models and Wald statistics using the DESeq function. The p-values were adjusted using the Benjamini–Hochberg correction. p-value and false discovery rate (FDR) were set at 0.05. Differential expression analyses were applied to the stressed group vs. control group, fluoxetine treated group vs. control group and fluoxetine treated group vs. stressed group.

The resultant significantly altered genes in groups were verified for existence in other group comparisons for convenience, resulting in gene sets uniquely represented only in original differential expression analyses and gene sets shared between analyses, yielding in two sets: genes co-expressed in both stressed and fluoxetine groups vs. control and genes that were found to be differentially expressed in stressed group vs. control and then found to be differentially expressed between fluoxetine treated group vs. stressed group, thus restoring their expression levels to the control group levels. MA-plots (Bland–Altman plots) were constructed using the ggplot2 R package^137^. Unless specified otherwise, all data were expressed as mean ± standard error of mean (S.E.M.), and p set as < 0.05 in all analyses. For qRT-PCR analysis (Fig. 6), we performed Kruskal–Wallis (KW) test with post-hoc Dunn’s test for pairwise comparison for significant KW data (see Supplementary Table S10 for details). Analyses of all data in this study were performed offline without blinding the analysts to the treatments, since all animals and samples were included in analyses, data were analyzed in a fully unbiased automated method, and the analysts had no ability to influence the results of the experiments, as in^119^. The study experimental design and its description here, as well as data analyses and presenting, adhered to the ARRIVE guidelines for reporting animal research and the PREPARE guidelines for planning animal research and testing.

### Gene set enrichment analysis (GSEA)

GSEA is widely used to study gene expression data arranged in known molecular pathways, allowing for a better detection of biologically relevant changes^143–146^. However, classical GSEA has some inherent limitations, including the inability to handle datasets of different sizes and complex experimental designs^147^. A subset of GSEA, the generally applicable gene set enrichment (GAGE) for the pathways analysis addressed these limitations^147^, enabling to choose independent pathways databases to be analyzed depending on research goals, hence consistently outperforming classical GSEA methods^147^. The KEGG and GO pathway enrichment analyses were performed on normalized and log2-transformed counts by the GAGE package^147^, using two-sample Student’s t-test for group comparison of differential expression of gene sets. The FDR cut-off was set at 0.05 for the KEGG pathways and 0.01 for the GO pathways. The FDR for GO pathways was reduced to 0.01 since it has more pathways than KEGG (~ 40 000 vs. ~ 500) and 0.05 FDR for GO results in large amount of significantly altered pathways, whereas we wanted to focus on the most significant pathways. Sets were additionally grouped by similarity of core genes that contribute to pathways enrichment by function esset.grp (cutoff p-value 10e−10), to provide clear representation of their functional connection, choosing the most enriched gene sets among functionally related sets when needed^148^.

### Topological analyses

Topological analyses were performed using the Cytoscape software for integrated models of biomolecular interaction networks version 3.8.0^149,150^. The protein–protein interaction (PPI) networks were constructed based on all significantly different genes from all analyses using the STRING (Search Tool for the Retrieval of Interacting Genes/Proteins) database^151^ (https://string-db.org/cgi/input.pl), with significant PPIs and the addition of 20 proteins suggested by STRING analyses as being closely related to the network (i.e., excluding text mining, and all proteins that had no connections to the main network), with the level of minimal required interaction score in STRING at db = 0.15. The resultant PPI networks were analyzed by the Cytoscape application cytoHubba^152^ to probe essential nodes/hubs in-network for top 10 degree nodes, top 10 bottleneck nodes or top 10 nodes by the Double Screening Scheme (DSS), combining Density of Maximum Neighborhood Component (DMNC) and Maximum Neighborhood Component (MNC)^152^. The degree of the node *v* was defined as the number of edges of node *v*, thus representing the number of a protein’s connections to other proteins. The bottleneck nodes were determined using the betweenness centrality of the node, based on the measuring of the number of shortest passes going through the node^153^. Bottleneck proteins are likely to be essential in the network functioning as connectors bridge-like proteins^154^. MNC of the node *v* was defined as a size of the maximum connected component of subnetwork *N(v)* constructed by nodes adjacent to *v*. DMNC of the node v was defined as *E/N*^*ε*^ where *N* is node number and* E* is edge number of MNC*(v)*, and *ε* is defined as 1.7. DSS was further calculated as follows: for *n* most possible essential proteins that were expected in the output (*n* is an empirical value), *2n* top-ranked proteins were selected by DMNC method. The resulting proteins were then ranked by MNC value, and top *n* proteins picked. DSS (DMNC||MNC) method is an effective method to identify essential proteins^155^. The gene co-expression networks were also constructed in the same way, as PPI networks using the Cytoscape application GeneMANIA^156–158^ (https://genemania.org/) for zebrafish gene co-expression data, and analyzed similarly to the PPI networks.

Finally, to visualize the resulting networks, we used power graph analysis (PGA)—a novel method of analysis and representations of complex networks, in which usual nodes and edges are replaced with power nodes and power edges constructed from common topological structures: ‘cliques’ (set of nodes with an edge between each pair, represented as a loop), ‘bicliques’ (two sets of nodes with edge between every member of other set; represented as two power nodes connected with power edge) and ‘stars’ (set of nodes connected to single node; represented as power node connected to usual node)^159^. PGA is an effective tool to compress information contained in the network and to improve its visual representation, helping to focus on key ‘hub’ nodes^159^. The present study constructed power graphs for both STRING and GeneMANIA networks, aiming to improve the overall visual representation of molecular networks revealed here (Supplementary Figs. S4, S5).

### Ethical confirmation statements

Animal experiments were approved by IACUC of St. Petersburg State University and fully adhered to the National and Institutional guidelines and regulations on animal experimentation, as well as the 3Rs principles of humane animal experimentation.

## Supplementary information

Supplementary Information.

## Data Availability

The datasets generated and/or analyzed during the current study are available from the corresponding authors upon reasonable requests.
