# Supplementary material for: Understanding complex dynamics of behavioral, neurochemical and transcriptomic changes induced by prolonged chronic unpredictable stress in zebrafish
Source: Sci Rep. 2020 Nov 17;10:19981. doi: 10.1038/s41598-020-75855-3 (PMC7673038; doi:10.1038/s41598-020-75855-3)
Supplement: Supplementary file 1 — Supplementary Information. [file 41598_2020_75855_MOESM1_ESM.pdf]

<u>**SUPPLEMENTARY ONLINE MATERIALS**</u>

Paper: Understanding complex dynamics of behavioral, neurochemical and transcriptomic changes induced by prolonged chronic unpredictable stress in zebrafish

Konstantin A. Demin*[1,2], Anton M. Lakstygal[2], Nataliya A. Krotova[1,2], Alexey Masharsky[3], Natsuki Tagawa[4], Maria V. Chernysh[2], Nikita P. Ilyin[2], Alexander S. Taranov[2], David S. Galstyan[2,5], Ksenia A. Nikolaeva[1], Nataliia A. Levchenko[2], Tatiana O. Kolesnikova[2], Mikael S. Mor[2], Marina L. Vasyutina[1], Evgeniya V. Efimova[2], Nataliia Katolikova[2,7], Andrey D. Prjibelski[8], Raul R. Gainetdinov[2], Murilo S. de Abreu[9], Tamara G. Amstislavskaya[10,11], Tatyana Strekalova[12] and Allan V. Kalueff*[6,13]

[1]Institute of Experimental Medicine, Almazov National Medical Research Centre, Ministry of Healthcare of Russian Federation, St. Petersburg, Russia
[2]Institute of Translational Biomedicine, St. Petersburg State University, St. Petersburg, Russia
[3]Core Facility Centre for Molecular and Cell Technologies, St. Petersburg State University
[4]Department of Biophysics and Biochemistry, Graduate School of Science, University of Tokyo, Tokyo, Japan
[5]Laboratory of Preclinical Bioscreening, Granov Russian Research Center of Radiology and Surgical Technologies, Ministry of Healthcare of Russian Federation, Pesochny, Russia
[6]School of Pharmacy, Southwest University, Chongqing, China;
[7]Institute of Cytology RAS, Saint-Petersburg, Russia
[8]Center for Algorithmic Biotechnology, Institute of Translational Biomedicine, St. Petersburg State University
[9]University of Passo Fundo, Passo Fundo, Brazil
[10]Laboratory of Biopsychiatry, Scientific Research Institute of Physiology and Basic Medicine, Novosibirsk, Russia
[11]School of Medicine and Psychology, Novosibirsk State University, Novosibirsk, Russia
[12]University of Maastricht, Maastricht, Netherlands
[13] Ural Federal University, Ekaterinburg, Russia

[*]Co-corresponding authors:

Allan V. Kalueff, Ph.D., School of Pharmacy, Southwest University, Chongqing, China.
Tel/Fax: +1-240-899-9571 E-mail: avkalueff@gmail.com
Konstantin A. Demin, Institute of Translational Biomedicine SPBU, University Emb. 7-9, St. Petersburg, Russia. E-mail: deminkasci@gmail.com

Data availability

The datasets generated and/or analyzed in the present study are available from the original article, supplementary materials, or the corresponding author (upon reasonable requests, for use in collaborative research projects and/or for joint publications resulting from such projects).

Supplementary Figure S1. The MA-plots (Bland–Altman plots) of the estimated fold change over average expression strength, representing whole-brain gene expression changes induced in adult zebrafish by a 5-week chronic unpredictable stress (CUS), assessed by RNA-sequencing (n=6). Data was normalized using the rlog function[1]. Graphs were constructed using the ggplot2 R package[2]. Color dots denote genes with statistically significant altered expression: Up – significantly up-regulated genes, down – significantly down-regulated genes, NS – no significant differences.

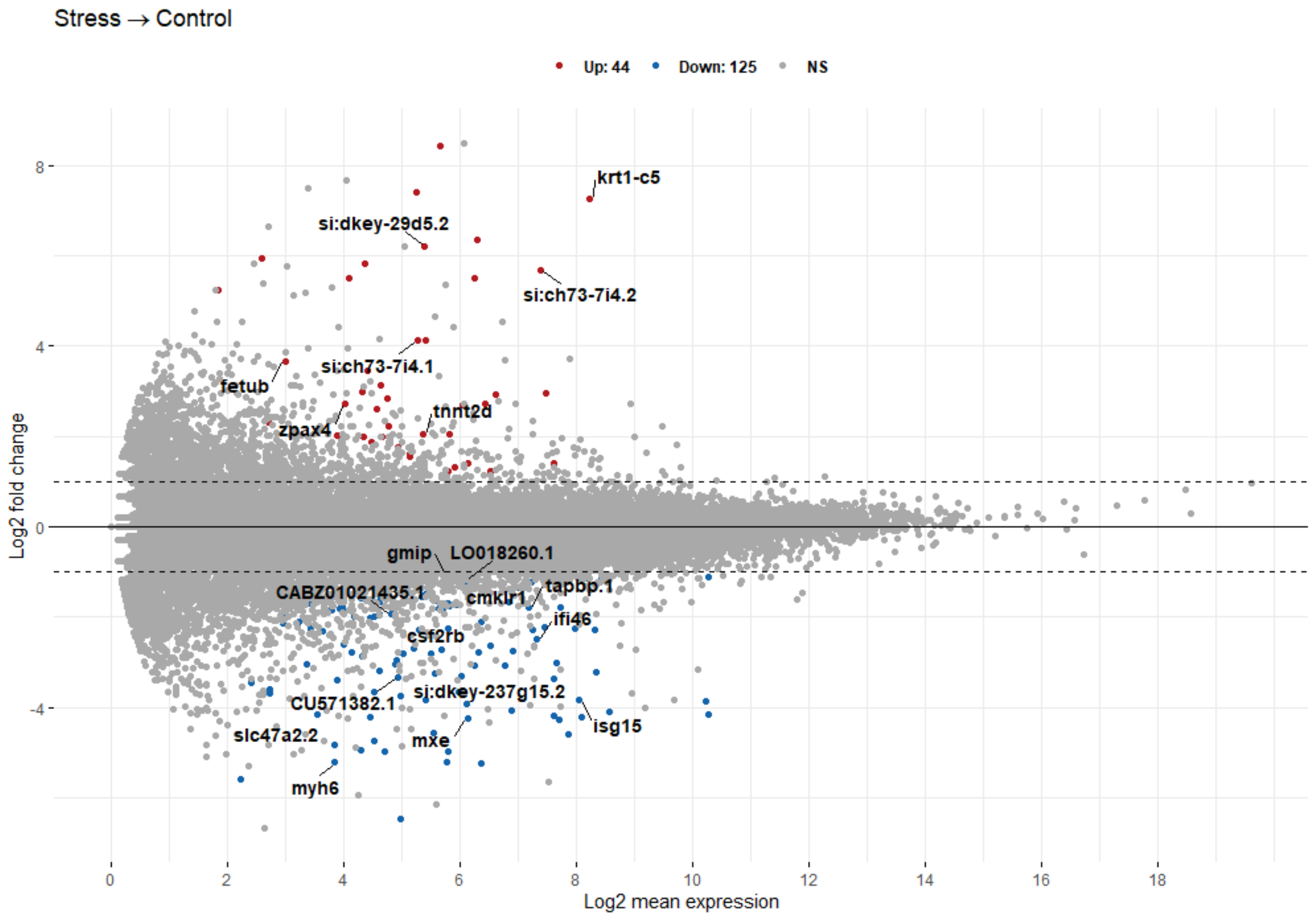

Supplementary Figure S2. The MA-plots (Bland–Altman plots) of the estimated fold change over average expression strength, representing whole-brain gene expression alterations induced by in adult zebrafish by a 5-week chronic unpredictable stress (CUS, as in Supplementary Figure S1) and chronic 0.1 mg/L fluoxetine treatment during the week 5 of CUS, assessed by RNA-sequencing (n=6-7). Data was normalized using the rlog function[1]. Graphs were constructed using the ggplot2 R package[2]. Color dots denote genes with statistically significantly altered expression: Up – significantly up-regulated genes, down – significantly down-regulated genes, NS – no significant differences.

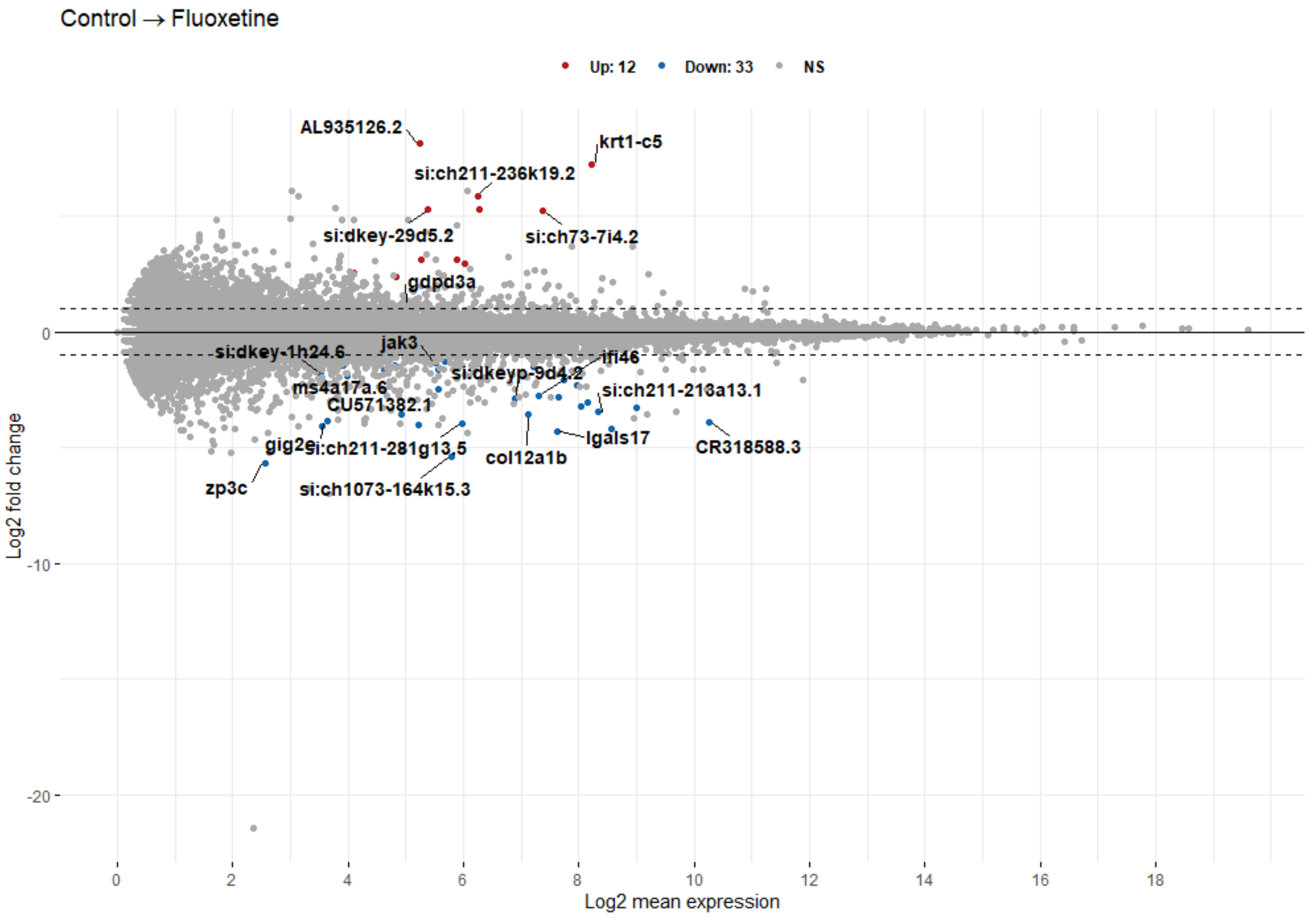

Supplementary Figure S3. The MA-plots (Bland–Altman plots) of the estimated fold change over average expression strength representing whole-brain gene expression changes induced in adult zebrafish subjected to a 5-week chronic unpredictable stress (CUS) with and without chronic 0.1 mg/L fluoxetine treatment during the last CUS week , assessed by RNA-sequencing (n=6-7). Data was normalized using the rlog function[1]. Graphs were constructed using the ggplot2 R package[2]. Color dots denote genes with statistically significantly altered expression: Up – significantly up-regulated genes, down – significantly down-regulated genes, NS – no significant differences.

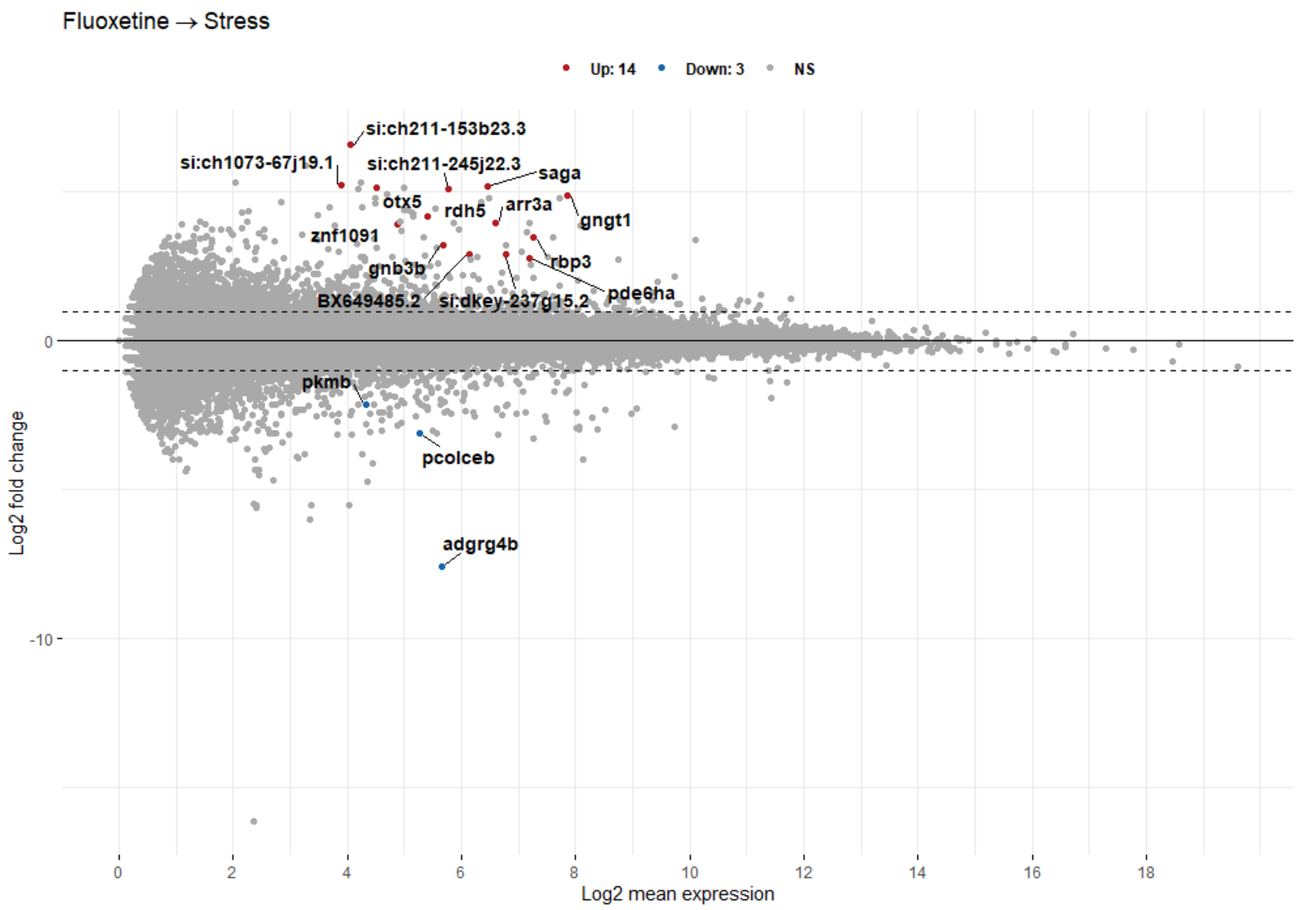

Supplementary Figure S4. Power graph analysis (PGA) of String PPI network. PGA is a novel method of analysis and representations of complex networks in which usual nodes and edges are replaced with power nodes and power edges constructed from common topological structures – cliques (set of nodes with an edge between each pair; represented as a loop), bicliques (two sets of nodes with edge between every member of other set; represented as two power nodes connected with power edge) and stars (set of nodes connected to single node; represented as power node connected to usual node)[3].

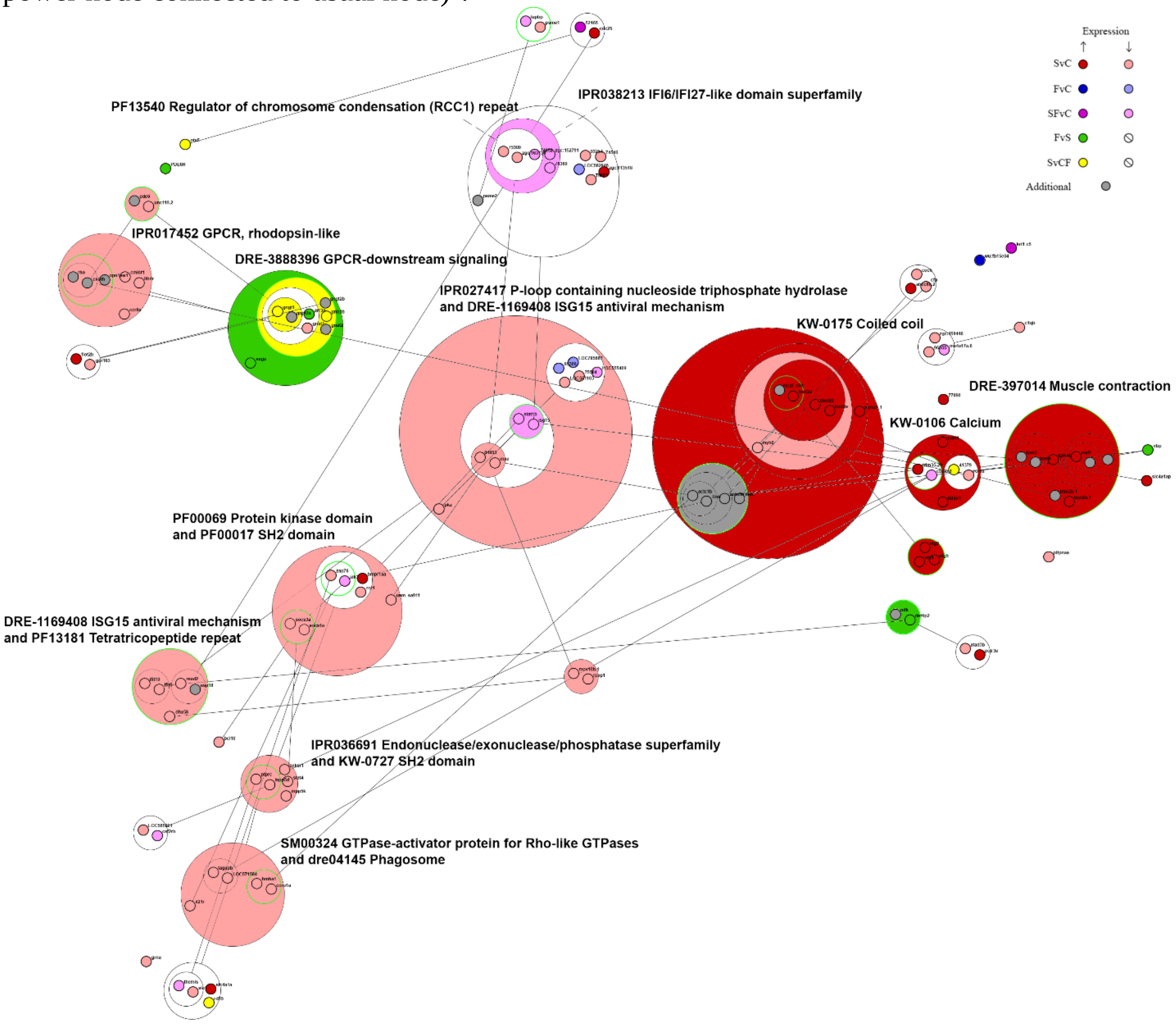

Supplementary Figure S5. Power graph analysis (PGA) of Genemania network. PGA is a novel method of analysis and representations of complex networks in which usual nodes and edges are replaced with power nodes and power edges constructed from common topological structures – cliques (set of nodes with an edge between each pair; represented as a loop), bicliques (two sets of nodes with edge between every member of other set; represented as two power nodes connected with power edge) and stars (set of nodes connected to single node; represented as power node connected to usual node)[3].

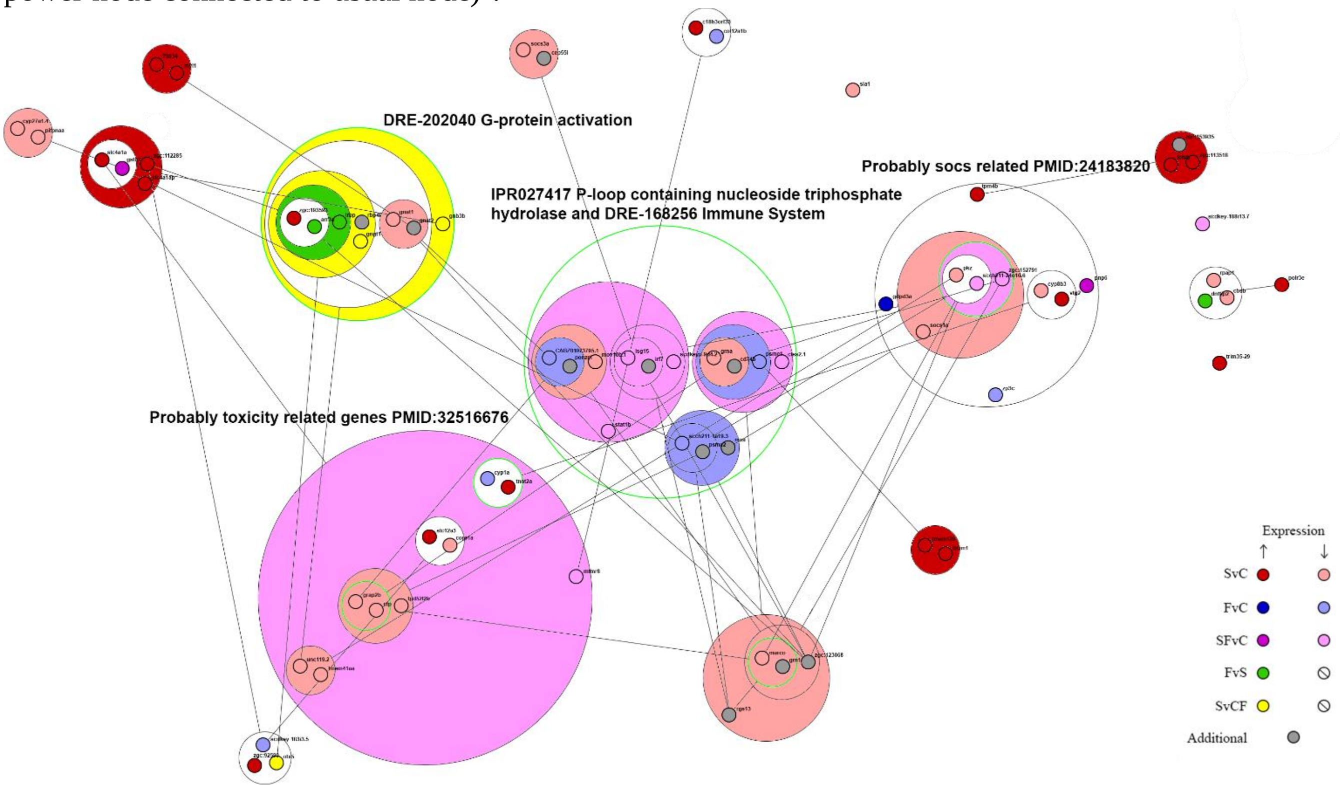

Supplementary Table S1. List of primers used in the study

Gene name		Primer sequence
b-act	F	CATCAGGGTGTCATGGTTGGT
	R	TCTCTTGCTCTGAGCCTCATCA
isg 15	F	ACTTGATTTCGGTGCGACTTGC
	R	GCTGCATCGTCACCGAGTTAT
otx5	F	CGCCGTCGGTTACCCTAACAC
	R	GGTTTTGGAGAACAGCGCCT
saga	F	GTAGATCCAGTGGACGGTGTC
	R	GTCGTCATCACGGCCATACC
tpm4b	F	TCGCGTTTGCGATGATGTCTG
	R	CTGCTACATCTCCTTCAGCTTTCT

Supplementary Table S2. Results of Generalized Linear Model (GZLM) fits using week, group and their interaction effects as 'predictors', to compare stress and control group at weeks 1-5. The corrected Akaike information criterion (AICc) was used to choose the 'best fit' model among Gaussian distribution (identity link), Poisson distribution (with log link), Gamma distribution (inverse and log links) and Inverse Gaussian distribution (with inverse and log links) for the novel tank test (NTT), light-dark test (LDT), shoaling test (ST) and the zebrafish tail immobilization test (ZTI), also see Supplementary Tables S2-S5 and Table 2 in the main text for ANOVA data and post-hoc tests results. 5-HIAA - 5-hydroxyindoleacetic acid, DOPAC - 3,4-dihydroxyphenylacetic acid, HVA - homovanillic acid.

Predictor	b	95% CI	$t(170)$	p
**NTT distance traveled, cm Gauss distribution identity link**				
Intercept	967.68	[809.91, 1,125.45]	12.02	< .001
Week2	187.50	[−35.63, 410.62]	1.65	.101
Week3	311.84	[88.72, 534.96]	2.74	.007
Week4	313.39	[72.39, 554.39]	2.55	.012
Week5	37.86	[−203.14, 278.85]	0.31	.759
GroupStress	538.17	[315.05, 761.29]	4.73	< .001
Week2 × GroupStress	30.89	[−284.65, 346.43]	0.19	.848
Week3 × GroupStress	-352.43	[−667.97, −36.89]	-2.19	.030
Week4 × GroupStress	-339.79	[−680.61, 1.03]	-1.95	.052
Week5 × GroupStress	-137.98	[−478.80, 202.84]	-0.79	.429
**NTT time spent not moving, s inverted Gauss distribution log link**				
Intercept	3.75	[3.22, 4.98]	10.43	< .001
Week2	-0.53	[−1.81, 0.40]	-1.18	.241
Week3	-0.77	[−2.04, 0.07]	-1.77	.079
Week4	-0.99	[−2.26, −0.13]	-2.26	.025
Week5	-0.07	[−1.40, 1.54]	-0.13	.896
GroupStress	-0.84	[−2.10, −0.02]	-1.95	.053
Week2 × GroupStress	-0.38	[−1.46, 0.96]	-0.71	.480
Week3 × GroupStress	1.17	[0.07, 2.56]	2.03	.043
Week4 × GroupStress	0.73	[−0.35, 2.10]	1.32	.190
Week5 × GroupStress	0.19	[−1.50, 1.65]	0.28	.777
**NTT time spent in top, s Gauss distribution identity link**				
Intercept	265.35	[239.39, 291.32]	20.03	< .001
Week2	-129.30	[-166.03, -92.58]	-6.90	< .001
Week3	-69.49	[-106.22, -32.77]	-3.71	< .001
Week4	-108.22	[-147.89, -68.55]	-5.35	< .001
Week5	-100.75	[-140.41, -61.08]	-4.98	< .001
GroupStress	-69.14	[-105.87, -32.42]	-3.69	< .001
Week2 × GroupStress	-23.19	[-75.12, 28.75]	-0.87	.383
Week3 × GroupStress	-78.34	[-130.28, -26.40]	-2.96	.004
Week4 × GroupStress	-28.01	[-84.11, 28.09]	-0.98	.329
Week5 × GroupStress	-69.98	[-126.08, -13.88]	-2.45	.016
**NTT top entries Gauss distribution identity link**				
Intercept	4.35	[0.63, 8.07]	2.29	.023
Week2	9.70	[4.44, 14.96]	3.61	< .001
Week3	9.70	[4.44, 14.96]	3.61	< .001
Week4	11.05	[5.37, 16.73]	3.81	< .001
Week5	3.92	[−1.77, 9.60]	1.35	.179
GroupStress	9.45	[4.19, 14.71]	3.52	.001
Week2 × GroupStress	-13.50	[−20.94, −6.06]	-3.55	< .001
Week3 × GroupStress	-12.15	[−19.59, −4.71]	-3.20	.002
Week4 × GroupStress	-13.72	[−21.76, −5.68]	-3.34	.001
Week5 × GroupStress	-12.58	[−20.62, −4.54]	-3.07	.003
**NTT latency to top, s Gauss distribution identity link**				
Intercept	28.97	[−6.82, 64.76]	1.59	.114
Week2	26.05	[−24.56, 76.67]	1.01	.315
Week3	26.15	[−24.46, 76.77]	1.01	.313
Week4	25.64	[−29.03, 80.31]	0.92	.359
Week5	17.29	[−37.38, 71.96]	0.62	.536
GroupStress	19.34	[−31.28, 69.95]	0.75	.455
Week2 × GroupStress	63.67	[−7.91, 135.25]	1.74	.083
Week3 × GroupStress	46.57	[−25.01, 118.14]	1.28	.204
Week4 × GroupStress	5.08	[−72.23, 82.40]	0.13	.898
Week5 × GroupStress	111.87	[34.55, 189.18]	2.84	.005
**LDT time spent in light, s Gauss distribution identity link**				
Intercept	236.30	[198.40, 274.20]	12.22	< .001

Week2	-41.80	[−95.40, 11.80]	-1.53	.128
Week3	-53.75	[−107.35, −0.15]	-1.97	.051
Week4	-77.50	[−135.40, −19.60]	-2.62	.009
Week5	-82.23	[−140.13, −24.34]	-2.78	.006
GroupStress	-141.20	[−194.80, −87.60]	-5.16	< .001
Week2 × GroupStress	29.10	[−46.70, 104.90]	0.75	.453
Week3 × GroupStress	81.05	[5.25, 156.85]	2.10	.038
Week4 × GroupStress	143.40	[61.52, 225.28]	3.43	.001
Week5 × GroupStress	122.53	[40.66, 204.41]	2.93	.004
**LDT number of light entries Gauss distribution identity link**				
Intercept	6.50	[3.82, 9.18]	4.75	< .001
Week2	0.20	[−3.59, 3.99]	0.10	.918
Week3	4.35	[0.56, 8.14]	2.25	.026
Week4	3.63	[−0.46, 7.73]	1.74	.084
Week5	1.83	[−2.26, 5.93]	0.88	.382
GroupStress	-0.70	[−4.49, 3.09]	-0.36	.718
Week2 × GroupStress	0.85	[−4.51, 6.21]	0.31	.756
Week3 × GroupStress	-2.85	[−8.21, 2.51]	-1.04	.299
Week4 × GroupStress	-2.17	[−7.96, 3.63]	-0.73	.464
Week5 × GroupStress	0.43	[−5.36, 6.23]	0.15	.884
**ST average inter-fish distance, cm Gauss distribution identity link**				
Intercept	1.40	[1.26, 1.56]	17.90	< .001
Week2	0.05	[−0.17, 0.27]	0.42	.672
Week3	0.18	[−0.04, 0.42]	1.59	.113
Week4	0.18	[−0.06, 0.44]	1.46	.146
Week5	0.35	[0.10, 0.63]	2.66	.008
GroupStress	0.00	[−0.22, 0.22]	0.00	.998
Week2 × GroupStress	-0.18	[−0.48, 0.13]	-1.15	.250
Week3 × GroupStress	-0.21	[−0.52, 0.11]	-1.30	.194
Week4 × GroupStress	-0.28	[−0.62, 0.06]	-1.61	.109
Week5 × GroupStress	-0.39	[−0.75, −0.04]	-2.19	.030
**ST distance to the surface, cm Gauss distribution identity link**				
Intercept	11.34	[9.41, 13.27]	11.51	< .001
Week2	-4.92	[−7.65, −2.19]	-3.53	.001
Week3	-3.06	[−5.79, −0.33]	-2.20	.029
Week4	0.75	[−2.19, 3.70]	0.50	.617
Week5	-3.73	[−6.68, −0.78]	-2.48	.014
GroupStress	-5.40	[−8.13, −2.67]	-3.87	< .001
Week2 × GroupStress	11.15	[7.29, 15.01]	5.66	< .001
Week3 × GroupStress	6.34	[2.48, 10.20]	3.22	.001
Week4 × GroupStress	5.12	[0.95, 9.29]	2.41	.017
Week5 × GroupStress	8.44	[4.27, 12.61]	3.97	< .001
**ZTI time spent active, s Gauss distribution identity link**				
Intercept	49.92	[26.42, 73.41]	4.16	< .001
Week2	18.06	[−15.16, 51.28]	1.07	.288
Week3	-18.90	[−52.12, 14.33]	-1.11	.267
Week4	5.23	[−30.66, 41.11]	0.29	.776
Week5	-6.18	[−42.07, 29.70]	-0.34	.736
GroupStress	-34.98	[−68.20, −1.76]	-2.06	.041
Week2 × GroupStress	32.74	[−14.24, 79.72]	1.37	.174
Week3 × GroupStress	101.50	[54.51, 148.48]	4.23	< .001
Week4 × GroupStress	22.28	[−28.46, 73.03]	0.86	.391
Week5 × GroupStress	45.77	[−4.97, 96.52]	1.77	.079
**Dopamine, pg/mg inverted Gauss distribution log link**				
Intercept	5.55	[5.33, 5.83]	44.47	< .001
Week2	-0.12	[−0.47, 0.22]	-0.71	.479
Week3	0.02	[−0.33, 0.38]	0.12	.901
Week4	-0.20	[−0.54, 0.13]	-1.17	.244
Week5	-0.02	[−0.38, 0.33]	-0.14	.887
GroupStress	-0.04	[−0.39, 0.31]	-0.23	.816
Week2 × GroupStress	0.22	[−0.27, 0.71]	0.89	.375
Week3 × GroupStress	0.22	[−0.29, 0.73]	0.85	.400
Week4 × GroupStress	-0.05	[−0.52, 0.41]	-0.22	.828
Week5 × GroupStress	0.25	[−0.25, 0.76]	0.99	.323
**DOPAC/dopamine ratio inverted Gauss distribution inverse link**				
Intercept	9.00	[5.47, 12.52]	5.00	< .001
Week2	-2.57	[−7.19, 2.05]	-1.09	.279
Week3	0.49	[−4.57, 5.54]	0.19	.851
Week4	-1.40	[−6.19, 3.39]	-0.57	.569
Week5	0.29	[−4.74, 5.32]	0.11	.909
GroupStress	0.85	[−4.26, 5.95]	0.33	.745
Week2 × GroupStress	-1.46	[−8.02, 5.09]	-0.44	.663
Week3 × GroupStress	4.68	[−3.06, 12.43]	1.19	.239

Week4 × GroupStress	0.17	[−6.80, 7.13]	0.05	.962
Week5 × GroupStress	-2.29	[−9.35, 4.76]	-0.64	.525
**DOPAC pg/mg inverted Gauss distribution log link**				
Intercept	3.37	[3.02, 3.93]	15.43	< .001
Week2	0.10	[−0.57, 0.79]	0.32	.752
Week3	-0.06	[−0.71, 0.59]	-0.18	.856
Week4	-0.06	[−0.71, 0.58]	-0.19	.849
Week5	-0.14	[−0.79, 0.48]	-0.47	.639
GroupStress	-0.26	[−0.90, 0.33]	-0.91	.368
Week2 × GroupStress	0.36	[−0.53, 1.29]	0.82	.415
Week3 × GroupStress	-0.02	[−0.84, 0.81]	-0.04	.967
Week4 × GroupStress	0.05	[−0.78, 0.88]	0.12	.904
**5-HIAA pg/mg inverted Gauss distribution log link**				
Intercept	5.24	[4.97, 5.63]	32.47	< .001
Week2	-0.28	[−0.73, 0.14]	-1.31	.193
Week3	0.02	[−0.45, 0.49]	0.07	.941
Week4	-0.05	[−0.51, 0.41]	-0.20	.842
Week5	-0.15	[−0.61, 0.29]	-0.69	.489
GroupStress	-0.15	[−0.61, 0.29]	-0.69	.495
Week2 × GroupStress	0.62	[0.00, 1.27]	1.96	.053
Week3 × GroupStress	-0.22	[−0.84, 0.38]	-0.73	.469
Week4 × GroupStress	-0.45	[−1.05, 0.14]	-1.52	.132
Week5 × GroupStress	0.20	[−0.40, 0.82]	0.67	.507
**5-HIAA/serotonin ratio inverted Gauss distribution inverse link**				
Intercept	1.58	[1.03, 2.14]	5.58	< .001
Week2	0.28	[−0.54, 1.10]	0.68	.501
Week3	-0.11	[−0.89, 0.66]	-0.28	.777
Week4	-0.30	[−1.05, 0.45]	-0.78	.436
Week5	-0.07	[−0.85, 0.71]	-0.17	.868
GroupStress	-0.03	[−0.82, 0.75]	-0.09	.932
Week2 × GroupStress	-0.80	[−1.89, 0.28]	-1.45	.151
Week3 × GroupStress	1.46	[0.25, 2.67]	2.36	.020
Week4 × GroupStress	1.06	[−0.09, 2.21]	1.81	.073
Week5 × GroupStress	0.51	[−0.63, 1.65]	0.88	.384
**Serotonin, pg/mg inverted Gauss distribution log link**				
Intercept	5.73	[5.50, 6.04]	42.77	< .001
Week2	-0.09	[−0.47, 0.28]	-0.48	.635
Week3	-0.03	[−0.41, 0.35]	-0.18	.861
Week4	-0.10	[−0.48, 0.27]	-0.53	.596
Week5	-0.14	[−0.51, 0.23]	-0.76	.449
GroupStress	-0.11	[−0.49, 0.26]	-0.61	.544
Week2 × GroupStress	0.10	[−0.41, 0.62]	0.40	.688
Week3 × GroupStress	0.37	[−0.16, 0.92]	1.36	.178
Week4 × GroupStress	-0.01	[−0.51, 0.50]	-0.03	.976
Week5 × GroupStress	0.49	[−0.04, 1.04]	1.82	.072
**HVA to dopamine ratio inverted Gauss distribution inverse link**				
Intercept	37.70	[20.26, 55.14]	4.24	< .001
Week2	6.21	[−19.45, 31.86]	0.47	.637
Week3	19.85	[−7.87, 47.57]	1.40	.164
Week4	-2.00	[−26.33, 22.33]	-0.16	.872
Week5	1.31	[−23.57, 26.19]	0.10	.918
GroupStress	13.78	[−13.04, 40.61]	1.01	.317
Week2 × GroupStress	-26.30	[−62.72, 10.13]	-1.42	.160
Week3 × GroupStress	-5.22	[−46.65, 36.22]	-0.25	.806
Week4 × GroupStress	6.35	[−31.83, 44.54]	0.33	.745
Week5 × GroupStress	13.55	[−26.06, 53.16]	0.67	.504
**HVA, pg/mg inverted Gauss distribution inverse link**				
Intercept	0.15	[0.07, 0.22]	3.99	< .001
Week2	0.05	[−0.06, 0.15]	0.81	.418
Week3	0.08	[−0.03, 0.20]	1.41	.163
Week4	0.03	[−0.08, 0.13]	0.47	.638
Week5	0.03	[−0.08, 0.14]	0.56	.579
GroupStress	0.08	[−0.03, 0.20]	1.42	.159
Week2 × GroupStress	-0.13	[−0.29, 0.03]	-1.62	.109
Week3 × GroupStress	-0.10	[−0.27, 0.07]	-1.12	.267
Week4 × GroupStress	0.04	[−0.13, 0.22]	0.50	.620
Week5 × GroupStress	0.00	[−0.17, 0.17]	0.01	.990
**Norepinephrine, pg/mg Gauss distribution identity link**				
Intercept	913.44	[678.49, 1,148.38]	7.62	< .001
Week2	-163.89	[−496.16, 168.37]	-0.97	.336
Week3	-106.53	[−438.79, 225.73]	-0.63	.531
Week4	-197.99	[−530.25, 134.27]	-1.17	.246
Week5	105.58	[−226.69, 437.84]	0.62	.535

GroupStress	-78.08	[−410.34, 254.18]	-0.46	.646
Week2 × GroupStress	254.60	[−215.29, 724.49]	1.06	.291
Week3 × GroupStress	463.23	[−6.67, 933.12]	1.93	.056
Week4 × GroupStress	100.21	[−369.68, 570.11]	0.42	.677
Week5 × GroupStress	310.81	[−159.08, 780.71]	1.30	.198

Supplementary Table S3. Post-hoc Tukey's test results for significant group predictor ANOVA data pair-wise comparison using the Generalized Linear Model (GZLM) with week, group and their interaction effects as predictors comparing stress and control group at 1-5 weeks of the chronic unpredictable stress (CUS) for the novel tank test (NTT), light-dark test (LDT), shoaling test (ST) and zebrafish tail immobilization (ZTI) test, also see Supplementary Tables S1, S3-S5 and Tables 2-3 in the main text for GZLM, ANOVA and other post-hoc test results. 5-HIAA - 5-hydroxyindoleacetic acid.

**Endpoints**	ΔM	**95% CI**	$z.ratio$	p
NTT Distance traveled, cm	-378.31	$[-484.54, -272.08]$	-6.98	< .001
NTT Time spent not moving, s	0.50	[0.15, 0.85]	2.80	.005
NTT Time spent in top, s	109.05	[91.56, 126.53]	12.22	< .001
NTT Latency to enter the top, s	-64.77	$[-88.87, -40.68]$	-5.27	< .001
LDB Time spent in light, s	65.98	[40.46, 91.50]	5.07	< .001
ST inter-fish distance, cm	0.21	[0.10, 0.32]	3.87	< .001
5-HIAA/serotonin ratio	-0.41	$[-0.78, -0.04]$	-2.18	.029

Supplementary Table S4. Results of post-hoc Tukey's test for significant week predictor ANOVA data pair-wise comparison using Generalized Linear Model (GZLM) with week, group and their interaction effects as 'predictors', comparing stress and control group at 1-5 weeks of the chronic unpredictable stress (CUS) for the novel tank test (NTT), the light-dark test (LDT), shoaling test (ST) and the zebrafish tail immobilization test (ZTI), also see Supplementary Tables S1-S2, S4-S5 and Table 2 in the main text for GZLM, ANOVA and other post-hoc test results. DOPAC - 3,4-dihydroxyphenylacetic acid.

Comparisons (week vs week)	ΔM	95% CI	$z.ratio$	p
**NTT Distance traveled, cm**				
1 - 2	-202.94	[−360.71, −45.17]	-2.52	.012
1 - 3	-135.63	[−293.40, 22.14]	-1.68	.092
1 - 4	-143.49	[−313.90, 26.92]	-1.65	.099
1 - 5	31.14	[−139.28, 201.55]	0.36	.720
2 - 3	67.31	[−90.46, 225.08]	0.84	.403
2 - 4	59.44	[−110.97, 229.86]	0.68	.494
2 - 5	234.07	[63.66, 404.49]	2.69	.007
3 - 4	-7.87	[−178.28, 162.55]	-0.09	.928
3 - 5	166.76	[−3.65, 337.17]	1.92	.055
4 - 5	174.63	[−7.55, 356.81]	1.88	.060
**NTT Time spent not moving, s**				
1 – 2	0.72	[0.20, 1.24]	2.71	.007
1 – 3	0.19	[−0.38, 0.75]	0.65	.517
1 – 4	0.63	[0.09, 1.17]	2.27	.023
1 – 5	-0.02	[−0.67, 0.62]	-0.07	.945
2 – 3	-0.54	[−1.02, −0.05]	-2.18	.029
2 – 4	-0.09	[−0.55, 0.37]	-0.40	.688
2 – 5	-0.75	[−1.32, −0.17]	-2.54	.011
3 – 4	0.44	[−0.06, 0.95]	1.72	.085
3 – 5	-0.21	[−0.82, 0.40]	-0.67	.503
4 – 5	-0.65	[−1.24, −0.06]	-2.15	.031
**NTT Time spent in top, s**				
1 – 2	140.90	[114.93, 166.87]	10.63	< .001
1 – 3	108.66	[82.69, 134.63]	8.20	< .001
1 – 4	122.22	[94.17, 150.27]	8.54	< .001
1 – 5	135.74	[107.69, 163.79]	9.48	< .001
2 – 3	-32.23	[−58.20, −6.27]	-2.43	.015
2 – 4	-18.67	[−46.72, 9.38]	-1.30	.192
2 – 5	-5.16	[−33.21, 22.89]	-0.36	.718
3 – 4	13.56	[−14.49, 41.61]	0.95	.343
3 – 5	27.08	[−0.97, 55.12]	1.89	.059
4 – 5	13.51	[−16.47, 43.50]	0.88	.377
**NTT Number of top entries**				
1 – 2	-2.95	[−6.67, 0.77]	-1.55	.120
1 – 3	-3.63	[−7.35, 0.10]	-1.91	.056
1 – 4	-4.19	[−8.21, −0.17]	-2.04	.041
1 – 5	2.37	[−1.64, 6.39]	1.16	.247
2 – 3	-0.68	[−4.40, 3.05]	-0.36	.722
2 – 4	-1.24	[−5.26, 2.78]	-0.61	.545
2 – 5	5.32	[1.31, 9.34]	2.60	.009
3 – 4	-0.57	[−4.59, 3.45]	-0.28	.782
3 – 5	6.00	[1.98, 10.02]	2.93	.003
4 – 5	6.57	[2.27, 10.86]	3.00	.003
**NTT Latency to enter top, s**				
1 - 2	-57.89	[−93.68, −22.10]	-3.17	.002
1 - 3	-49.43	[−85.22, −13.65]	-2.71	.007
1 - 4	-28.18	[−66.84, 10.48]	-1.43	.153
1 - 5	-73.22	[−111.88, −34.56]	-3.71	< .001
2 - 3	8.45	[−27.34, 44.24]	0.46	.644
2 - 4	29.71	[−8.95, 68.37]	1.51	.132
2 - 5	-15.33	[−53.99, 23.32]	-0.78	.437
3 - 4	21.26	[−17.40, 59.91]	1.08	.281
3 - 5	-23.79	[−62.44, 14.87]	-1.21	.228
4 - 5	-45.04	[−86.37, −3.72]	-2.14	.033
**ST Distance to surface, cm**				
1 - 2	-0.66	[−2.59, 1.27]	-0.67	.504
1 - 3	-0.11	[−2.04, 1.82]	-0.11	.909
1 - 4	-3.31	[−5.40, −1.23]	-3.11	.002

1 - 5	-0.49	[−2.58, 1.59]	-0.46	.645
2 - 3	0.55	[−1.38, 2.48]	0.56	.579
2 - 4	-2.65	[−4.74, −0.57]	-2.50	.013
2 - 5	0.17	[−1.92, 2.25]	0.16	.874
3 - 4	-3.20	[−5.29, −1.12]	-3.01	.003
3 - 5	-0.38	[−2.46, 1.71]	-0.36	.722
4 - 5	2.82	[0.59, 5.05]	2.48	.013
**ZTI Time spent active, s**				
1 - 2	-34.43	[−57.92, −10.94]	-2.87	.004
1 - 3	-31.85	[−55.34, −8.36]	-2.66	.008
1 - 4	-16.37	[−41.74, 9.00]	-1.26	.206
1 - 5	-16.70	[−42.08, 8.67]	-1.29	.197
2 - 3	2.58	[−20.92, 26.07]	0.21	.830
2 - 4	18.06	[−7.31, 43.43]	1.39	.163
2 - 5	17.72	[−7.65, 43.10]	1.37	.171
3 - 4	15.48	[−9.89, 40.86]	1.20	.232
3 - 5	15.15	[−10.22, 40.52]	1.17	.242
4 - 5	-0.33	[−27.46, 26.79]	-0.02	.981
**Dopamine, pg/mg**				
1 - 2	0.01	[−0.23, 0.25]	0.10	.923
1 - 3	-0.13	[−0.38, 0.12]	-1.02	.309
1 - 4	0.22	[−0.01, 0.45]	1.90	.057
1 - 5	-0.10	[−0.35, 0.15]	-0.80	.426
2 - 3	-0.14	[−0.39, 0.11]	-1.11	.266
2 - 4	0.21	[−0.02, 0.44]	1.80	.071
2 - 5	-0.11	[−0.36, 0.14]	-0.89	.372
3 - 4	0.35	[0.11, 0.59]	2.90	.004
3 - 5	0.03	[−0.23, 0.29]	0.22	.825
4 - 5	-0.32	[−0.56, −0.09]	-2.68	.007
**DOPAC/dopamine ratio**				
1 - 2	3.30	[0.02, 6.58]	1.97	.049
1 - 3	-2.83	[−6.70, 1.04]	-1.43	.152
1 - 4	1.31	[−2.17, 4.79]	0.74	.460
1 - 5	0.85	[−2.67, 4.38]	0.47	.635
2 - 3	-6.13	[−9.69, −2.56]	-3.37	.001
2 - 4	-1.99	[−5.12, 1.15]	-1.24	.214
2 - 5	-2.45	[−5.63, 0.74]	-1.50	.133
3 - 4	4.14	[0.39, 7.89]	2.16	.031
3 - 5	3.68	[−0.11, 7.48]	1.90	.057
4 - 5	-0.46	[−3.85, 2.94]	-0.26	.792
**Norepinephrine, pg/mg**				
1 - 2	36.59	[−198.35, 271.54]	0.31	.760
1 - 3	-125.08	[−360.03, 109.86]	-1.04	.297
1 - 4	147.88	[−87.06, 382.83]	1.23	.217
1 - 5	-260.98	[−495.93, −26.04]	-2.18	.029
2 - 3	-161.68	[−396.62, 73.27]	-1.35	.177
2 - 4	111.29	[−123.65, 346.24]	0.93	.353
2 - 5	-297.58	[−532.52, −62.63]	-2.48	.013
3 - 4	272.97	[38.02, 507.91]	2.28	.023
3 - 5	-135.90	[−370.85, 99.04]	-1.13	.257
4 - 5	-408.87	[−643.81, −173.92]	-3.41	.001

Supplementary Table S5. Results of post-hoc Tukey's test for significant group x week interaction predictor ANOVA data pair-wise comparison of group differences within the same week, using Generalized Linear Model (GZLM) with week, group and their interaction effects as predictors to compare stress and control group at weeks 1-5 for the novel tank test (NTT), the light-dark test (LDT), shoaling test (ST) and the zebrafish tail immobilization test (ZTI). 5-HIAA - 5-hydroxyindoleacetic acid.

Comparisons (group vs. group within the same week)	ΔM	95% CI	$z.ratio$	p
**NTT Time spent not moving, s**				
1,Control - 1,Stress	0.84	[0.00, 1.68]	1.95	.051
2,Control - 2,Stress	1.22	[0.60, 1.83]	3.88	< .001
3,Control - 3,Stress	-0.33	[−1.07, 0.42]	-0.86	.388
4,Control - 4,Stress	0.11	[−0.57, 0.79]	0.32	.751
5,Control - 5,Stress	0.65	[−0.32, 1.62]	1.32	.186
**NTT Time spent in top, s**				
1,Control - 1,Stress	69.14	[32.42, 105.87]	3.69	< .001
2,Control - 2,Stress	92.33	[55.60, 129.05]	4.93	< .001
3,Control - 3,Stress	147.48	[110.75, 184.21]	7.87	< .001
4,Control - 4,Stress	97.15	[54.74, 139.56]	4.49	< .001
5,Control - 5,Stress	139.13	[96.72, 181.53]	6.43	< .001
**NTT Number of top entries**				
1,Control - 1,Stress	-9.45	[−14.71, −4.19]	-3.52	< .001
2,Control - 2,Stress	4.05	[−1.21, 9.31]	1.51	.131
3,Control - 3,Stress	2.70	[−2.56, 7.96]	1.01	.315
4,Control - 4,Stress	4.27	[−1.81, 10.34]	1.38	.169
5,Control - 5,Stress	3.13	[−2.94, 9.21]	1.01	.312
**NTT Latency to enter the top, s**				
1,Control - 1,Stress	-19.34	[−69.95, 31.28]	-0.75	.454
2,Control - 2,Stress	-83.01	[−133.62, −32.39]	-3.21	.001
3,Control - 3,Stress	-65.90	[−116.52, −15.29]	-2.55	.011
4,Control - 4,Stress	-24.42	[−82.86, 34.03]	-0.82	.413
5,Control - 5,Stress	-131.20	[−189.65, −72.76]	-4.40	< .001
**LDT Time spent in light, s**				
1,Control - 1,Stress	141.20	[87.60, 194.80]	5.16	< .001
2,Control - 2,Stress	112.10	[58.50, 165.70]	4.10	< .001
3,Control - 3,Stress	60.15	[6.55, 113.75]	2.20	.028
4,Control - 4,Stress	-2.20	[−64.09, 59.69]	-0.07	.944
5,Control - 5,Stress	18.67	[−43.23, 80.56]	0.59	.554
**ST Distance to surface, cm**				
1,Control - 1,Stress	5.40	[2.67, 8.13]	3.87	< .001
2,Control - 2,Stress	-5.76	[−8.49, −3.03]	-4.13	< .001
3,Control - 3,Stress	-0.95	[−3.68, 1.78]	-0.68	.497
4,Control - 4,Stress	0.28	[−2.87, 3.43]	0.17	.863
5,Control - 5,Stress	-3.04	[−6.19, 0.11]	-1.89	.059
**ZTI Time spent active, s**				
1,Control - 1,Stress	34.98	[1.76, 68.20]	2.06	.039
2,Control - 2,Stress	2.24	[−30.98, 35.46]	0.13	.895
3,Control - 3,Stress	-66.52	[−99.74, −33.30]	-3.92	< .001
4,Control - 4,Stress	12.69	[−25.67, 51.05]	0.65	.517
5,Control - 5,Stress	-10.80	[−49.16, 27.56]	-0.55	.581
**5-HIAA, pg/mg**				
1,Control - 1,Stress	0.15	[−0.28, 0.58]	0.69	.493
2,Control - 2,Stress	-0.47	[−0.91, −0.02]	-2.07	.038
3,Control - 3,Stress	0.37	[−0.04, 0.79]	1.76	.078
4,Control - 4,Stress	0.60	[0.22, 0.99]	3.05	.002
5,Control - 5,Stress	-0.05	[−0.47, 0.37]	-0.25	.803
**5-HIAA/serotonin ratio**				
1,Control - 1,Stress	0.03	[−0.75, 0.82]	0.09	.932
2,Control - 2,Stress	0.84	[0.08, 1.59]	2.18	.029
3,Control - 3,Stress	-1.43	[−2.35, −0.50]	-3.03	.002
4,Control - 4,Stress	-1.03	[−1.87, −0.19]	-2.40	.016
5,Control - 5,Stress	-0.47	[−1.30, 0.35]	-1.12	.261

Supplementary Table S6. Results of generalized Linear Model (GZLM) fits using group as ‘predictor’ to compare control, stress and fluoxetine groups at 5th week of chronic unpredictable stress (CUS). The corrected Akaike information criterion (AIC) was used to choose the ‘best fit’ model among Gaussian distribution (identity link), Poisson distribution (with log link), Gamma distribution (inverse and log links) and Inverse Gaussian distribution (with inverse and log links) for the novel tank test (NTT), the light-dark test (LDT), shoaling test (ST) and the zebrafish tail immobilization test (ZTI), also see Table 3 in the main text for ANOVA and post-hoc tests results. 5-HIAA - 5-hydroxyindoleacetic acid, DOPAC - 3,4-dihydroxyphenylacetic acid, HVA - homovanillic acid.

**Predictor**	***b***	**95% CI**	$t(42)$	***p***
**ZTI Time spent active, s Gauss distribution identity link**				
Intercept	43.73	[18.90, 68.56]	3.45	.001
GroupFluoxetine	2.24	[−32.88, 37.36]	0.13	.901
GroupStress	10.80	[−24.32, 45.92]	0.60	.550
**NTT Distance traveled, cm Gauss distribution identity link**				
Intercept	1,005.54	[835.31, 1,175.77]	11.58	< .001
GroupFluoxetine	28.33	[-212.41, 269.06]	0.23	.819
GroupStress	400.19	[159.45, 640.93]	3.26	.002
**ST Inter-fish distance, cm Gamma distribution log link**				
Intercept	1.75	[1.58, 1.94]	19.18	< .001
GroupFluoxetine	-0.09	[−0.34, 0.17]	-0.67	.508
GroupStress	-0.39	[−0.64, −0.14]	-3.01	.004
**NTT Time spent not moving, s inverted Gauss distribution log link**				
Intercept	3.68	[3.20, 4.64]	11.72	< .001
GroupFluoxetine	-0.53	[−1.55, 0.27]	-1.33	.192
GroupStress	-0.65	[−1.67, 0.10]	-1.69	.099
**NTT Time spent in top, s Gauss distribution identity link**				
Intercept	164.61	[131.03, 198.19]	9.61	< .001
GroupFluoxetine	-41.10	[−88.59, 6.39]	-1.70	.097
GroupStress	-139.13	[−186.61, −91.64]	-5.74	< .001
**NTT Number of top entries Gauss distribution identity link**				
Intercept	8.27	[5.10, 11.43]	5.12	< .001
GroupFluoxetine	0.27	[−4.21, 4.74]	0.12	.908
GroupStress	-3.13	[−7.61, 1.34]	-1.37	.178
**NTT Latency to enter the top, s Gauss distribution identity link**				
Intercept	46.26	[1.49, 91.03]	2.03	.049
GroupFluoxetine	31.44	[−31.88, 94.75]	0.97	.336
GroupStress	131.20	[67.89, 194.52]	4.06	< .001
**LDT Time spent in light, s Gauss distribution identity link**				
Intercept	154.07	[113.70, 194.43]	7.48	< .001
GroupFluoxetine	-8.07	[−65.15, 49.02]	-0.28	.783
GroupStress	-18.67	[−75.75, 38.42]	-0.64	.525
**LDT Number of light entries Gauss distribution identity link**				
Intercept	8.33	[5.51, 11.16]	5.78	< .001
GroupFluoxetine	-1.40	[−5.40, 2.60]	-0.69	.496
GroupStress	-0.27	[−4.27, 3.73]	-0.13	.897
**ST Distance to surface, cm Gamma distribution inverse link**				
Intercept	2.03	[1.78, 2.30]	15.23	< .001
GroupFluoxetine	0.10	[−0.27, 0.47]	0.51	.613
GroupStress	0.34	[−0.03, 0.71]	1.79	.080
**Dopamine, pg/mg inverted Gauss distribution log link**				
Intercept	5.52	[5.24, 5.91]	33.49	< .001
GroupFluoxetine	0.06	[-0.42, 0.55]	0.25	.803
GroupStress	0.21	[-0.28, 0.73]	0.86	.399
**DOPAC/dopamine ratio Gamma distribution inverted link**				
Intercept	9.29	[6.37, 12.99]	5.52	< .001
GroupFluoxetine	-0.81	[-5.39, 3.68]	-0.35	.726
GroupStress	-1.45	[-5.92, 2.85]	-0.66	.517
**DOPAC pg/mg Gamma distribution log link**				
Intercept	3.23	[2.87, 3.65]	16.32	< .001
GroupFluoxetine	0.20	[-0.36, 0.75]	0.70	.489
GroupStress	0.37	[-0.18, 0.92]	1.32	.198
**5-HIAA, pg/mg Gamma ditribution log link**				
Intercept	5.09	[4.78, 5.44]	30.38	< .001
GroupFluoxetine	-0.84	[-1.30, -0.37]	-3.52	.002
GroupStress	0.05	[-0.41, 0.52]	0.23	.823
**5-HIAA/serotonin inverted Gauss distribution log link**				
Intercept	-0.42	[-0.82, 0.26]	-1.66	.108

GroupFluoxetine	-0.89	[-1.62, -0.34]	-2.98	.006
GroupStress	-0.27	[-1.03, 0.41]	-0.82	.422
**Serotonin, pg/mg inverted Gauss distribution log link**				
Intercept	5.59	[5.34, 5.93]	37.89	< .001
GroupFluoxetine	0.03	[-0.40, 0.46]	0.14	.886
GroupStress	0.38	[-0.08, 0.87]	1.63	.114
**HVA/dopamine inverted Gauss distribution inverse link**				
Intercept	39.01	[21.75, 56.27]	4.43	< .001
GroupFluoxetine	8.82	[-16.94, 34.57]	0.67	.508
GroupStress	27.33	[-1.03, 55.70]	1.89	.070
**HVA, pg/mg inverted Gauss distribution log link**				
Intercept	1.73	[1.43, 2.18]	9.51	< .001
GroupFluoxetine	-0.09	[-0.62, 0.43]	-0.36	.724
GroupStress	-0.39	[-0.90, 0.07]	-1.66	.109
**Norepinephrine, pg/mg inverted Gauss log link**				
Intercept	6.93	[6.66, 7.29]	44.75	< .001
GroupFluoxetine	-0.01	[-0.45, 0.44]	-0.03	.980
GroupStress	0.21	[-0.26, 0.69]	0.89	.381

Supplementary Table S7. Results of Generalized Linear Model (GZLM) fits using week, group, their interaction effects and the involvement in behavioral battery as 'predictors' to compare stress and control group at weeks 1-5 of chronic unpredictable stress (CUS). The corrected Akaike information criterion (AIC) was used to choose the 'best fit' model among Gaussian distribution (identity link), Poisson distribution (with log link), Gamma distribution (inverse and log links) and Inverse Gaussian distribution (with inverse and log links) for levels of serotonin, dopamine, norepinephrine and their metabolites in the whole brain tissue using HPLC. behaviors assessed in the present study. Because no significant effects were observed for behavioral predictor in any models, it was excluded from further analyses, also see Supplementary Table S1 for details of GZLMs utilized in this study. Monoamine metabolites assessed here included 5-HIAA - 5-hydroxyindoleacetic acid, DOPAC - 3,4-dihydroxyphenylacetic acid, and HVA - homovanillic acid.

**Predictor**	b	**95% CI**	$t(170)$	p
**Dopamine pg/mg**				
Intercept	5.54	[5.31, 5.83]	42.22	< .001
Week2	-0.12	[−0.47, 0.22]	-0.71	.480
Week3	0.02	[−0.34, 0.38]	0.12	.902
Week4	-0.20	[−0.54, 0.14]	-1.17	.246
Week5	-0.03	[−0.38, 0.33]	-0.14	.887
GroupStress	-0.04	[−0.40, 0.31]	-0.23	.816
BehaviorN	0.00	[−0.15, 0.16]	0.06	.956
Week2 × GroupStress	0.22	[−0.27, 0.72]	0.89	.377
Week3 × GroupStress	0.22	[−0.29, 0.73]	0.84	.402
Week4 × GroupStress	-0.05	[−0.52, 0.42]	-0.22	.830
Week5 × GroupStress	0.25	[−0.25, 0.76]	0.99	.325
**DOPAC/dopamine ratio**				
Intercept	8.86	[5.21, 12.51]	4.69	< .001
Week2	-2.55	[−7.19, 2.09]	-1.08	.285
Week3	0.44	[−4.64, 5.52]	0.17	.866
Week4	-1.37	[−6.18, 3.44]	-0.56	.578
Week5	0.24	[−4.82, 5.30]	0.09	.927
GroupStress	0.79	[−4.34, 5.93]	0.30	.762
BehaviorN	0.33	[−1.89, 2.55]	0.30	.765
Week2 × GroupStress	-1.52	[−8.10, 5.07]	-0.45	.652
Week3 × GroupStress	4.76	[−3.03, 12.54]	1.20	.233
Week4 × GroupStress	0.20	[−6.79, 7.19]	0.06	.955
Week5 × GroupStress	-2.23	[−9.32, 4.86]	-0.62	.538
**DOPAC, pg/mg**				
Intercept	3.41	[3.04, 3.96]	15.04	< .001
Week2	0.10	[−0.56, 0.78]	0.32	.748
Week3	-0.02	[−0.67, 0.63]	-0.07	.944
Week4	-0.06	[−0.71, 0.57]	-0.21	.835
Week5	-0.10	[−0.75, 0.53]	-0.33	.746
GroupStress	-0.22	[−0.86, 0.38]	-0.77	.443
BehaviorN	-0.11	[−0.39, 0.18]	-0.80	.423
Week2 × GroupStress	0.37	[−0.51, 1.30]	0.85	.400
Week3 × GroupStress	-0.08	[−0.91, 0.76]	-0.19	.851
Week4 × GroupStress	0.01	[−0.81, 0.84]	0.03	.973
Week5 × GroupStress	0.58	[−0.28, 1.50]	1.35	.182
**5-HIAA pg/mg**				
Intercept	5.21	[4.92, 5.54]	32.49	< .001
Week2	-0.29	[−0.72, 0.13]	-1.35	.180
Week3	0.01	[−0.42, 0.43]	0.03	.974
Week4	-0.05	[−0.48, 0.37]	-0.25	.801
Week5	-0.17	[−0.59, 0.26]	-0.77	.444
GroupStress	-0.17	[−0.60, 0.26]	-0.78	.435
BehaviorN	0.08	[−0.11, 0.27]	0.82	.417
Week2 × GroupStress	0.64	[0.04, 1.25]	2.10	.038
Week3 × GroupStress	-0.20	[−0.80, 0.40]	-0.66	.513
Week4 × GroupStress	-0.42	[−1.03, 0.18]	-1.38	.172
Week5 × GroupStress	0.23	[−0.38, 0.83]	0.74	.462
**5-HIAA/serotonin ratio**				
Intercept	1.64	[1.05, 2.23]	5.38	< .001
Week2	0.30	[−0.55, 1.14]	0.69	.492
Week3	-0.09	[−0.89, 0.70]	-0.23	.816
Week4	-0.29	[−1.06, 0.47]	-0.76	.451

Week5	-0.04	[−0.84, 0.76]	-0.10	.923
GroupStress	-0.01	[−0.81, 0.79]	-0.03	.979
BehaviorN	-0.13	[−0.50, 0.24]	-0.70	.484
Week2 × GroupStress	-0.82	[−1.93, 0.29]	-1.44	.153
Week3 × GroupStress	1.44	[0.19, 2.68]	2.27	.026
Week4 × GroupStress	1.02	[−0.16, 2.20]	1.71	.091
Week5 × GroupStress	0.47	[−0.70, 1.64]	0.79	.433
**Serotonin, pg/mg**				
Intercept	5.75	[5.50, 6.06]	40.86	< .001
Week2	-0.09	[−0.47, 0.28]	-0.47	.638
Week3	-0.03	[−0.42, 0.34]	-0.18	.857
Week4	-0.10	[−0.47, 0.27]	-0.52	.602
Week5	-0.14	[−0.52, 0.22]	-0.78	.440
GroupStress	-0.11	[−0.49, 0.26]	-0.59	.557
BehaviorN	-0.03	[−0.20, 0.14]	-0.36	.721
Week2 × GroupStress	0.09	[−0.42, 0.61]	0.36	.722
Week3 × GroupStress	0.36	[−0.17, 0.91]	1.34	.185
Week4 × GroupStress	-0.01	[−0.52, 0.50]	-0.04	.970
Week5 × GroupStress	0.49	[−0.04, 1.04]	1.82	.073
**HVA/dopamine ratio**				
Intercept	-3.64	[−4.03, −3.01]	-14.77	< .001
Week2	-0.17	[−0.90, 0.52]	-0.53	.599
Week3	-0.44	[−1.14, 0.18]	-1.43	.157
Week4	0.07	[−0.67, 0.83]	0.19	.848
Week5	-0.04	[−0.76, 0.68]	-0.11	.913
GroupStress	-0.32	[−1.03, 0.31]	-1.02	.309
BehaviorN	0.04	[−0.25, 0.32]	0.27	.791
Week2 × GroupStress	0.66	[−0.26, 1.66]	1.44	.153
Week3 × GroupStress	0.19	[−0.64, 1.04]	0.46	.649
Week4 × GroupStress	-0.15	[−1.08, 0.76]	-0.34	.735
Week5 × GroupStress	-0.21	[−1.10, 0.66]	-0.50	.619
**HVA, pg/mg**				
Intercept	0.14	[0.07, 0.22]	3.65	< .001
Week2	0.04	[−0.07, 0.15]	0.77	.446
Week3	0.08	[−0.04, 0.20]	1.37	.173
Week4	0.03	[−0.08, 0.13]	0.49	.627
Week5	0.03	[−0.08, 0.14]	0.54	.592
GroupStress	0.08	[−0.03, 0.20]	1.39	.167
BehaviorN	0.01	[−0.05, 0.06]	0.30	.766
Week2 × GroupStress	-0.13	[−0.29, 0.03]	-1.57	.120
Week3 × GroupStress	-0.10	[−0.27, 0.08]	-1.09	.279
Week4 × GroupStress	0.04	[−0.13, 0.22]	0.47	.636
Week5 × GroupStress	0.00	[−0.17, 0.17]	0.02	.981
**Norepinephrine, pg/mg**				
Intercept	937.51	[690.28, 1,184.75]	7.43	< .001
Week2	-163.89	[−497.27, 169.48]	-0.96	.338
Week3	-106.53	[−439.91, 226.84]	-0.63	.533
Week4	-197.99	[−531.37, 135.38]	-1.16	.248
Week5	105.58	[−227.80, 438.95]	0.62	.536
GroupStress	-78.08	[−411.45, 255.30]	-0.46	.647
BehaviorN	-48.15	[−197.24, 100.94]	-0.63	.528
Week2 × GroupStress	254.60	[−216.86, 726.06]	1.06	.293
Week3 × GroupStress	463.23	[−8.24, 934.69]	1.93	.057
Week4 × GroupStress	100.21	[−371.25, 571.68]	0.42	.678
Week5 × GroupStress	310.81	[−160.65, 782.28]	1.29	.200

Supplementary Table S8. Summary of statistical analyses for all differentially expressed genes in stress vs. control, fluoxetine vs. control or fluoxetine vs. stress groups, on the Negative Binomial (Gamma-Poisson) distribution performed by estimation of size factors, estimation of dispersion and negative binomial generalized linear models and Wald statistics using the DESeq function. P value and false discovery rate were set at 0.05. Genes in this table are listed as sorted by their p-adjusted value.

Ensembl	external_gene_name	baseMean	log2FoldChange	lfcSE	Stat	pvalue	padj
**Stress vs. Control**							
ENSDARG00000093998	si:ch73-7i4.2	164,8442	5,686818	0,620534	9,164391	4,98E-20	1,21E-15
ENSDARG00000102639	si:dkey-29d5.2	41,03763	6,214117	0,924045	6,724906	1,76E-11	2,14E-07
ENSDARG00000079402	tapbp.1	144,5193	-1,78864	0,284994	-6,27605	3,47E-10	2,82E-06
ENSDARG00000094554	si:ch73-7i4.1	37,6995	4,12461	0,750041	5,499177	3,82E-08	0,000232
ENSDARG00000076586	csf2rb	50,70728	-1,85704	0,341433	-5,43895	5,36E-08	0,000248
ENSDARG00000090890	cmklr1	55,55607	-1,7606	0,325129	-5,41508	6,13E-08	0,000248
ENSDARG00000100302	si:dkey-237g15.2	108,2872	-3,08287	0,601631	-5,12419	2,99E-07	0,000891
ENSDARG00000102835	CABZ01021435.1	26,96331	-1,9449	0,380925	-5,10574	3,3E-07	0,000891
ENSDARG00000079034	zpax4	15,31746	2,711004	0,529375	5,121144	3,04E-07	0,000891
ENSDARG00000090637	myh6	13,27576	-5,20513	1,062621	-4,89839	9,66E-07	0,002138
ENSDARG00000069839	CU571382.1	29,63909	-3,33594	0,680015	-4,90569	9,31E-07	0,002138
ENSDARG00000053973	fetub	6,957216	3,670704	0,752827	4,875896	1,08E-06	0,002197
ENSDARG00000086374	isg15	261,0382	-3,83891	0,793292	-4,83921	1,3E-06	0,002263
ENSDARG00000104120	LO018260.1	67,36825	-1,21762	0,252712	-4,8182	1,45E-06	0,002263
ENSDARG00000077249	gmip	54,58877	-1,06096	0,221001	-4,80072	1,58E-06	0,002263
ENSDARG00000002988	tnnt2d	40,15867	2,050141	0,426229	4,809951	1,51E-06	0,002263
ENSDARG00000026979	krt1-c5	299,3362	7,251248	1,510097	4,801841	1,57E-06	0,002263
ENSDARG00000074136	slc47a2.2	13,28693	-4,82162	1,01405	-4,75481	1,99E-06	0,002306
ENSDARG00000014427	mxe	68,81475	-4,23896	0,891577	-4,75445	1,99E-06	0,002306
ENSDARG00000094719	CR318588.3	1227,035	-4,16004	0,869977	-4,78178	1,74E-06	0,002306
ENSDARG00000078389	ifi46	158,1358	-2,49557	0,522744	-4,77398	1,81E-06	0,002306
ENSDARG00000059294	marco	174,3955	-2,23722	0,476887	-4,6913	2,71E-06	0,002872
ENSDARG00000079997	si:zfos-223e1.2	33,87397	1,598868	0,3406	4,694269	2,68E-06	0,002872
ENSDARG00000087102	si:ch1073-164k15.3	54,19928	-4,9841	1,067979	-4,66685	3,06E-06	0,003101
ENSDARG00000055186	ccr9a	44,12408	-2,80143	0,603577	-4,64138	3,46E-06	0,003369
ENSDARG00000094386	adgrg4b	49,28134	8,419412	1,829425	4,602218	4,18E-06	0,003913
ENSDARG00000088251	lgals17	195,6001	-4,19997	0,922056	-4,55501	5,24E-06	0,004605
ENSDARG00000039490	pitpnaa	65,80556	-1,27998	0,281611	-4,54521	5,49E-06	0,004605
ENSDARG00000100732	si:dkeyp-93h6.1	24,14235	1,992828	0,437833	4,551574	5,32E-06	0,004605
ENSDARG00000029720	si:dkeyp-9d4.2	118,4261	-2,75882	0,609126	-4,52914	5,92E-06	0,004758
ENSDARG00000010946	cbsb	1229,743	-1,11274	0,24621	-4,51949	6,2E-06	0,004758
ENSDARG00000101585	tecra	76,98249	6,34775	1,405135	4,517538	6,26E-06	0,004758
ENSDARG00000010317	gpr183a	18,51935	-1,57612	0,351277	-4,48682	7,23E-06	0,005175
ENSDARG00000067990	myhz1.1	64,67868	2,700507	0,601157	4,492184	7,05E-06	0,005175
ENSDARG00000010619	themis	14,19646	-1,81447	0,40819	-4,44516	8,78E-06	0,005937
ENSDARG00000114894	znf1026	19,7091	-1,38193	0,310862	-4,44548	8,77E-06	0,005937
ENSDARG00000090600	si:ch211-213a13.1	323,8362	-3,22941	0,728271	-4,43435	9,24E-06	0,006074
ENSDARG00000117491	CU855688.1	14,46722	1,922018	0,434193	4,426639	9,57E-06	0,00613

ENSDARG00000092233	vtg1	176,6857	2,944982	0,6664	4,419243	9,9E-06	0,006181
ENSDARG00000028731	stat4	34,94947	-1,58575	0,36035	-4,40058	1,08E-05	0,006568
ENSDARG00000007467	ifit10	18,52404	-4,94187	1,137183	-4,34571	1,39E-05	0,007947
ENSDARG00000069961	il21r.1	63,55553	-1,29313	0,297719	-4,34348	1,4E-05	0,007947
ENSDARG00000032885	si:ch211-226m7.4	33,89089	1,547943	0,356405	4,343211	1,4E-05	0,007947
ENSDARG00000093214	si:ch211-284e13.9	23,50924	-1,66773	0,388949	-4,2878	1,8E-05	0,009981
ENSDARG00000095673	si:dkey-79f11.8	30,39832	-6,4794	1,529671	-4,23581	2,28E-05	0,012115
ENSDARG00000088831	si:dkey-81j8.6	22,07982	-1,99191	0,470394	-4,23455	2,29E-05	0,012115
ENSDARG00000101014	slco1f4	4,985412	5,932084	1,402738	4,228934	2,35E-05	0,012158
ENSDARG00000105266	si:dkey-16p6.1	14,8571	-2,59149	0,615033	-4,21357	2,51E-05	0,012737
ENSDARG00000075346	tmem129	255,4415	0,505023	0,119985	4,209047	2,56E-05	0,012737
ENSDARG00000074283	inpp5d	116,6038	-1,11091	0,264307	-4,2031	2,63E-05	0,012815
ENSDARG00000095026	si:dkey-58f10.14	10,72728	-4,15513	0,994521	-4,17802	2,94E-05	0,013357
ENSDARG00000101728	si:dkeyp-80d11.12	9,188694	-3,03997	0,729765	-4,16568	3,1E-05	0,013357
ENSDARG00000071543	si:dkey-42i9.7	28,76336	-2,9514	0,706858	-4,17538	2,97E-05	0,013357
ENSDARG00000043475	si:ch211-122h15.4	113,3422	-1,67301	0,400194	-4,1805	2,91E-05	0,013357
ENSDARG00000071437	ptprc	343,6064	-1,11075	0,266757	-4,1639	3,13E-05	0,013357
ENSDARG00000062049	arhgap45b	99,35305	-0,92879	0,222678	-4,17098	3,03E-05	0,013357
ENSDARG00000055809	vtg2	19,39724	5,828072	1,392504	4,185318	2,85E-05	0,013357
ENSDARG00000056791	pde6ga	21,88924	-3,64709	0,878431	-4,15182	3,3E-05	0,01384
ENSDARG00000074052	si:ch211-281g13.5	61,93331	-3,65391	0,884609	-4,13054	3,62E-05	0,014438
ENSDARG00000099949	FP236551.1	48,90707	-1,8141	0,439081	-4,13158	3,6E-05	0,014438
ENSDARG00000032631	ltb4r	39,66947	-1,54528	0,373787	-4,13413	3,56E-05	0,014438
ENSDARG00000089463	dhx58	63,537	-3,29948	0,802559	-4,1112	3,94E-05	0,01545
ENSDARG00000041379	si:ch211-245j22.3	53,2759	-5,22288	1,276876	-4,09036	4,31E-05	0,016638
ENSDARG00000111309	AL935126.2	37,11771	7,400761	1,812357	4,083502	4,44E-05	0,016869
ENSDARG00000052288	zmp:0000000634	80,56334	-5,23374	1,284563	-4,07434	4,61E-05	0,017015
ENSDARG00000077090	si:ch211-127b11.1	16,06137	5,489022	1,34639	4,076845	4,57E-05	0,017015
ENSDARG00000004459	unc119.2	45,52201	-4,57894	1,130001	-4,05215	5,07E-05	0,017016
ENSDARG00000054418	ssuh2.2	16,49514	-2,77854	0,685146	-4,0554	5E-05	0,017016
ENSDARG00000102525	lck	17,0159	-1,98235	0,488948	-4,05431	5,03E-05	0,017016
ENSDARG00000019728	bmpr1aa	439,3845	0,627031	0,154914	4,047613	5,17E-05	0,017016
ENSDARG00000058256	draxin	1407,05	0,665238	0,164239	4,050415	5,11E-05	0,017016
ENSDARG00000094668	si:ch211-51a19.5	85,66699	2,73115	0,671709	4,065971	4,78E-05	0,017016
ENSDARG00000098315	cyp1a	20,05002	3,458075	0,854267	4,048005	5,17E-05	0,017016
ENSDARG00000052165	si:ch211-236k19.2	75,20497	5,488341	1,349347	4,067406	4,75E-05	0,017016
ENSDARG00000074656	ctss2.1	46,2542	-1,63224	0,403898	-4,04123	5,32E-05	0,017253
ENSDARG00000015176	ror1	39,3239	-1,00205	0,248429	-4,03355	5,49E-05	0,017592
ENSDARG00000075389	si:ch211-281g13.4	30,43102	-3,7568	0,933473	-4,02454	5,71E-05	0,018042
ENSDARG00000007018	ms4a17a.6	23,82854	-1,55099	0,386569	-4,0122	6,02E-05	0,018768
ENSDARG00000035798	gngt1	231,2607	-4,58871	1,148379	-3,99582	6,45E-05	0,019415
ENSDARG00000004953	mxb	68,51614	-3,91733	0,979392	-3,99976	6,34E-05	0,019415
ENSDARG00000076182	stat1b	200,0531	-3,00857	0,754124	-3,98949	6,62E-05	0,019415
ENSDARG00000038095	socs1a	60,44625	-1,72096	0,431249	-3,99065	6,59E-05	0,019415
ENSDARG00000088366	zgc:77938	193,9505	1,411368	0,35339	3,993799	6,5E-05	0,019415
ENSDARG00000075757	gig2e	11,43792	-2,30678	0,578929	-3,98457	6,76E-05	0,019586

ENSDARG00000074546	si:ch211-213a13.2	207,1771	-4,26256	1,071408	-3,97847	6,94E-05	0,019801
ENSDARG00000104045	tlr22	9,832313	-2,26098	0,569042	-3,97331	7,09E-05	0,019801
ENSDARG00000015752	zap70	32,19613	-1,42027	0,357668	-3,9709	7,16E-05	0,019801
ENSDARG00000037408	tbc1d10c	41,53559	-1,194	0,300553	-3,97268	7,11E-05	0,019801
ENSDARG00000052396	pkz	35,78193	-2,68548	0,677851	-3,96175	7,44E-05	0,020344
ENSDARG00000010252	jak3	44,89497	-1,21599	0,307974	-3,94835	7,87E-05	0,021278
ENSDARG00000040942	pnp6	782,434	0,691913	0,175374	3,945363	7,97E-05	0,021308
ENSDARG00000092259	si:dkey-211g8.5	3,67091	-5,58819	1,422267	-3,92907	8,53E-05	0,022002
ENSDARG00000075072	rpap1	258,9355	-0,62954	0,160114	-3,93184	8,43E-05	0,022002
ENSDARG00000014196	myl1	22,47509	2,590033	0,659487	3,927348	8,59E-05	0,022002
ENSDARG00000045822	tnnt2e	97,31019	2,923894	0,744396	3,927875	8,57E-05	0,022002
ENSDARG00000101071	si:dkeyp-80d11.14	9,692705	-1,69541	0,43224	-3,92239	8,77E-05	0,022226
ENSDARG00000093748	si:ch211-217k17.11	74,89433	-3,07385	0,786411	-3,90871	9,28E-05	0,023042
ENSDARG00000037358	polr3e	639,91	0,740317	0,189372	3,909328	9,26E-05	0,023042
ENSDARG00000105364	si:ch73-223f5.1	249,9613	-2,25547	0,578322	-3,90002	9,62E-05	0,023602
ENSDARG00000055730	trim35-29	26,2314	2,224615	0,570704	3,89802	9,7E-05	0,023602
ENSDARG00000076158	zgc:165583	14,77398	-1,73106	0,444598	-3,89354	9,88E-05	0,023804
ENSDARG00000098189	si:dkey-16p6.1	21,01967	-2,0213	0,520049	-3,88674	0,000102	0,024005
ENSDARG00000075504	si:dkey-40c23.2	39,2451	-1,77002	0,455386	-3,88687	0,000102	0,024005
ENSDARG00000117508	CU459056.2	31,31827	-2,81318	0,726197	-3,87385	0,000107	0,025048
ENSDARG00000104450	CR855996.2	18,79987	2,993049	0,773054	3,871719	0,000108	0,025048
ENSDARG00000092191	CR318588.1	1186,549	-3,8714	1,002492	-3,86178	0,000113	0,025602
ENSDARG00000040582	C18H3orf33	96,04662	0,956905	0,247762	3,862198	0,000112	0,025602
ENSDARG00000058774	CABZ01053221.1	6,751038	-2,13749	0,554449	-3,85516	0,000116	0,02606
ENSDARG00000077130	bcl10	19,27022	-1,44581	0,375445	-3,85093	0,000118	0,026272
ENSDARG00000025428	socs3a	319,8547	-2,28392	0,593862	-3,84587	0,00012	0,026576
ENSDARG00000061177	mov10b.1	67,60348	-2,23373	0,582393	-3,83543	0,000125	0,027056
ENSDARG00000044235	BX005421.1	11,77669	-1,87999	0,490235	-3,83487	0,000126	0,027056
ENSDARG00000022183	gsto1	561,16	0,791405	0,206245	3,837212	0,000124	0,027056
ENSDARG00000070028	tmem41aa	294,3783	-0,52064	0,136132	-3,82454	0,000131	0,027969
ENSDARG00000086391	cald1b	29,47612	1,741375	0,455576	3,822362	0,000132	0,027971
ENSDARG00000013855	slc12a3	25,84152	2,833511	0,741834	3,819602	0,000134	0,028042
ENSDARG00000100968	si:ch211-1a19.3	127,6878	-1,3544	0,355749	-3,80717	0,000141	0,029237
ENSDARG00000097559	cyp8b3	23,46184	-3,1911	0,83928	-3,80219	0,000143	0,02958
ENSDARG00000093753	BX004774.2	193,7659	-3,35418	0,884297	-3,79305	0,000149	0,030432
ENSDARG00000013655	tpd52l2b	1755,256	0,396047	0,104678	3,783498	0,000155	0,031102
ENSDARG00000099860	pkmb	19,15483	1,985092	0,52447	3,784949	0,000154	0,031102
ENSDARG00000032584	thnsl2	58,5869	1,312257	0,347211	3,77942	0,000157	0,031356
ENSDARG00000112454	FO744833.2	250,7418	-1,06081	0,281449	-3,7691	0,000164	0,032415
ENSDARG00000039243	zgc:152791	379,5849	-4,08968	1,08629	-3,76482	0,000167	0,032449
ENSDARG00000107526	smkr1	91,23716	1,22454	0,325166	3,765891	0,000166	0,032449
ENSDARG00000038288	cd151	53,99511	1,22684	0,326066	3,762555	0,000168	0,032484
ENSDARG00000101169	grap2b	69,45649	-1,34499	0,357704	-3,76006	0,00017	0,032551
ENSDARG00000068939	xaf1	80,99448	-2,10235	0,5609	-3,74818	0,000178	0,033866
ENSDARG00000043483	otx5	21,80288	-4,75563	1,270297	-3,74372	0,000181	0,033915
ENSDARG00000078731	si:dkey-85k7.12	46,23873	-3,24038	0,865637	-3,74335	0,000182	0,033915

ENSDARG00000100900	si:ch211-183d5.2	90,99566	-2,64398	0,70701	-3,73967	0,000184	0,033915
ENSDARG00000092653	si:dkey-1h24.6	10,56826	-1,46668	0,391992	-3,7416	0,000183	0,033915
ENSDARG00000002682	tbcela	1466,567	0,437677	0,117083	3,738187	0,000185	0,033915
ENSDARG00000092126	vtg5	23,70013	3,134181	0,842453	3,720305	0,000199	0,036138
ENSDARG00000101272	si:dkey-29j8.1	6,759197	-1,94503	0,523401	-3,71615	0,000202	0,036465
ENSDARG00000055159	cyp27a1.4	191,0922	0,818813	0,220705	3,709983	0,000207	0,036863
ENSDARG00000053323	zgc:112285	19,59111	1,085756	0,292827	3,707837	0,000209	0,036863
ENSDARG00000097580	BX663519.1	55,41507	2,045346	0,55146	3,708969	0,000208	0,036863
ENSDARG00000089185	si:ch73-264i18.2	2,574693	5,23719	1,41348	3,705175	0,000211	0,036985
ENSDARG00000032932	cnksr1	19,13048	-1,15229	0,311167	-3,70313	0,000213	0,037018
ENSDARG00000054610	coro1a	158,7542	-1,13353	0,306657	-3,69639	0,000219	0,037478
ENSDARG00000034063	unm_sa911	124,8442	-1,01276	0,273941	-3,697	0,000218	0,037478
ENSDARG00000090730	cfbl	23,55705	-1,94248	0,527028	-3,68572	0,000228	0,038276
ENSDARG00000089227	si:ch211-91p5.3	148,7863	-1,22865	0,333224	-3,68714	0,000227	0,038276
ENSDARG00000004954	grna	435,3453	-0,97673	0,264828	-3,68817	0,000226	0,038276
ENSDARG00000008592	mtmr8	211,84	-1,80008	0,489257	-3,67922	0,000234	0,038995
ENSDARG00000074501	tnip2	14,95865	-1,8318	0,498515	-3,67452	0,000238	0,039451
ENSDARG00000112831	znf1116	5,616213	-3,68212	1,007437	-3,65494	0,000257	0,040309
ENSDARG00000034095	si:dkey-59l11.10	4,273366	-3,45926	0,947606	-3,65053	0,000262	0,040309
ENSDARG00000113386	LO018432.1	13,70658	-3,40887	0,933373	-3,6522	0,00026	0,040309
ENSDARG00000093254	znf569l	22,27115	-1,74352	0,476823	-3,65653	0,000256	0,040309
ENSDARG00000002165	psme1	196,5018	-1,24669	0,340793	-3,6582	0,000254	0,040309
ENSDARG00000094451	cfp	291,775	-1,13416	0,309322	-3,66658	0,000246	0,040309
ENSDARG00000094854	ms4a17a.9	23,93016	-1,12734	0,308705	-3,65183	0,00026	0,040309
ENSDARG00000071558	fblim1	86,34791	0,791259	0,216127	3,661085	0,000251	0,040309
ENSDARG00000096045	si:ch73-263o4.4	21,07568	1,864121	0,510054	3,654755	0,000257	0,040309
ENSDARG00000087641	cdh26.2	27,49433	2,062629	0,563345	3,661399	0,000251	0,040309
ENSDARG00000045592	tnni2a.1	41,53237	4,136382	1,131927	3,654282	0,000258	0,040309
ENSDARG00000092159	CR788249.1	20,92327	-4,21296	1,157248	-3,6405	0,000272	0,041537
ENSDARG00000039512	ap1m3	51,50422	-1,22567	0,336763	-3,63957	0,000273	0,041537
ENSDARG00000096355	ighv1-4	116,5287	-4,07637	1,120754	-3,63717	0,000276	0,041666
ENSDARG00000008306	rdh5	41,4861	-3,83153	1,056286	-3,62736	0,000286	0,04275
ENSDARG00000100434	znf1091	28,42767	-3,03531	0,83643	-3,62889	0,000285	0,04275
ENSDARG00000052223	rcvrna	24,89004	-4,98773	1,38359	-3,60492	0,000312	0,043175
ENSDARG00000100568	si:ch211-209j12.3	5,64605	-3,59469	0,996871	-3,60598	0,000311	0,043175
ENSDARG00000002696	gnb3b	50,1038	-2,72007	0,754059	-3,60724	0,000309	0,043175
ENSDARG00000004952	rsad2	151,1438	-2,29699	0,636882	-3,60662	0,00031	0,043175
ENSDARG00000098515	si:dkey-16p6.1	54,17549	-1,69489	0,469968	-3,60639	0,00031	0,043175
ENSDARG00000100669	si:dkey-61p9.9	18,42742	-1,47296	0,408158	-3,60879	0,000308	0,043175
ENSDARG00000114991	tmem14cb	32,63878	-1,22506	0,338416	-3,61999	0,000295	0,043175
ENSDARG00000069774	flot2b	1183,238	0,363745	0,100632	3,614625	0,000301	0,043175
ENSDARG00000015482	zgc:113518	197,057	0,514276	0,142076	3,619716	0,000295	0,043175
ENSDARG00000075599	cdc25d	51,14493	0,687367	0,190245	3,613054	0,000303	0,043175
ENSDARG00000094901	abcc6b.2	17,71361	1,093126	0,301822	3,621754	0,000293	0,043175
ENSDARG00000019128	tpm4b	69,61131	1,403176	0,38894	3,607694	0,000309	0,043175
ENSDARG00000095024	si:dkeyp-68b7.10	5,475087	2,323612	0,642833	3,614645	0,000301	0,043175

ENSDARG00000044612	c1qb	41,35639	-1,48744	0,412887	-3,60255	0,000315	0,043325
ENSDARG00000101309	si:ch73-100l22.3	227,2183	0,752427	0,209258	3,595699	0,000324	0,044232
ENSDARG00000101675	zgc:123107	60,63829	-1,22681	0,341577	-3,5916	0,000329	0,044433
ENSDARG00000020610	tnnt2a	13,70478	2,009186	0,559411	3,59161	0,000329	0,044433
ENSDARG00000005713	ethe1	180,6208	0,715788	0,200044	3,578154	0,000346	0,046016
ENSDARG00000098046	FP017161.1	196,3582	0,871499	0,243536	3,578517	0,000346	0,046016
ENSDARG00000098811	LO018021.1	38,82198	0,879947	0,245746	3,580713	0,000343	0,046016
ENSDARG00000054340	sla1a	12,8555	-1,84624	0,517082	-3,5705	0,000356	0,047124
ENSDARG00000078618	inpp5kb	54,3152	-2,24734	0,629697	-3,56892	0,000358	0,047153
ENSDARG00000089930	iqce	451,2363	0,846778	0,237392	3,567004	0,000361	0,047244
ENSDARG00000044199	gnat1	271,0448	-4,22288	1,186217	-3,55995	0,000371	0,048014
ENSDARG00000103551	rnf213b	78,05034	-2,76741	0,777137	-3,56103	0,000369	0,048014
ENSDARG00000078130	CR391986.1	68,27406	0,555449	0,156091	3,558495	0,000373	0,048026
ENSDARG00000091627	si:dkey-271j15.3	18,75689	-2,85533	0,802833	-3,55657	0,000376	0,048046
ENSDARG00000100420	tpcn2	38,46233	-2,2763	0,640198	-3,55562	0,000377	0,048046
ENSDARG00000103720	zgc:162730	428,5616	-1,05731	0,297686	-3,55178	0,000383	0,048499
ENSDARG00000034160	slc4a1ap	852,5459	0,356273	0,100466	3,546204	0,000391	0,049026
ENSDARG00000038941	ppp1r1c	914,9612	0,947675	0,26721	3,546548	0,00039	0,049026
ENSDARG00000090977	ifit12	8,278224	-2,10441	0,593758	-3,54422	0,000394	0,049144
ENSDARG00000086848	atad3	94,54343	-0,72335	0,204181	-3,5427	0,000396	0,049174
**Fluoxetine vs. Control**							
ENSDARG00000093998	si:ch73-7i4.2	164,8442	5,23191	0,601678	8,695536	3,45E-18	7,63E-14
ENSDARG00000075757	gig2e	11,43792	-3,85335	0,635052	-6,06777	1,3E-09	1,43E-05
ENSDARG00000102639	si:dkey-29d5.2	41,03763	5,294999	0,903836	5,858365	4,67E-09	3,44E-05
ENSDARG00000069839	CU571382.1	29,63909	-3,57225	0,656588	-5,44063	5,31E-08	0,000276
ENSDARG00000078389	ifi46	158,1358	-2,7274	0,50396	-5,41194	6,23E-08	0,000276
ENSDARG00000087102	si:ch1073-164k15.3	54,19928	-5,37584	1,034098	-5,19858	2,01E-07	0,00074
ENSDARG00000026979	krt1-c5	299,3362	7,266663	1,457883	4,984394	6,22E-07	0,001963
ENSDARG00000090600	si:ch211-213a13.1	323,8362	-3,46337	0,701894	-4,93433	8,04E-07	0,002101
ENSDARG00000029720	si:dkeyp-9d4.2	118,4261	-2,8883	0,586773	-4,92235	8,55E-07	0,002101
ENSDARG00000092919	zp3c	4,913544	-5,64865	1,157449	-4,88026	1,06E-06	0,002342
ENSDARG00000088251	lgals17	195,6001	-4,28668	0,888117	-4,8267	1,39E-06	0,00279
ENSDARG00000074052	si:ch211-281g13.5	61,93331	-3,9522	0,853709	-4,62945	3,67E-06	0,00548
ENSDARG00000094719	CR318588.3	1227,035	-3,8692	0,838033	-4,617	3,89E-06	0,00548
ENSDARG00000092653	si:dkey-1h24.6	10,56826	-1,78926	0,384799	-4,64985	3,32E-06	0,00548
ENSDARG00000074466	gdpd3a	29,9061	1,20471	0,261146	4,613172	3,97E-06	0,00548
ENSDARG00000111309	AL935126.2	37,11771	8,144139	1,759393	4,628947	3,68E-06	0,00548
ENSDARG00000007018	ms4a17a.6	23,82854	-1,67663	0,371835	-4,50908	6,51E-06	0,00805
ENSDARG00000010252	jak3	44,89497	-1,33266	0,296398	-4,49619	6,92E-06	0,00805
ENSDARG00000052165	si:ch211-236k19.2	75,20497	5,87199	1,304001	4,503057	6,7E-06	0,00805
ENSDARG00000019601	col12a1b	137,9121	-3,57917	0,814411	-4,3948	1,11E-05	0,011672
ENSDARG00000076158	zgc:165583	14,77398	-1,88427	0,428078	-4,40171	1,07E-05	0,011672
ENSDARG00000095309	si:dkey-79f11.9	36,29543	-4,0224	0,920755	-4,36859	1,25E-05	0,01202
ENSDARG00000087641	cdh26.2	27,49433	2,372439	0,542722	4,371371	1,23E-05	0,01202
ENSDARG00000008592	mtmr8	211,84	-2,03965	0,471631	-4,32466	1,53E-05	0,012831
ENSDARG00000012881	slc4a1a	147,5154	-1,46805	0,339068	-4,32966	1,49E-05	0,012831

ENSDARG00000078193	si:ch211-67e16.3	11,84568	-1,37999	0,317853	-4,34161	1,41E-05	0,012831
ENSDARG00000094554	si:ch73-7i4.1	37,6995	3,148321	0,728938	4,319054	1,57E-05	0,012831
ENSDARG00000095026	si:dkey-58f10.14	10,72728	-4,04741	0,938916	-4,31072	1,63E-05	0,012848
ENSDARG00000024032	coch	283,3135	-3,06525	0,713951	-4,29337	1,76E-05	0,012969
ENSDARG00000079402	tapbp.1	144,5193	-1,1655	0,271111	-4,299	1,72E-05	0,012969
ENSDARG00000086374	isg15	261,0382	-3,21871	0,762464	-4,22146	2,43E-05	0,016892
ENSDARG00000074656	ctss2.1	46,2542	-1,63579	0,387645	-4,21982	2,45E-05	0,016892
ENSDARG00000090267	CU062628.1	16,30255	2,569695	0,610233	4,211005	2,54E-05	0,017032
ENSDARG00000034473	ttyh3a	1828,15	-0,34355	0,082096	-4,18477	2,85E-05	0,018561
ENSDARG00000105364	si:ch73-223f5.1	249,9613	-2,32072	0,557109	-4,16566	3,1E-05	0,01961
ENSDARG00000022183	gsto1	561,16	0,825369	0,198708	4,153682	3,27E-05	0,020092
ENSDARG00000040282	zgc:92590	58,28274	3,142379	0,764166	4,112165	3,92E-05	0,02342
ENSDARG00000102835	CABZ01021435.1	26,96331	-1,43292	0,353846	-4,04957	5,13E-05	0,029853
ENSDARG00000039243	zgc:152791	379,5849	-4,19111	1,046639	-4,00435	6,22E-05	0,034371
ENSDARG00000076586	csf2rb	50,70728	-1,28586	0,320894	-4,00713	6,15E-05	0,034371
ENSDARG00000089227	si:ch211-91p5.3	148,7863	-1,27266	0,320772	-3,96749	7,26E-05	0,039165
ENSDARG00000010729	CABZ01073795.1	515,0924	-3,24934	0,832737	-3,902	9,54E-05	0,04894
ENSDARG00000076182	stat1b	200,0531	-2,82559	0,725852	-3,89279	9,91E-05	0,04894
ENSDARG00000070132	aste1a	46,78711	-2,44764	0,63025	-3,8836	0,000103	0,04894
ENSDARG00000010619	themis	14,19646	-1,45562	0,375914	-3,87223	0,000108	0,04894
ENSDARG00000040942	pnp6	782,434	0,657659	0,169004	3,891394	9,97E-05	0,04894
ENSDARG00000092062	BX511123.2	74,96263	0,881983	0,227854	3,87082	0,000108	0,04894
ENSDARG00000056248	si:dkey-183i3.5	63,86707	2,962784	0,764435	3,875781	0,000106	0,04894
ENSDARG00000101585	tecra	76,98249	5,307561	1,359124	3,905133	9,42E-05	0,04894
**Fluoxetine vs. Stress**							
ENSDARG00000100302	si:dkey-237g15.2	108,2872	2,90793	0,581199	5,003329	5,63E-07	0,013086
ENSDARG00000100434	znf1091	28,42767	3,918893	0,807863	4,850939	1,23E-06	0,014269
ENSDARG00000094386	adgrg4b	49,28134	-7,56984	1,691682	-4,47474	7,65E-06	0,028179
ENSDARG00000102558	pde6ha	146,3818	2,761691	0,621272	4,445218	8,78E-06	0,028179
ENSDARG00000002696	gnb3b	50,1038	3,23506	0,727714	4,445509	8,77E-06	0,028179
ENSDARG00000056511	arr3a	96,37119	3,967655	0,888263	4,466758	7,94E-06	0,028179
ENSDARG00000035798	gngt1	231,2607	4,899159	1,107504	4,423605	9,71E-06	0,028179
ENSDARG00000104919	si:ch211-153b23.3	15,75144	6,587543	1,444016	4,561959	5,07E-06	0,028179
ENSDARG00000052057	pcolceb	37,76886	-3,10023	0,725539	-4,27301	1,93E-05	0,043741
ENSDARG00000059163	rbp3	153,2547	3,47854	0,809138	4,299067	1,72E-05	0,043741
ENSDARG00000012610	saga	87,58627	5,196321	1,220649	4,257013	2,07E-05	0,043741
ENSDARG00000099860	pkmb	19,15483	-2,12218	0,509276	-4,16705	3,09E-05	0,049546
ENSDARG00000057648	dnttip2	254,251	0,536119	0,129562	4,137921	3,5E-05	0,049546
ENSDARG00000095801	BX649485.2	68,99263	2,90827	0,702904	4,137506	3,51E-05	0,049546
ENSDARG00000008306	rdh5	41,4861	4,202817	1,020871	4,116895	3,84E-05	0,049546
ENSDARG00000041379	si:ch211-245j22.3	53,2759	5,096952	1,235552	4,125244	3,7E-05	0,049546
ENSDARG00000043483	otx5	21,80288	5,151354	1,233783	4,175251	2,98E-05	0,049546
ENSDARG00000105096	si:ch1073-67j19.1	13,8963	5,255277	1,261485	4,165945	3,1E-05	0,049546

Supplementary Table S9. Statistical data for pathways enriched in stress vs. control, fluoxetine vs. control or fluoxetine vs. stress groups as in Supplementary Table S1. The KEGG- and GO-pathways enrichment analysis was performed on normalized and log2-transformed counts by general applicable gene set enrichment for pathway analysis (GAGE) package[4], using two-tailed t-test for group comparison of differential expression of gene sets. False discovery rate cutoffs were set at 0.05 for KEGG pathways and 0.01 for GO pathways. The p value for GO pathways was reduced to 0.01 because it has more pathways than KEGG (~40 000 vs. ~500), and 0.05 FDR for GO already results in the large amount of significantly altered pathways, hence complicating our intended focus here on the most significant (e.g., most strongly affected) pathways instead. p.geomean – geometric mean of the individual p-values from multiple single array based gene set tests, stat.mean - mean of the individual statistics from multiple single array based gene set tests.

**Pathway**	**p.geomean**	**stat.mean**	**p.val**	**q.val**	**set.size**
Stress vs. Control upregulated					
GO					
GO:0043292 contractile fiber	5,7E-11	6,756234	5,7E-11	9,17E-08	134
GO:0030016 myofibril	6,37E-11	6,73993	6,37E-11	9,17E-08	133
GO:0044449 contractile fiber part	7,13E-11	6,723342	7,13E-11	9,17E-08	132
GO:0030017 sarcomere	7,97E-11	6,707025	7,97E-11	9,17E-08	131
GO:0005865 striated muscle thin filament	3,3E-10	6,94235	3,3E-10	2,53E-07	54
GO:0036379 myofilament	3,3E-10	6,94235	3,3E-10	2,53E-07	54
GO:0003735 structural constituent of ribosome	6,6E-10	6,388184	6,6E-10	4,34E-07	152
GO:0099080 supramolecular complex	1,24E-09	6,0308	1,24E-09	6,32E-07	435
GO:0099081 supramolecular polymer	1,24E-09	6,0308	1,24E-09	6,32E-07	435
GO:0099512 supramolecular fiber	1,83E-09	5,963975	1,83E-09	8,44E-07	434
GO:0005861 troponin complex	8,57E-09	6,964295	8,57E-09	3,51E-06	25
GO:0044391 ribosomal subunit	9,15E-09	5,901812	9,15E-09	3,51E-06	142
GO:0005840 ribosome	2,03E-08	5,663013	2,03E-08	7,18E-06	179
GO:0003009 skeletal muscle contraction	2,56E-08	6,543129	2,56E-08	8,42E-06	28
GO:0050879 multicellular organismal movement	4,14E-08	6,29183	4,14E-08	1,19E-05	31
GO:0050881 musculoskeletal movement	4,14E-08	6,29183	4,14E-08	1,19E-05	31
GO:0006397 mRNA processing	9,51E-08	5,287186	9,51E-08	2,58E-05	292
GO:0009199 ribonucleoside triphosphate metabolic process	1,12E-07	5,307604	1,12E-07	2,87E-05	143
GO:0009205 purine ribonucleoside triphosphate metabolic process	1,39E-07	5,273186	1,39E-07	3,37E-05	134
GO:0009144 purine nucleoside triphosphate metabolic process	1,58E-07	5,245025	1,58E-07	3,64E-05	136
GO:0006936 muscle contraction	1,9E-07	5,267732	1,9E-07	4,17E-05	117
GO:0098798 mitochondrial protein complex	3,07E-07	5,097474	3,07E-07	6,42E-05	198
GO:0003012 muscle system process	3,95E-07	5,101369	3,95E-07	7,9E-05	124
GO:0009141 nucleoside triphosphate metabolic process	4,26E-07	5,027818	4,26E-07	8,18E-05	152
GO:0044455 mitochondrial membrane part	6,54E-07	4,952215	6,54E-07	0,00012	163
GO:0044445 cytosolic part	1,05E-06	4,843757	1,05E-06	0,000186	159
GO:0008380 RNA splicing	2,22E-06	4,653209	2,22E-06	0,000379	237
GO:0046034 ATP metabolic process	3,84E-06	4,57801	3,84E-06	0,000631	117
GO:0022626 cytosolic ribosome	4,99E-06	4,59203	4,99E-06	0,000792	87
GO:0070469 respiratory chain	7,28E-06	4,524333	7,28E-06	0,001117	75
GO:0030018 Z disc	8,43E-06	4,457722	8,43E-06	0,001252	74
GO:0006941 striated muscle contraction	8,99E-06	4,583173	8,99E-06	0,001294	50

GO:0006412 translation	9,52E-06	4,3053	9,52E-06	0,001328	412
GO:0098800 inner mitochondrial membrane protein complex	1,04E-05	4,375027	1,04E-05	0,001408	109
GO:0031674 I band	1,09E-05	4,381794	1,09E-05	0,001431	83
GO:0044427 chromosomal part	1,14E-05	4,259254	1,14E-05	0,001462	461
GO:0016071 mRNA metabolic process	1,19E-05	4,253874	1,19E-05	0,001484	391
GO:0043043 peptide biosynthetic process	1,4E-05	4,216929	1,4E-05	0,001692	417
GO:0015934 large ribosomal subunit	1,43E-05	4,383207	1,43E-05	0,001692	83
GO:0009123 nucleoside monophosphate metabolic process	1,58E-05	4,221616	1,58E-05	0,001822	162
GO:0000003 reproduction	1,65E-05	4,189676	1,65E-05	0,001855	255
GO:0006518 peptide metabolic process	1,71E-05	4,164812	1,71E-05	0,001873	491
GO:0005179 hormone activity	2E-05	4,189538	2E-05	0,002146	115
GO:0017144 drug metabolic process	2,38E-05	4,091858	2,38E-05	0,002436	365
GO:0009161 ribonucleoside monophosphate metabolic process	2,38E-05	4,124877	2,38E-05	0,002436	158
GO:0006091 generation of precursor metabolites and energy	2,44E-05	4,113422	2,44E-05	0,002439	176
GO:0098803 respiratory chain complex	2,62E-05	4,224879	2,62E-05	0,002517	67
GO:0000375 RNA splicing, via transesterification reactions	2,73E-05	4,09329	2,73E-05	0,002517	187
GO:0000377 RNA splicing, via transesterification reactions	2,73E-05	4,09329	2,73E-05	0,002517	187
GO:0000398 mRNA splicing, via spliceosome	2,73E-05	4,09329	2,73E-05	0,002517	187
GO:0005746 mitochondrial respiratory chain	2,8E-05	4,204058	2,8E-05	0,002523	66
GO:0022414 reproductive process	2,85E-05	4,060517	2,85E-05	0,002523	251
GO:0009126 purine nucleoside monophosphate metabolic process	3,03E-05	4,070776	3,03E-05	0,002585	145
GO:0009167 purine ribonucleoside monophosphate metabolic process	3,03E-05	4,070776	3,03E-05	0,002585	145
GO:0019866 organelle inner membrane	3,54E-05	4,009657	3,54E-05	0,002968	259
GO:0006281 DNA repair	3,89E-05	3,984296	3,89E-05	0,003201	286
GO:0009201 ribonucleoside triphosphate biosynthetic process	4,36E-05	4,031523	4,36E-05	0,003526	79
GO:0006259 DNA metabolic process	4,57E-05	3,931776	4,57E-05	0,003627	460
GO:0005743 mitochondrial inner membrane	4,76E-05	3,941233	4,76E-05	0,003712	232
GO:0045214 sarcomere organization	5,02E-05	4,062879	5,02E-05	0,003849	57
GO:0044429 mitochondrial part	5,14E-05	3,901409	5,14E-05	0,003879	475
GO:0009145 purine nucleoside triphosphate biosynthetic process	5,54E-05	3,984612	5,54E-05	0,004049	70
GO:0009206 purine ribonucleoside triphosphate biosynthetic process	5,54E-05	3,984612	5,54E-05	0,004049	70
GO:0022625 cytosolic large ribosomal subunit	5,81E-05	4,160117	5,81E-05	0,004184	47
GO:0044451 nucleoplasm part	6,86E-05	3,834049	6,86E-05	0,004862	413
GO:0051321 meiotic cell cycle	7,82E-05	3,862158	7,82E-05	0,005438	101
GO:0031033 myosin filament organization	8,15E-05	4,084641	8,15E-05	0,005438	28
GO:0031034 myosin filament assembly	8,15E-05	4,084641	8,15E-05	0,005438	28
GO:0071688 striated muscle myosin thick filament assembly	8,15E-05	4,084641	8,15E-05	0,005438	28
GO:0015935 small ribosomal subunit	8,86E-05	3,954587	8,86E-05	0,00577	59
GO:0009259 ribonucleotide metabolic process	8,9E-05	3,775155	8,9E-05	0,00577	267
GO:0019693 ribose phosphate metabolic process	9,03E-05	3,770041	9,03E-05	0,005778	279
GO:0015078 proton transmembrane transporter activity	9,44E-05	3,796702	9,44E-05	0,005957	113
GO:0009142 nucleoside triphosphate biosynthetic process	0,000114	3,770568	0,000114	0,007079	85
GO:0031672 A band	0,000116	3,917425	0,000116	0,0071	35
GO:0051726 regulation of cell cycle	0,000119	3,692054	0,000119	0,007231	412
GO:0009150 purine ribonucleotide metabolic process	0,000129	3,680693	0,000129	0,007709	252
GO:0030240 skeletal muscle thin filament assembly	0,000156	3,884542	0,000156	0,009192	28

GO:0030241 skeletal muscle myosin thick filament assembly	0,000158	3,889877	0,000158	0,009192	27
KEGG					
dre03010 Ribosome	7,59E-10	6,444811	7,59E-10	1,15E-07	127
dre00190 Oxidative phosphorylation	3,70E-08	5,574204	3,70E-08	2,79E-06	134
dre03040 Spliceosome	0,000233	3,558233	0,000233	0,011711	131
dre01200 Carbon metabolism	0,000357	3,425732	0,000357	0,013459	131
dre00010 Glycolysis / Gluconeogenesis	0,001543	3,007828	0,001543	0,046611	76
dre00983 Drug metabolism - other enzymes	0,001959	2,941778	0,001959	0,049312	61
Stress vs. Control downregulated					
GO					
GO:0004713 protein tyrosine kinase activity	2,73E-07	-5,14243	2,73E-07	0,001048	127
GO:0043235 receptor complex	4,55E-07	-4,96885	4,55E-07	0,001048	269
GO:0043207 response to external biotic stimulus	5,94E-06	-4,42465	5,94E-06	0,006837	256
GO:0051707 response to other organism	5,94E-06	-4,42465	5,94E-06	0,006837	256
GO:0009607 response to biotic stimulus	8,13E-06	-4,35321	8,13E-06	0,007487	259
GO:0009615 response to virus	1,29E-05	-4,36962	1,29E-05	0,009014	67
GO:0007169 transmembrane receptor protein tyrosine kinase signaling pathway	1,37E-05	-4,22875	1,37E-05	0,009014	284
GO:0019199 transmembrane receptor protein kinase activity	1,73E-05	-4,24773	1,73E-05	0,009953	91
KEGG					
dre04010 MAPK signaling pathway	4,24E-07	-4,96907	4,24E-07	6,4E-05	349
dre04621 NOD-like receptor signaling pathway	0,000148	-3,66037	0,000148	0,011205	154
dre04620 Toll-like receptor signaling pathway	0,00026	-3,53213	0,00026	0,013066	95
dre04744 Phototransduction	0,000359	-3,56559	0,000359	0,013567	36
Fluoxetine vs. Control upregulated					
GO					
GO:0030017 sarcomere	1,17E-07	5,333424	1,17E-07	0,000226	131
GO:0044449 contractile fiber part	1,46E-07	5,285806	1,46E-07	0,000226	132
GO:0030016 myofibril	1,62E-07	5,263313	1,62E-07	0,000226	133
GO:0043292 contractile fiber	2,02E-07	5,216665	2,02E-07	0,000226	134
GO:0009144 purine nucleoside triphosphate metabolic process	3,38E-07	5,092253	3,38E-07	0,000226	136
GO:0009199 ribonucleoside triphosphate metabolic process	3,69E-07	5,068422	3,69E-07	0,000226	143
GO:0009205 purine ribonucleoside triphosphate metabolic process	3,98E-07	5,059764	3,98E-07	0,000226	134
GO:0006163 purine nucleotide metabolic process	4,31E-07	4,981415	4,31E-07	0,000226	262
GO:0019693 ribose phosphate metabolic process	4,87E-07	4,953079	4,87E-07	0,000226	279
GO:0009259 ribonucleotide metabolic process	6,79E-07	4,887383	6,79E-07	0,000226	267
GO:0006936 muscle contraction	6,9E-07	4,97093	6,9E-07	0,000226	117
GO:0009126 purine nucleoside monophosphate metabolic process	7,34E-07	4,919859	7,34E-07	0,000226	145
GO:0009167 purine ribonucleoside monophosphate metabolic process	7,34E-07	4,919859	7,34E-07	0,000226	145
GO:0006091 generation of precursor metabolites and energy	7,39E-07	4,901995	7,39E-07	0,000226	176
GO:0009150 purine ribonucleotide metabolic process	8,2E-07	4,851689	8,2E-07	0,000226	252
GO:0005865 striated muscle thin filament	8,7E-07	5,11086	8,7E-07	0,000226	54
GO:0036379 myofilament	8,7E-07	5,11086	8,7E-07	0,000226	54
GO:0009123 nucleoside monophosphate metabolic process	8,83E-07	4,870419	8,83E-07	0,000226	162
GO:0034660 ncRNA metabolic process	1,16E-06	4,777442	1,16E-06	0,000276	332
GO:0003012 muscle system process	1,2E-06	4,8407	1,2E-06	0,000276	124
GO:0009161 ribonucleoside monophosphate metabolic process	1,26E-06	4,796776	1,26E-06	0,000276	158

GO:0072521 purine-containing compound metabolic process	1,32E-06	4,74342	1,32E-06	0,000276	298
GO:0003735 structural constituent of ribosome	2,25E-06	4,727254	2,25E-06	0,000451	152
GO:0009117 nucleotide metabolic process	2,67E-06	4,586984	2,67E-06	0,000513	346
GO:0046034 ATP metabolic process	3,03E-06	4,633455	3,03E-06	0,000558	117
GO:0006753 nucleoside phosphate metabolic process	3,6E-06	4,521899	3,6E-06	0,000638	350
GO:0022613 ribonucleoprotein complex biogenesis	3,79E-06	4,530983	3,79E-06	0,000647	308
GO:0044391 ribosomal subunit	4,1E-06	4,599033	4,1E-06	0,000675	142
GO:0098798 mitochondrial protein complex	4,92E-06	4,503932	4,92E-06	0,000781	198
GO:0009141 nucleoside triphosphate metabolic process	6,48E-06	4,434776	6,48E-06	0,000994	152
GO:0044455 mitochondrial membrane part	7,59E-06	4,415163	7,59E-06	0,001128	163
GO:0003009 skeletal muscle contraction	9,42E-06	4,776851	9,42E-06	0,00132	28
GO:0006412 translation	9,46E-06	4,309842	9,46E-06	0,00132	412
GO:0050879 multicellular organismal movement	1,18E-05	4,652965	1,18E-05	0,001554	31
GO:0050881 musculoskeletal movement	1,18E-05	4,652965	1,18E-05	0,001554	31
GO:0055086 nucleobase-containing small molecule metabolic process	1,3E-05	4,230349	1,3E-05	0,00166	415
GO:0034470 ncRNA processing	1,37E-05	4,245744	1,37E-05	0,001665	254
GO:0006941 striated muscle contraction	1,37E-05	4,431813	1,37E-05	0,001665	50
GO:0007601 visual perception	1,56E-05	4,238356	1,56E-05	0,001837	147
GO:0005840 ribosome	1,63E-05	4,228984	1,63E-05	0,001875	179
GO:0043043 peptide biosynthetic process	1,98E-05	4,138762	1,98E-05	0,002219	417
GO:0015980 energy derivation by oxidation of organic compounds	2,13E-05	4,185421	2,13E-05	0,00233	123
GO:0050953 sensory perception of light stimulus	2,46E-05	4,123614	2,46E-05	0,002636	154
GO:0006518 peptide metabolic process	2,53E-05	4,073881	2,53E-05	0,002644	491
GO:0070469 respiratory chain	3,16E-05	4,165479	3,16E-05	0,00323	75
GO:0009127 purine nucleoside monophosphate biosynthetic process	3,59E-05	4,07192	3,59E-05	0,003516	85
GO:0009168 purine ribonucleoside monophosphate biosynthetic process	3,59E-05	4,07192	3,59E-05	0,003516	85
GO:0043604 amide biosynthetic process	3,87E-05	3,973568	3,87E-05	0,003712	480
GO:0009124 nucleoside monophosphate biosynthetic process	4,47E-05	3,999043	4,47E-05	0,00415	101
GO:0017144 drug metabolic process	4,51E-05	3,938214	4,51E-05	0,00415	365
GO:0042254 ribosome biogenesis	5,55E-05	3,917606	5,55E-05	0,005014	205
GO:0030018 Z disc	5,89E-05	3,97508	5,89E-05	0,005215	74
GO:0009156 ribonucleoside monophosphate biosynthetic process	6,16E-05	3,919026	6,16E-05	0,005352	98
GO:0006164 purine nucleotide biosynthetic process	6,51E-05	3,874823	6,51E-05	0,005555	156
GO:0098800 inner mitochondrial membrane protein complex	6,72E-05	3,911589	6,72E-05	0,005629	109
GO:0009260 ribonucleotide biosynthetic process	7,46E-05	3,837522	7,46E-05	0,006137	164
GO:0009152 purine ribonucleotide biosynthetic process	7,62E-05	3,836548	7,62E-05	0,006157	150
GO:0046390 ribose phosphate biosynthetic process	7,93E-05	3,821061	7,93E-05	0,006297	167
GO:0009201 ribonucleoside triphosphate biosynthetic process	8,25E-05	3,861156	8,25E-05	0,006438	79
GO:0009145 purine nucleoside triphosphate biosynthetic process	9,11E-05	3,847039	9,11E-05	0,006879	70
GO:0009206 purine ribonucleoside triphosphate biosynthetic process	9,11E-05	3,847039	9,11E-05	0,006879	70
GO:0098803 respiratory chain complex	0,000107	3,854088	0,000107	0,007957	67
GO:0019752 carboxylic acid metabolic process	0,00011	3,708618	0,00011	0,008036	500
GO:0045333 cellular respiration	0,000127	3,752971	0,000127	0,009139	88
GO:0005861 troponin complex	0,000129	3,998797	0,000129	0,009139	25
GO:0072522 purine-containing compound biosynthetic process	0,000135	3,682199	0,000135	0,009406	168
GO:0005746 mitochondrial respiratory chain	0,000138	3,779315	0,000138	0,009497	66

KEGG					
dre00190 Oxidative phosphorylation	2,52E-06	4,679714	2,52E-06	0,000198	134
dre03010 Ribosome	2,62E-06	4,725296	2,62E-06	0,000198	127
dre04744 Phototransduction	7,92E-05	4,016019	7,92E-05	0,003691	36
dre01200 Carbon metabolism	9,78E-05	3,779534	9,78E-05	0,003691	131
dre00010 Glycolysis / Gluconeogenesis	0,000833	3,202248	0,000833	0,025153	76
dre03040 Spliceosome	0,001156	3,088301	0,001156	0,029099	131
Fluoxetine vs. Control downregulated					
GO					
GO:0006955 immune response	7,61E-07	-4,84991	7,61E-07	0,003505	423
Fluoxetine vs. Stress upregulated					
GO					
GO:0007601 visual perception	7,36E-08	5,440696	7,36E-08	0,000271	147
GO:0050953 sensory perception of light stimulus	1,18E-07	5,335551	1,18E-07	0,000271	154
GO:0005261 cation channel activity	4,77E-06	4,464657	4,77E-06	0,005886	313
GO:0001653 peptide receptor activity	5,11E-06	4,483654	5,11E-06	0,005886	162
GO:0050877 nervous system process	6,56E-06	4,388221	6,56E-06	0,005946	419
GO:0005216 ion channel activity	7,75E-06	4,347982	7,75E-06	0,005946	408
KEGG					
dre04010 MAPK signaling pathway	5,6E-08	5,362301	5,6E-08	8,45E-06	349
dre04744 Phototransduction	1,29E-05	4,710578	1,29E-05	0,000971	36
Fluoxetine vs. Stress downregulated					
GO					
GO:0099080 supramolecular complex	9,16E-07	-4,80511	9,16E-07	0,001996	435
GO:0099081 supramolecular polymer	9,16E-07	-4,80511	9,16E-07	0,001996	435
GO:0099512 supramolecular fiber	1,3E-06	-4,73273	1,3E-06	0,001996	434
GO:0044427 chromosomal part	1,76E-06	-4,66885	1,76E-06	0,002025	461
GO:0005865 striated muscle thin filament	3,46E-06	-4,79302	3,46E-06	0,002657	54
GO:0036379 myofilament	3,46E-06	-4,79302	3,46E-06	0,002657	54
GO:0005861 troponin complex	4,07E-06	-5,14199	4,07E-06	0,002676	25
GO:0043292 contractile fiber	8,54E-06	-4,39165	8,54E-06	0,004915	134
GO:0030016 myofibril	1,07E-05	-4,33912	1,07E-05	0,005311	133
GO:0007059 chromosome segregation	1,18E-05	-4,30405	1,18E-05	0,005311	151
GO:0044449 contractile fiber part	1,27E-05	-4,29935	1,27E-05	0,005311	132
GO:0003735 structural constituent of ribosome	1,57E-05	-4,26674	1,57E-05	0,005614	152
GO:0030017 sarcomere	1,58E-05	-4,24659	1,58E-05	0,005614	131
KEGG					
dre03010 Ribosome	1,37E-05	-4,32619	1,37E-05	0,002074	127

Supplementary Table S10. Statistical data for qualitative real-time polymerase chain reaction results of the last (fifth) week of CUS showing differential expression of selected four reference genes with significant expression differences detected previously by the RNA-seq. Data is analyzed and represented using the Pfaffl method. Post-hoc Dunn's test for pairwise comparisons for significant Kruskal-Wallis data (see also Figure 6 in the MS for visual representation).

**Group**	**Pfaffl Mean±SEM**	**KW test**	**P value**	**Dunn's test**	
		**saga**			
Control	1.461±0.4057	**8.302**	**0.0157**	C vs. S	0.1344
Stress	0.6749±0.1122			C vs. F	>0.9999
Fluoxetine	1.901±0.47			**S vs. F**	**0.0156**
		**isg15**			
Control	2.384±0.7881	**21.6**	**<0.0001**	**C vs. S**	**<0.0001**
Stress	0.0229±0.007507			**C vs. F**	**0.0277**
Fluoxetine	0.1818±0.06124			S vs. F	0.1264
		**otx5**			
Control	3.013±1.067	**12.71**	**0.0017**	**C vs. S**	**0.0384**
Stress	0.5811±0.2882			C vs. F	>0.9999
Fluoxetine	3.904±1.102			**S vs. F**	**0.0017**
		**tpm4**			
Control	1.09±0.1964	**7.804**	**0.0202**	**C vs. S**	**0.0183**
Stress	2.325±0.4452			C vs. F	>0.9999
Fluoxetine	1.37±0.1431			S vs. F	0.2023

Cited references:

1 Love, M. I., Anders, S. & Huber, W. (2019).

2 Wickham, H. *ggplot2: elegant graphics for data analysis*. (Springer, 2016).

3 Royer, L., Reimann, M., Andreopoulos, B. & Schroeder, M. Unraveling protein networks with power graph analysis. PLoS Comput Biol 4, e1000108 (2008).

4 Luo, W., Friedman, M. S., Shedden, K., Hankenson, K. D. & Woolf, P. J. GAGE: generally applicable gene set enrichment for pathway analysis. *BMC Bioinformatics* **10**, 161, doi:10.1186/1471-2105-10-161 (2009).
